# Genome-wide identification, evolutionary and functional analyses of KFB family members in potato

**DOI:** 10.1186/s12870-022-03611-y

**Published:** 2022-05-02

**Authors:** Ruimin Tang, Haitao Dong, Liheng He, Peng Li, Yuanrui Shi, Qing Yang, Xiaoyun Jia, Xiu-Qing Li

**Affiliations:** 1 College of life sciences, Shanxi Agricultural University, Taigu, 030801 Shanxi China; 2 College of Agriculture, Shanxi Agricultural University, Taigu, 030801 Shanxi China; 3 College of life sciences, Nanjing Agricultural University, Nanjing, 210095 Jiangsu China; 4 Fredericton Research and Development Centre, Agriculture and Agri-Food Canada, Fredericton, New Brunswick E3B 4Z7 Canada

**Keywords:** Potato, *KFB* gene family, Synteny analysis, Expression pattern, Anthocyanin biosynthesis

## Abstract

**Background:**

Kelch repeat F-box (KFB) proteins play vital roles in the regulation﻿ of multitudinous biochemical and physiological processes in plants, including growth and development, stress response and secondary metabolism. Multiple KFBs have been characterized in various plant species, but the family members and functions have not been systematically identified and analyzed in potato.

**Results:**

Genome and transcriptome analyses of *StKFB* gene family were conducted to dissect the structure, evolution and function of the StKFBs in *Solanum tuberosum* L. Totally, 44 StKFB members were identified and were classified into 5 groups. The chromosomal localization analysis showed that the 44 *StKFB* genes were located on 12 chromosomes of potato. Among these genes, two pairs of genes (*StKFB15*/*16* and *StKFB40*/*41*) were predicted to be tandemly duplicated genes, and one pair of genes (*StKFB15*/*29*) was segmentally duplicated genes. The syntenic analysis showed that the *KFBs* in potato were closely related to the *KFBs* in tomato and pepper. Expression profiles of the *StKFBs* in 13 different tissues and in potato plants with different treatments uncovered distinct spatial expression patterns of these genes and their potential roles in response to various stresses, respectively. Multiple *StKFB* genes were differentially expressed in yellow- (cultivar ‘Jin-16’), red- (cultivar ‘Red rose-2’) and purple-fleshed (cultivar ‘Xisen-8’) potato tubers, suggesting that they may play important roles in the regulation of anthocyanin biosynthesis in potato.

**Conclusions:**

This study reports the structure, evolution and expression characteristics of the KFB family in potato. These findings pave the way for further investigation of functional mechanisms of StKFBs, and also provide candidate genes for potato genetic improvement.

**Supplementary Information:**

The online version contains supplementary material available at 10.1186/s12870-022-03611-y.

## Background

The F-box gene family broadly exists in plants and plays a crucial role in plant growth and development through a ubiquitin-mediated degradation of cellular proteins [1, 2]. F-box proteins are named for the presence of conserved F-box domain, which is generally located at the N-terminus of the protein and functions in coordination with other motifs at the C-terminus [3, 4]. The F-box domain consists of around 50 amino acids and binds to SKP1 (S-phase Kinase-associated Protein 1) or SKP1-like proteins in the SCF (Skp1-Cullin-F-box) complex, which is the most typical E3 (ubiquitin-ligation enzymes) in organisms [4, 5]. The C-terminus usually contains some highly variable secondary motifs that are responsible for the specific recognition and binding of their substrate proteins [1]. F-box proteins are diverse due to their different C-terminal motifs, such as Kelch repeats, leucine-rich repeats, WD-40 repeats and tetratricopeptide repeats that interact with specific proteins through the UPS (ubiquitination-26 s proteasome system) degradation pathway [4, 6, 7].

Kelch repeat F-box (KFB) subfamily is a major category of the F-box protein family and participates in ubiquitin-mediated protein degradation by selective binding of target proteins [1]. The approximately 50 residues of the F-box domain at N-terminus of KFB lack strictly conserved sequences and only a few amino acid residues are relatively invariant. By analyzing the alignment of 234 sequences used to create the F-box Pfam profile (http://pfam.wustl.edu/cgi-bin/getdesc?name=Fbox), Kipreos and Pagano found that the 8th amino acid of F-box domain was mostly leucine (L) or methionine (M); the 9th amino acids was mainly proline (P); the 16th was isoleucine (I) or valine (V); the 20th was leucine (L) or methionine (M), and the 32nd was serine (S) or cysteine (C) [3]. This domain of KFB was used to accurately recognize the core element of SCF and functions in protein degradation via ubiquitylation pathway. Another typical domain of KFB is the Kelch motif, which is a highly evolved but ancient consensus sequence [8]. Sequence alignment of Kelch repeats (supplemental Web data at http://info.med.yale.edu/cooley) showed that the sequence identity between individual Kelch motifs is low, and each Kelch motif is featured with 8 conserved amino acid residues: four hydrophobic amino acids, followed by two adjacent glycines (G), and two non-adjacent aromatic amino acids (Y or W) [9]. ﻿The crystal structure of the Kelch domain of fungal galactose oxidase revealed that multiple Kelch repeats can generate a β-propeller with blades arrayed around a funnel-like central axis [10, 11]. Different numbers of repeated Kelch motifs can generate distinct contact sites and interact with disparate partners, resulting in the diversification of KFB functions [12]. However, the key residues associated with protein contact sites in the β-propeller structures of the vast majority KFBs have not been mapped. Apart from F-box domain and Kelch repeat motifs, some KFB members possess other conserved domains. For example, the LOV (Light, Oxygen or Voltage) domain has been found to exist in N-terminus of some KFB proteins, including ZTL (ZEITLUPE), FKF1 (Flavin-binding Kelch repeat F-box 1) and LKP2 (Light, oxygen or voltage Kelch protein 2) [13]. The presence of the LOV domain in KFB proteins makes their function different from that of other KFB proteins.

With the development of deep sequencing, numerous KFBs have been identified in many plant species, like chickpea (*Cicer arietinum*), *Arabidopsis* (*Arabidopsis thaliana*), salvia (*Salvia miltiorrhiza*), wheat (*Triticum aestivum*) and so on, but only a few KFB members have been functionally characterized in depth [1, 14–16]. KFB proteins have been demonstrated to participate in plant growth and development. For example, CFK1 (COP9 interacting F-box Kelch 1) was proved to participate in hypocotyl elongation under light in *Arabidopsis* [17]. OsFBK12 modulated seed germination and leaf senescence by affecting ethylene levels in rice [18]. In potato, StFKF1 controlled potato tuberization and maturation by affecting the activity of StSP6A, which interacted with StCDF (Cycling Dof Factor) [19, 20]. CTG10 (Cold Temperature Germinating 10), a Kelch F-box protein in *Arabidopsis*, stimulated the seed germination through a negatively regulation of PIF1 (Phytochrome Interacting Factor 1) activity [21]. Furthermore, previous studies have exemplified that large numbers of KFB members played a pivotal role in circadian rhythm regulation and photomorphogenesis. In *Arabidopsis*, one KFB member named AFR (Attenuated Far-red Response) degraded the light signal suppressor and enabled plants to perceive light signals at dawn [22]. ZTL, FKF1, LKP2, as three KFBs with similar structure and function, controlled the photoperiod flowering activity by degrading AtCDFs in *Arabidopsis* [23, 24]. GmZTL3 and GmFKF1 were also demonstrated to regulate flowering process in soybean [25, 26]. Additionally, several KFB members were involved in plant hormone signaling and stress responses. The expression of *SmKFB5* was inhibited in the hairy roots of *Salvia miltiorrhiza* treated with methyl jasmonate (MeJA) [1]. AtKFB39/KMD3 induced by *Meloidogyne incognita* in plant roots can degrade specific target proteins through the formation of SCF^AtKFB39^ complex and thereby promote the successful phagocytosis of pathogens [27]. In recent decades, an increasingly number of studies have focused on the function of KFB proteins in the biosynthesis of secondary metabolites, and great progress has been made. One of CmKFB members in muskmelon was reported to negatively regulate the production and accumulation of naringin chalcone by transferring the metabolic flux of flavonoids [28]. AtKFB^PAL^ and AtKFB^CHS^, post-translationally regulated phenylpropanoid metabolism by mediating protein ubiquitination and degradation of PAL (phenylalanine ammonia-lyase) and CHS (chalcone synthase), respectively, thereby controlling development and stress response in *Arabidopsis thaliana* [14, 29]. The negative role of AtFKF1 in regulation of cellulose biosynthesis was also observed in *Arabidopsis* [30].

Potato (*Solanum tuberosum* L.), originally discovered in the Andes region of South America and initially domesticated in Peru, is considered as a dominant crop closely related to social and economic development [31]. The yield of edible dry matter per unit area of potato has been reported to be almost the same as that of cereal crops [32]. During long period of cultivation in the field and adaptation to extreme environment, potato has gradually accumulated abundant genes for resistance to diversified stresses, including diseases, pests, drought, cold, high salt and so on [33]. Colored potatoes, especially purple fleshed potatoes rich in anthocyanins are favored by many consumers [34, 35]. Despite the importance of potato, the functions and regulatory mechanisms of most StKFBs are still largely unknown in potatoes. KFB family members, as described above, play important roles in plant growth and development, stress responses, and biosynthesis of secondary metabolites. However, the functions and regulatory mechanism of StKFBs has not been systematically reported in potatoes.

In this research, gene members of *StKFB* family were firstly identified from the whole genome of potato. Their sequence characteristics, motif composition, gene structure, evolutionary relationship, duplication events and synteny prediction were comprehensively analyzed. In order to shed light into their underlying functions, the expression profiles of the identified *StKFB* members were examined across various tissues, different treatments, as well as tubers from cultivars containing various levels of anthocyanin content, using in-house and publicly available transcriptome sequencing data. Moreover, the expression patterns of 9 selected *StKFB* genes in the tubers with different colors were analyzed by quantitative real-time polymerase chain reaction (qRT-PCR). These results will enrich the knowledge of structural characteristics, evolutionary relationship and expression patterns of potato KFBs and provide a theoretical basis for further exploration of the functional mechanism of StKFB members.

## Result

### Identification of StKFB members in potato

The profile HMMs (Hidden Markov Models) of F-box domains and Kelch domains were downloaded from Pfam database [36] (Additional file 1: Table S1). Totally, 379 and 45 candidate proteins containing F-box domains and Kelch domains were identified, respectively, by searching the potato protein sequences using HMMER software package v3.0 [37]. Furthermore, 84 StKFB members were identified by alignment against the potato genome (DM v4.03/v4.04) [38] using AtKFB protein sequences from *Arabidopsis* (TAIR10). Totally, 91 StKFBs were preliminarily obtained through these two methods. After removal of redundant and non-full length sequences, 44 StKFB family members were identified (Table 1). These StKFB members were renamed as StKFB01 to StKFB44 based on their chromosomal localizations. Their CDS and protein sequences were presented in Additional file 2.

**Table 1:** The list of StKFB members identified in potato

Proposed name	Protein sequence number in Spud DB Potato Genomics Resources	Chromosomal location	Kelch number	CDS length (bp)	Protein length (amino acid)	MW (KDa)	pI	GRAVY	Subcellular localization
StKFB01	PGSC0003DMP400034658	1:531784–536,380	4	1905	634	70.39	4.80	−0.34	Nucleus
StKFB02	PGSC0003DMP400055280	1:1500584–1,506,897	2	1314	437	49.27	9.64	−0.27	Nucleus
StKFB03	PGSC0003DMP400023231	1:52787118–52,792,578	3	1314	437	49.52	10.02	−0.28	Nucleus
StKFB04	PGSC0003DMP400039281	1:58222546–58,224,447	1	1113	370	42.46	5.66	−0.12	Nucleus
StKFB05	PGSC0003DMP400039629	1:60036805–60,038,756	1	1095	364	42.23	4.77	−0.16	Nucleus
StKFB06	PGSC0003DMP400000361	1:72056268–72,059,559	3	1200	399	44.27	5.16	−0.24	Nucleus
StKFB07	PGSC0003DMP400000190	1:72867862–72,871,090	1	1218	405	45.99	5.27	−0.05	Nucleus
StKFB08	PGSC0003DMP400022250	1:81754156–81,756,635	3	1428	475	52.25	7.52	−0.18	Nucleus
StKFB09	PGSC0003DMP400005740	2:12109437–12,111,893	5	1209	402	45.39	7.95	−0.11	Nucleus
StKFB10	PGSC0003DMP400063161	2:20267166–20,267,906	1	741	246	28.35	8.82	−0.01	Nucleus
StKFB11	PGSC0003DMP400014559	2:29840585–29,841,993	1	1254	417	47.36	9.69	−0.21	Nucleus
StKFB12	PGSC0003DMP400002671	2:45255269–45,257,745	1	1317	438	50.24	8.84	−0.19	Nucleus
StKFB13	PGSC0003DMP400001216	3:56345081–56,346,592	1	1116	371	41.70	8.16	−0.07	Nucleus
StKFB14	PGSC0003DMP400009925	3:58832801–58,834,345	1	1263	420	48.87	8.98	−0.17	Nucleus
StKFB15	PGSC0003DMP400004568	3:59761482–59,763,077	4	1083	360	40.53	5.61	−0.25	Nucleus
StKFB16	PGSC0003DMP400004569	3:59750380–59,751,653	4	1068	355	39.93	5.44	−0.18	Nucleus
StKFB17	PGSC0003DMP400005140	4:446867–451,573	1	405	134	14.50	5.21	−0.08	Chloroplast
StKFB18	PGSC0003DMP400028986	4:54383109–54,386,555	2	1074	357	39.75	7.60	0.07	Nucleus
StKFB19	PGSC0003DMP400001524	4:59149519–59,151,141	2	1134	377	42.74	9.61	−0.17	Chloroplast
StKFB20	PGSC0003DMP400018760	4:66964061–66,965,818	1	1146	381	44.00	8.36	−0.09	Nucleus
StKFB21	PGSC0003DMP400017581	4:71223587–71,225,655	1	1179	392	44.82	7.86	−0.25	Nucleus
StKFB22	PGSC0003DMP400017645	4:71789808–71,793,120	3	1740	579	65.94	8.59	−0.40	Nucleus
StKFB23	PGSC0003DMP400023853	5:9111929–9,113,418	3	1116	371	41,17	5.42	−0.11	Nucleus
StKFB24	PGSC0003DMP400056401	5:14146752–14,152,580	1	1401	466	52.44	9.31	−0.08	Cell membrane, Chloroplast, Nucleus
StKFB25	PGSC0003DMP400023213	5:49049885–49,051,148	2	1104	367	42.70	6.78	−0.25	Nucleus
StKFB26	PGSC0003DMP400009438	6:1250211–1,254,852	3	1038	345	38.47	6.84	0.03	Nucleus
StKFB27	PGSC0003DMP400046057	6:48061565–48,065,951	6	1401	466	51.70	7.55	−0.12	Nucleus
StKFB28	PGSC0003DMP400013675	6:47941457–47,942,626	1	1170	389	45.41	8.51	−0.24	Nucleus
StKFB29	PGSC0003DMP400028932	6:49430214–49,431,826	2	1137	378	42.14	4.87	−0.16	Nucleus
StKFB30	PGSC0003DMP400034842	6:58465264–58,471,072	1	1851	616	70.05	8.80	−0.28	Nucleus
StKFB31	PGSC0003DMP400014971	7:7499230–7,502,018	3	1254	417	47.36	6.51	−0.48	Nucleus
StKFB32	PGSC0003DMP400063862	7:8301782–8,302,723	1	942	313	36.33	6.72	−0.16	Nucleus
StKFB33	PGSC0003DMP400032228	8:3886902–3,888,342	1	1158	385	43.66	5.12	−0.14	Nucleus
StKFB34	PGSC0003DMP400013056	8:40812188–40,813,595	1	1257	418	47.52	4.57	−0.31	Nucleus
StKFB35	PGSC0003DMP400032959	8:45856649–45,858,089	1	1182	393	43.21	8.87	−0.14	Nucleus
StKFB36	PGSC0003DMP400033574	9:47922617–47,936,286	3	1038	345	38.70	6.88	0.05	Nucleus
StKFB37	PGSC0003DMP400054724	9:49658888–49,663,619	4	1254	417	46.58	8.26	−0.12	Nucleus
StKFB38	PGSC0003DMP400068902	9:58480722–58,481,828	1	1107	368	42.27	8.20	−0.06	Nucleus
StKFB39	PGSC0003DMP400041016	10:57368001–57,369,328	2	1032	343	38.14	6.25	−0.33	Nucleus
StKFB40	PGSC0003DMP400014200	11:41051214–41,055,813	1	1137	378	44.3	9.10	−0.11	Nucleus
StKFB41	PGSC0003DMP400014281	11:41047198–41,048,310	1	1113	370	43.11	8.60	−0.11	Chloroplast
StKFB42	PGSC0003DMP400047693	11:43665296–43,666,731	1	1179	392	42.56	8.73	0.09	Nucleus
StKFB43	PGSC0003DMP400057355	12:4312012–4,313,079	2	1068	355	41.23	8.45	−0.21	Nucleus
StKFB44	PGSC0003DMP400048465	12:9079836–9,080,918	1	1083	360	41.73	7.92	−0.21	Nucleus

*MW* Molecular weight, *pI* Isoelectric point, *GRAVY* Grand average of hydropathicity, is defined as the ratio of the sum of hydrophilic values of all amino acids in a sequence to the number of amino acids. GRAVY > 0 represents that these amino acids were hydrophobic, and the higher score, the stronger the hydrophobicity; GRAVY < 0 shows that these amino acids were hydrophilic, and the lower score, the stronger the hydrophilicity

The CDS length of the candidate *StKFBs* ranged from 405 bp (*StKFB17*) to 1905 bp (*StKFB01*), encoding 134 to 634 amino acids. Molecular weight (MW) of the deduced StKFB proteins varied from 14.5 KDa (StKFB17) to 70.39 KDa (StKFB01). Of these 44 StKFB members, most of them contained a single Kelch motif (23 members), followed by the members contained 3 Kelch motifs (8 members), 2 Kelch motifs (7 members), 4 Kelch motifs (4 members), 5 Kelch motifs (1 members) and 6 Kelch motifs (1 member). The differences in Kelch motif numbers in StKFBs revealed their structural complexity and functional diversity. The theoretical isoelectric point (pI) of the StKFBs widely ranged from 4.8 (StKFB01) to 10.02 (StKFB03), suggesting that these KFB proteins may distribute and function in different microenvironments of cells. The prediction of subcellular localization showed that the majority of StKFBs were located in nucleus, and only a few members exist in chloroplast (StKFB17, StKFB19, StKFB24 and StKFB41) and cell membrane (StKFB24). The grand average of hydropathicity (GRAVY) data indicated that most StKFBs may belong to hydrophilic proteins except StKFB18, StKFB26, StKFB36 and StKFB42.

### Structural analysis of conserved domains in StKFBs

**Fig. 1:**
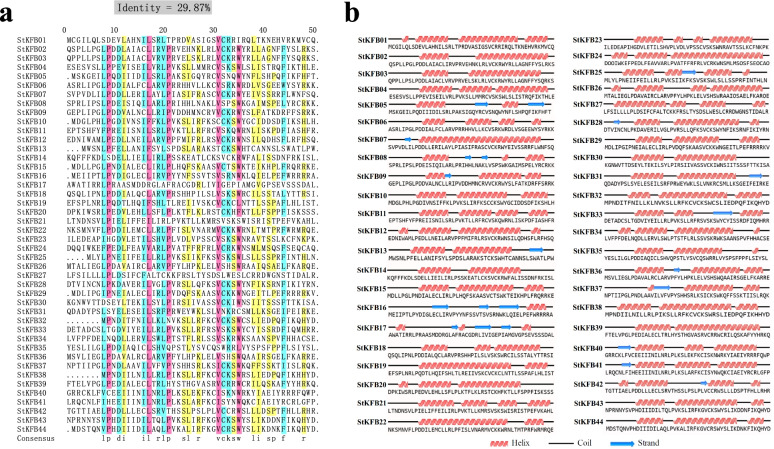
Multi-sequence alignment (**a**) and the secondary structure prediction (**b**) of F-box domains in the identified StKFBs. (**a**) The identity was automatically calculated by multiple sequence alignment of DNAMAN X software. The amino acids with identity greater than 75, 50 and 33% are highlighted in pink, blue and yellow, respectively. (**b**) Secondary structures prediction was performed using online tool provided by NovoPro Bioscience Inc.

The sequences and positions of F-box and Kelch domains in 44 StKFB members were detected using PfamScan database [39] (Additional file 1: Table S2 and Table S3). Multiple sequence alignment analysis of F-box domains showed that the identity of all aligned sequences was 29.87% and these relatively conserved amino acids were discontinuous (Fig. 1a). In this figure, the amino acids labeled in pink, such as proline (P), leucine (L), valine (V) and tryptophan (W) at the 9th, 17th, 31st and 35th position respectively, were the most conserved residues with identity greater than 75%. The amino acids marked in blue and yellow were less conserved, with identity more than 50 and 33%, respectively. Other amino acids without any color shadow had great variation. Furthermore, the secondary structures prediction of F-box domains of StKFBs showed that helices and coils were the main secondary structures, while the strands and coils were dominant in F-box domains of a few StKFB members (Fig. 1b). Such structures may facilitate their interaction with other proteins like SKP1 in their network.

**Fig. 2:**
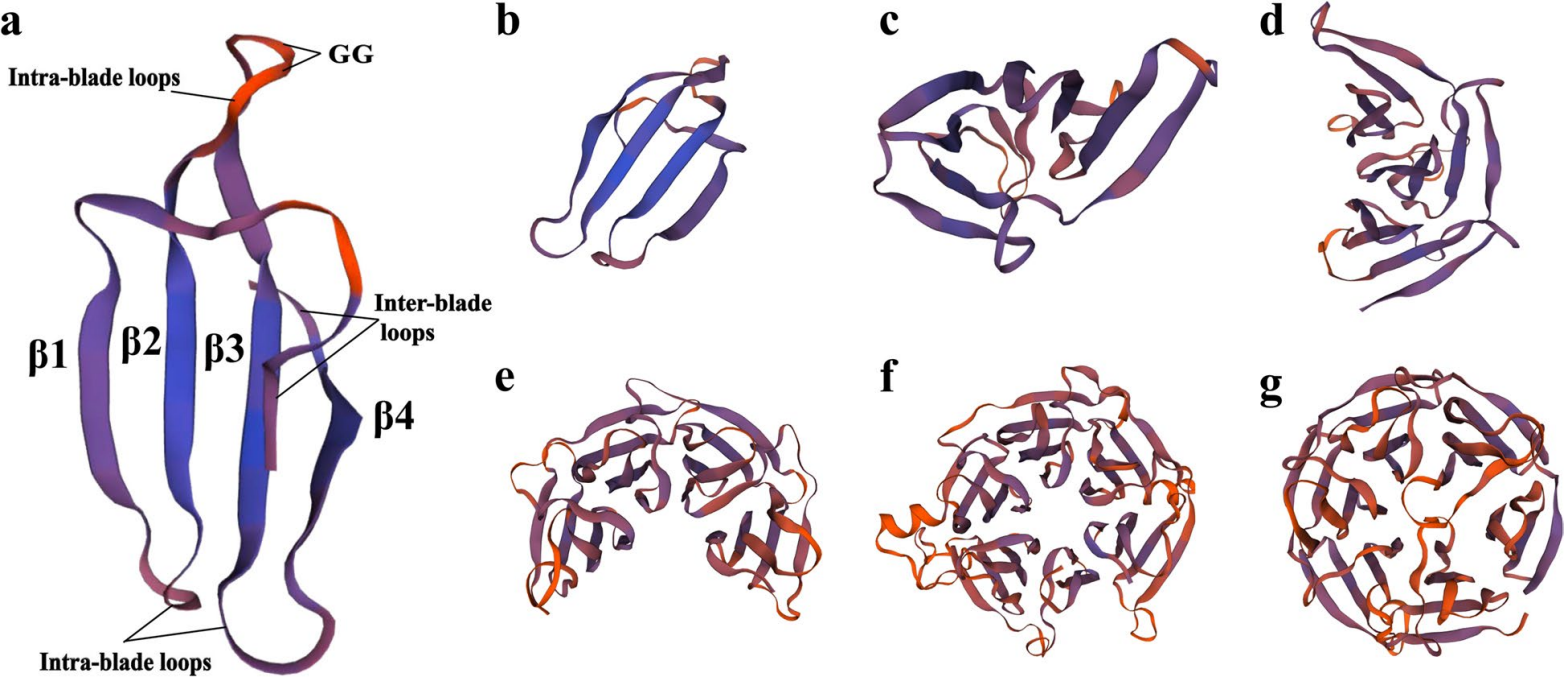
The tertiary structures of Kelch motifs with different numbers. **a** ﻿A typical Kelch motif with a four-stranded β-sheets. **b-g** The tertiary structures of Kelch domains with 1–6 Kelch motifs predicted by SWISS-MODEL website. The Kelch domains were extracted from StKFB07, StKFB02, StKFB03, StKFB01, StKFB09 and StKFB27, respectively

The sequences of Kelch motifs of StKFBs were also variable. The most striking feature of each Kelch repeat was the conserved bi-glycine (GG) and two characteristically spaced aromatic residues (Y or W) (Additional file 1: Table S3). Four inverted β-sheets were spatially twisted into a Kelch motif (Fig. 2a). Multiple Kelch repeats were arranged as blades around a funnel-shaped central axis to form a β propeller structure (Fig. 2b-g). The intra-blade loops connected two adjacent sheets in each Kelch motif; while the inter-blade loops jointed different Kelch motifs. The diversification of spatial structures of Kelch motifs with different numbers implies difference in StKFB functions.

### Gene duplication analyses of *StKFB* genes

**Fig. 3:**
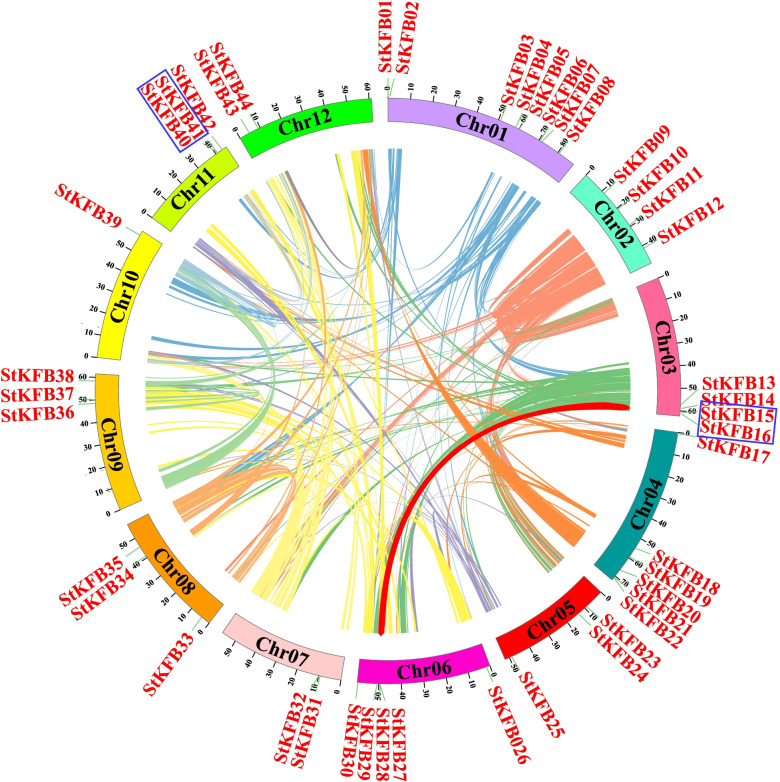
Chromosomal distribution and gene duplication of *StKFB* genes. Chromosomal distribution of *StKFB* genes was conducted by Circos software. Gene duplication events of *StKFBs* were analyzed by MCScanX. Colorized lines inside represent all synteny blocks in the potato genome. The segmentally duplicated genes are linked by the bold red line. Tandem duplications are indicated in blue box

The 44 deduced *StKFBs* were unevenly distributed on 12 potato chromosomes according to chromosomal localization analysis using Circos software [40] (Fig. 3). Relatively more *StKFB* genes were observed on Chr01, Chr04 and Chr06, containing 8, 6 and 5 *StKFBs*, respectively. While *StKFB* genes were less distributed in Chr10, Chr07, Chr12, containing 1, 2 and 2 *StKFB* genes, respectively. Most chromosomes contained 3 (Chr05, Chr08, Chr09 and Chr11) or 4 *StKFB* genes (Chr02 and Chr03).

Analysis of gene duplication events in potato genome manifested that there were 7753 single copy genes, 17,021 dispersed genes, 4269 tandem duplications, 5996 segmental duplications and 2443 adjacent but discontinuous repetitive genes in the potato genome (Additional file 3: Fig. S1). Among the 44 *StKFB* genes, *StKFB15*/*StKFB16* on Chr03 (location: 59850507 Mb/59.861699 Mb) and *StKFB40*/*StKFB41* on Chr11 (location: 41.047198 Mb/41.054665 Mb) were found to be two pairs of tandem duplications (Fig. 3) according to the definition of tandemly duplicated genes [41]. Besides, *StKFB15*/*StKFB29* was predicted to be one pair of segmental duplications, implying that they may have differentiated from the same ancestor gene.

The ratio of the number of non-synonymous substitutions per non-synonymous site (Ka) to the number of synonymous substitutions per synonymous site (Ks) is an effective indicator to test the positive selection pressure after gene duplication and to infer the potential date of duplication events [42]. The Ka/Ks ratios of *StKFB15*/*StKFB16*, *StKFB40*/*StKFB41*, *StKFB15*/*StKFB29* were 0.21, 0.65 and 0.26 (less than 1.0), respectively (Table 2), indicating that these duplicated genes were experienced purification and elimination by natural selection during the evolutionary process. Moreover, the occurrence dates of these duplication events were also estimated according to Shen and Yuan [43]. The earliest divergence time between *StKFB15* and *StKFB16* was around 58.16 million years ago (Mya), while *StKFB40* and *StKFB41* began to diverge from 9.77 Mya. The segmental duplication *StKFB15*/*StKFB29* was found to occur around 28.14 Mya, which was later than the divergence date of *StKFB15* and *StKFB16*.

**Table 2:** Tandemly and segmentally duplicated *StKFB* pairs in potato and inference of duplication time

Gene pairs	Type of gene duplication	Chr. location	Ka	Ks	Ka/Ks	Approximate duplication date (Mya)
StKFB15/StKFB16	Tandem duplication	Chr03	0.370878414	1.744689634	0.212575582	58.15632113
StKFB40/StKFB41	Tandem duplication	Chr11	0.191360755	0.293117307	0.652847	9.7705769
StKFB15/StKFB29	Segmental duplication	Chr3/Chr6	0.216198333	0.844230721	0.256089157	28.141024

Ka/Ks is the ratio of the number of non-synonymous substitutions per non-synonymous site (Ka) to the number of synonymous substitutions per synonymous site (Ks). This ratio is used as indicator to determine the selective pressure or strength on a protein-encoding gene. “Ka/Ks = 1” shows “no selection”, “Ka/Ks < 1” indicates “negative or purifying selection” and “Ka/Ks > 1” shows “positive or Darwinian selection”

### Evolutionary analysis of KFB family members in potato and other plant species

**Fig. 4:**
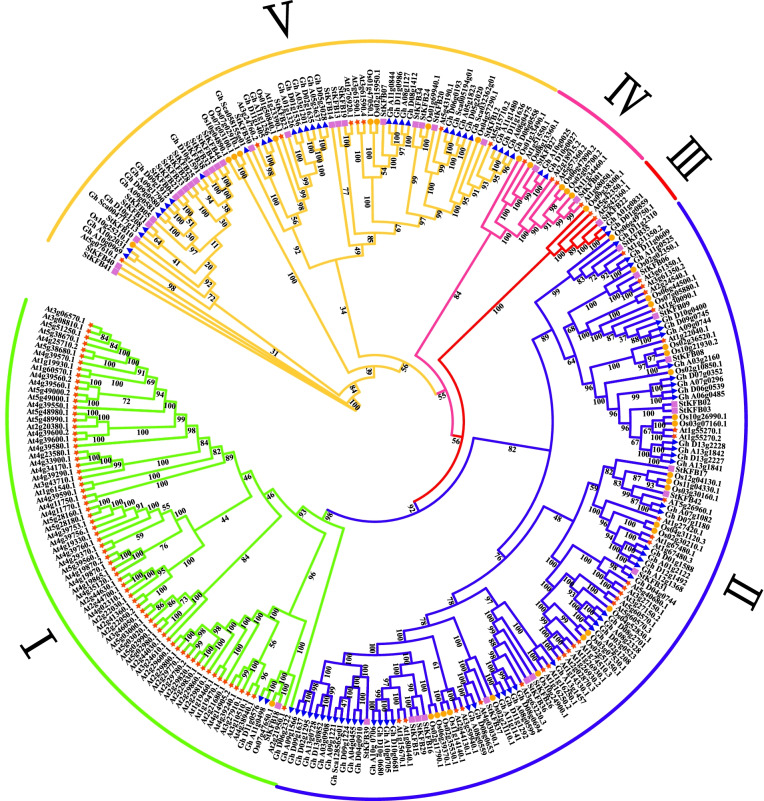
Phylogenetic tree of KFB proteins from potato and other plant species. The maximum- likelihood method of IQ-TREE software was applied to construct the phylogenetic tree with 1000 bootstrap replicates. The model VT + F + R7 was automatically evaluated as the best-fit model through ModelFinder analysis. Branch lines in different colors represent different clans. The abbreviations used for different plant KFB proteins are as follows: At-*Arabidopsis thaliana*, St-*Solanum tuberosum*, Os-*Oryza sativa* and Gh-*Gossypium hirsutum*

To explore the potential evolutionary relationship of KFB proteins in different plant species, a maximum-likelihood (ML) phylogenetic tree was constructed based on the multiple sequence alignment of 284 KFBs, including 44 StKFBs from potato, 115 AtKFBs from *Arabidopsis*, 39 OsKFBs from rice and 86 GhKFBs from upland cotton. As shown in Fig. 4, all the 284 KFB members were classified into five groups, with Group II containing the most members (117 KFBs) and Group III containing the least members (6 KFBs).

The StKFBs in potato were categorized into these five clades according to the classification schemes of other plant species. Group I contained 76 plant KFB members, including 71 AtKFBs, 3 GhKFBs, 1 StKFBs and 1 OsKFB. Large numbers of AtKFB members in Group I implied that KFBs from *Arabidopsis* may have undergone expansion [1, 41]. Group II was the largest clade with a total of 117 plant KFB proteins, containing 48 GhKFBs, 30 AtKFBs, 23 OsKFB and 16 StKFBs. Many KFB members in this group have been functionally studied, such as At1g15670 (AtKFB01) and At1g80440 (AtKFB20) which have been demonstrated to post-translationally regulate phenylpropanoid metabolism [14]. Another AtKFB protein, At2g44130 enhanced nematode susceptibility in *Arabidopsis* [27]. OsFBK12 (Os03g07530) has been reported to play a role in seed germination and leaf senescence of rice [18]. Group III was the smallest clade among the five groups, including 2 AtKFBs, 2 GhKFBs, 1 OsKFB and 1 StKFBs. Group IV was the second smallest group, but the members within the group had distinct characteristics. For example, At5g57360/ZTL, At2g18925/LKP2 and At1g68050/FKF1, which contained LOV motif, were involved in plant circadian rhythm and photomorphogenesis [1]. Group V was composed of 72 KFB members. Most of the potato KFBs (24 members) and 31 upland cotton KFBs were classified into Group V, while KFBs from *Arabidopsis* and rice were less distributed in this group (8 and 9 members respectively). This phylogenetic tree helps to predict the functions of StKFBs that are closely related to those in other plant species.

### Phylogenetic analysis, conserved motifs and exon-intron organization of StKFB family members

**Fig. 5:**
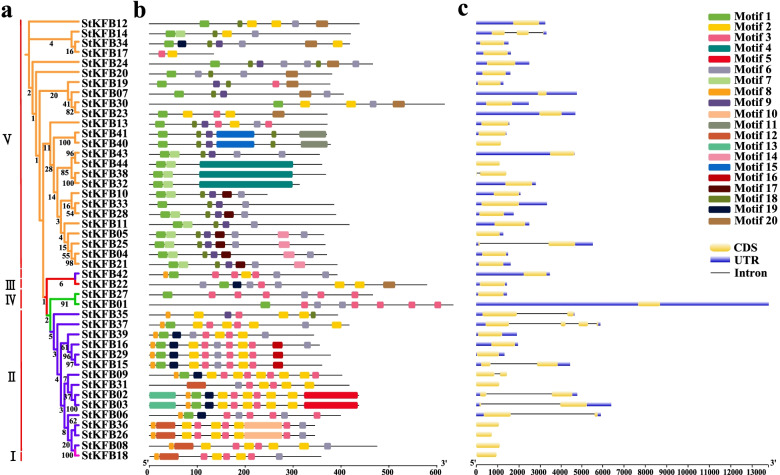
Phylogenetic relationships (**a**), motif composition (**b**) and gene structures **(c)** of StKFB members. (**a**) The phylogenetic tree was constructed by IQ-TREE software using the neighbor-joining method with 1000 bootstrap replicates. The branch lines in different colors represent different KFB groups. (**b**) Motif composition of StKFB proteins was performed by MEME software v5.3.0. The motifs, numbered 1–20, are displayed in different colored boxes. The length of proteins can be estimated using the scale at the bottom. (**c**) Exon/intron distribution of corresponding *StKFB* genes was carried out by GSDS 2.0. The yellow boxes represent exons of genes; the blue boxes indicate untranslated region (UTR); the lines refer to introns of genes. The length of genes can be estimated using the scale at the bottom

The phylogenetic analysis of the 44 StKFB protein sequences was carried out by IQ-TREE [44, 45] to further investigate the evolution relationship of StKFB members in potato. Except for StKFB17, the classification of StKFB members is generally consistent with that in phylogenetic tree among different plant species (Fig. 5a).

Additionally, twenty putative conserved motifs in the 44 StKFB members were identified by MEME software v5.3.0 [46] to investigate the conservation and diversification of structures in StKFB family members (Fig. 5b). The details of the 20 motifs were shown in Additional file 1: Table S4 and Additional file 3: Fig. S2. The motif composition diagram depicted that the numbers of conserved motifs in each KFB protein sequence ranged from 2 to 11 (Fig. 5b). The majority of StKFB members contained Motif 1 (37 members), Motif 2 (23 members), Motif 3 (23 members) and Motif 6 (32 members), suggesting that these motifs are highly conserved in StKFBs. In comparison, several motifs only appeared in a specific group. For instance, Motif 17 and 18 were only distributed in some StKFB members of Group V; while Motif 3 was rarely distributed in Group V. Motif specificity was also shown in tandem and segmental duplications. Motif 11 and 15 were found only in StKFB40 and StKFB41, and Motif 16 was unique to StKFB15, StKFB16 and StKFB29. By annotating the conserved motifs with InterProScan [47, 48], five motifs (Motif 1, 4, 10, 12 and 15) were found as parts of the F-box domains, and four motifs (Motif 2, 3, 5, 13) were considered as Kelch repeats (Additional file 1: Table S4).

Furthermore, the number and length of introns and extrons in *StKFB* genes were analyzed to explore the structural diversity of *StKFB* gene sequences. As shown in Fig. 5c, 34 *StKFB* genes had no introns, while 8, 1 and 1 *StKFBs* contained 1, 2 and 3 introns, respectively. Apart from intron number differences, the length of introns also displayed certain degrees of variation. In comparison with *StKFB09*, *StKFB14* and *StKFB38*, the introns within *StKFB02*, *StKFB06*, *StKFB15*, *StKFB24*, *StKFB25*, *StKFB35* and *StKFB37* were relatively large. Although the gene structures of most closely related genes exhibited high similarity and conservation, there still exist several differences in intron numbers and intron length between some of the phylogenetically related members. *StKFB16* and *StKFB29* had no intron, while *StKFB15* had a long intron, which may result in the expression pattern and function of *StKFB15* being different from that of *StKFB16* and *StKFB29*. Gene structure diversity may have driven the evolution of the *KFB* gene family.

### Syntenic analysis of *KFB* genes in different plant species

**Fig. 6:**
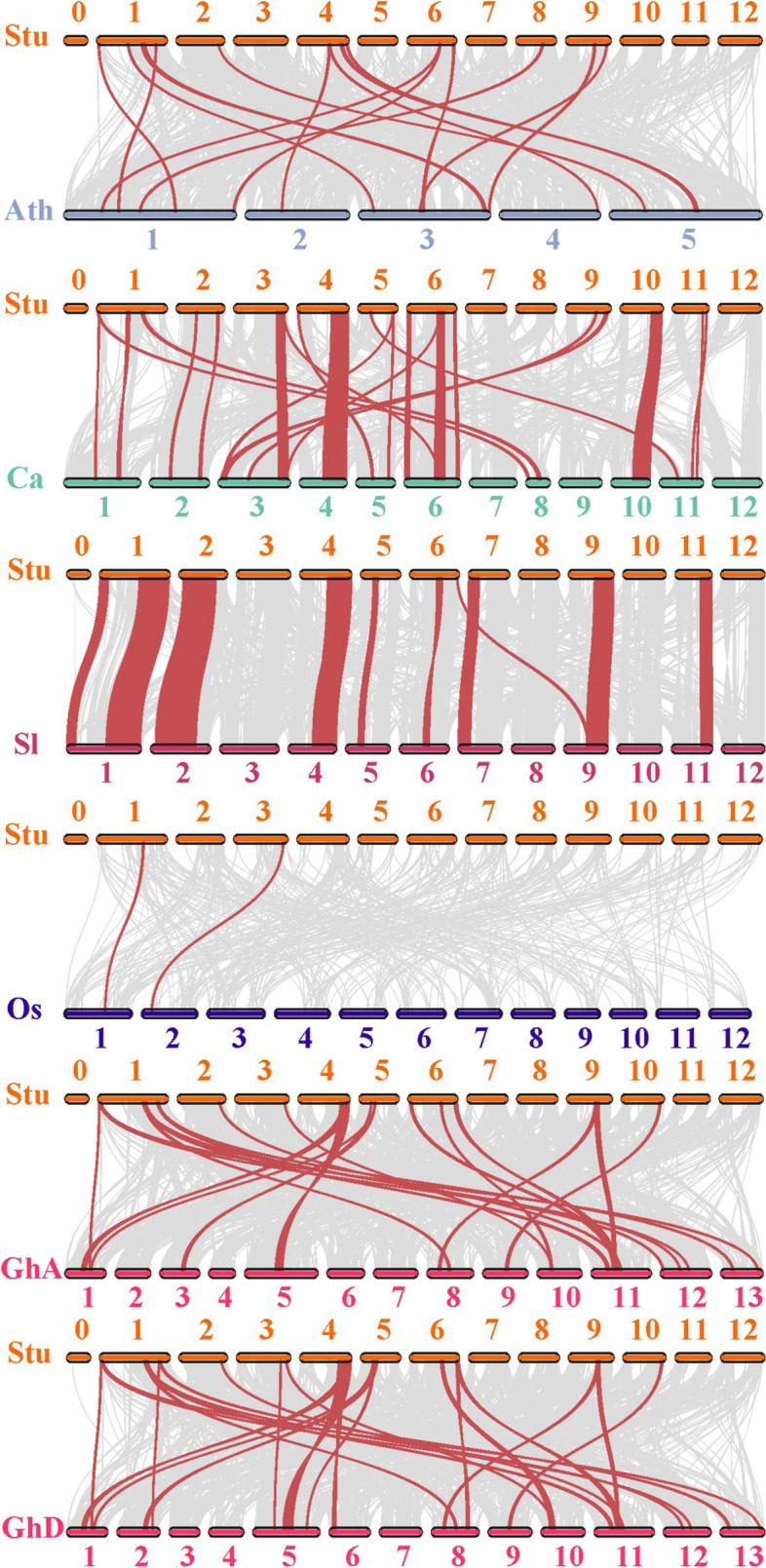
Synteny analysis of *KFB* genes between potato and five other plant species. The syntenic relationship was analyzed by MCScanX software. Gray lines in the background indicate the collinear blocks within potato and other plant genomes, while the red lines highlight the syntenic KFB gene pairs. The abbreviations used for different plant genomes are as follows: Ath-*Arabidopsis thaliana*, Stu-*Solanum tuberosum*, Ca-*Capsicum annuum*, Sl-*Solanum lycopersicum*, Os-*Oryza sativa*, GhA/D-Genome A/D of *Gossypium hirsutum*

Synteny describes the similarity of gene arrangement in different genomes, and to some extent, can represent the evolutionary relationship of genes in different species [49]. To deduce the potential phylogenetic mechanism of *StKFB* genes, the comparative syntenic analysis of *KFB* genes was conducted between potato and five other plant species respectively, including four dicots (*Arabidopsis*, pepper, tomato and upland cotton) and one monocot (rice) (Fig. 6). In general, potato *KFB* genes showed a closer syntenic relationship with those in dicots than the monocot. Totally, 25 potato *KFB* members were found to be syntenic with *KFBs* in pepper, followed by upland cotton (18), tomato (16), *Arabidopsis* (14) and rice (2). The syntenic genes of 5 *StKFB* members (*StKFB02*, *StKFB06*, *StKFB20*, *StKFB22* and *StKFB30*) were all discovered in the genome of these dicots (Additional file 1: Table S5). It is noteworthy that Genome A and Genome D of upland cotton contained 17 syntenic genes of *StKFB* genes, respectively. The syntenic gene of *StKFB26* only existed in Genome A, while the syntenic gene of *StKFB13* was specifically contained in Genome D of upland cotton.

The orthologous *KFB* genes syntenic with *StKFB* genes in other plants were listed in Additional file 1: Table S5. We noticed that the Ka/Ks values of orthologs pairs were less than 1, suggesting that these genes had evolved under the effect of negative or purifying selection. Some *StKFB* genes were syntenic with more than two genes in the genome of pepper, *Arabidopsis* and upload cotton. For example, *StKFB18* in potato was found to be syntenic with two *Arabidopsis KFB* genes (At4g39550.1 and At2g21950.1). Similarly, PHT79419 and PHT88782 in pepper were identified to be the syntenic genes of *StKFB16*. In upland cotton, two genes in Genome A (Gh_A01g1212 and Gh_A12g1407) and one gene in Genome D (Gh_D01g1375) were syntenic with *StKFB01*. These orthologous *KFB* genes in different plants may facilitate KFB family evolution. Moreover, the *KFB* syntenic gene pairs found between potato and other plants were anchored on conserved syntenic blocks. And potato *KFBs* has a larger syntenic blocks with tomato and pepper *KFBs*, indicating that the syntenic relationship of potato *KFB* gene family were closer to tomato and pepper *KFBs* than those in other plants.

### Tissue-specific expression analysis of *StKFB* genes

**Fig. 7:**
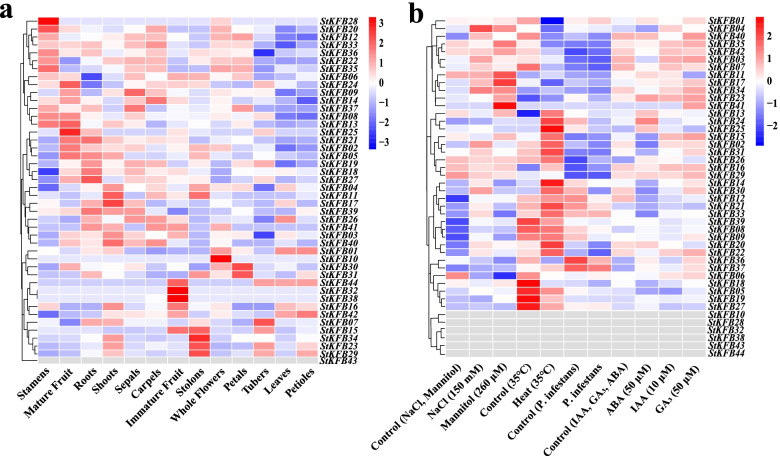
Expression profiles of *StKFB* genes in different potato tissues (**a**) and in potato plants with different treatments (**b**). ﻿The dataset was obtained from the publicly available dataset for FPKM values of all the representative transcripts across 40 DM and 16 RH libraries: DM_RH_RNA-Seq_FPKM_expression_matrix_for_DM_v4.03_13dec2013_desc.xlsx (http://spuddb.uga.edu/pgsc_download.shtml)

The expression heatmap of *StKFB* genes in 13 different potato tissues displayed that the expression levels of individual members of this gene family varied greatly in various tissues (Fig. 7a). Some *StKFB* genes exhibited tissue-specific expression patterns. For example, *StKFB10* and *StKFB28*, two closely related *KFB* genes on the phylogenetic tree, were both predominately expressed in flower organs, suggesting their possible involvement in potato flowering. *StKFB15*/*16*/*32*/*38*/*42*/*44* were mainly expressed in immature fruits; while *StKFB02*/*05*/*08*/*13*/*14*/*21*/*24*/*25* were expressed higher in mature fruits than in immature fruits, inferring that these members might participate in fruit formation and development. Other members such as *StKFB07*/*23*/*29*/*34*, showed high levels of expression in vegetative organs, such as shoots, roots, tubers and stolon, suggesting an involvement of them in plant vegetative growth. In addition, we found that some *StKFBs* with close phylogenetic relationship showed different expression patterns. *StKFB15* and *StKFB16* were predicted to be a pair of tandem duplication, but their expression patterns were not the same. *StKFB15* was mainly expressed in stolon, immature fruits and tubers, while *StKFB16* was highly expressed in shoot and immature fruits. *StKFB29*, the predicted segmentally duplicated gene of *StKFB15*, appeared high expression in stolon, tubers and petioles.

Furthermore, the correlation between the expression patterns of *StKFBs* in diverse tissues was also analyzed. The genes with positive correlation might act synergistically in similar tissues; while the genes with negative correlation might indicate that the function of these members is differentiated [50]. As shown in Additional file 3: Fig. S3a, *StKFB23*, *StKFB29* and *StKFB34*, which were highly expressed in vegetative tissues, had a positive correlation with each other. *StKFB20*/*28*/*35*/*36* with high expression in stamens also showed a high positive correlation. Similarly, *StKFB02*/*05*/*18*/*19*/*21*/*27* were positively correlated and clustered together in the expression heatmap (Fig. 7a and Additional file 3: Fig. S3a). In contrast, *StKFB15*/*23*/*29*/*34* were negatively correlated with *StKFB03*/*26*/*39*/40/41, indicating that these two groups of genes may perform functions in different potato tissues.

### Expression patterns of *StKFBs* in potato plants with different treatments

The RNA-seq data of whole potato plants with various treatments was used to detect the response of *StKFB* genes to different stresses (Additional file 1: Table S6). As shown in Fig. 7b, the *StKFB* genes have different degrees of response to these stresses. The number of up-regulated *StKFBs* induced by salt (150 mM NaCl) and drought stresses (260 μM mannitol) was greater than that of down-regulated members. The expression levels of *StKFB03*/*04*/*07*/*17*/*34* were increased in both salt stress and drought stress, while *StKFB06* showed decreased expression under both treatments compared with the control group. Besides, *StKFB13*/*14*/*15*/*24*/*25*/*30* were predominant *StKFB* transcripts during heat stress (35 °C). Additionally, *StKFBs* were also respond to hormone-induced stresses and *StKFB23* was found to be up-regulated under abscisic acid (ABA), inidole-3-acetic acid (IAA) and gibberellic acid (GA_3_) treatments. Apart from *StKFB23*, exogenous ABA treatment increased the expression levels of *StKFB01*/*13*/*24*/*25*/*26*; while the expression levels of *StKFB04* and *StKFB15* were induced by exogenous IAA treatment. More genes were up-regulated under GA_3_ induction than ABA and IAA induction. For biotic stress, *StKFB13*/*14*/*20*/*24*/*25*/*30* were down-regulated in potato plants infected with *Phytophthora infestans*; while the expression of other members had no significant difference. The expression levels of *StKFB10*/*28*/*32*/*38*/*43*/*44* were too low to be detected in these treatments.

Meanwhile, we analyzed the correlations between the *StKFB* genes expression in potato plants with different treatments (Additional file 3: Fig. S3b). *StKFB08*/*09*/*12*/*21*/*33* showed high positive correlations with each other. *StKFB02*/*20*/*22*, which were highly expressed under salt stress and heat stress, were positively correlated. *StKFB18* was positively correlated with *StKFB05*/*19*/*27*/*39*, but negatively correlated with *StKFB11*/*13*/*16*/*23*/*29*/*34*. *StKFB03*/*11*/*15*/*16*/*26*/*29*/*31*/*34*/*35*/*42* were positively correlated among each other and were negative correlated with *StKFB36* and *StKFB37*. These results might suggest that potato may adaptively respond to harmful environments by mitigating the threat of adversity through coordination and compensation of *StKFB* family members.

### Expression patterns of *StKFB* genes in potato tubers with different colors

**Fig. 8:**
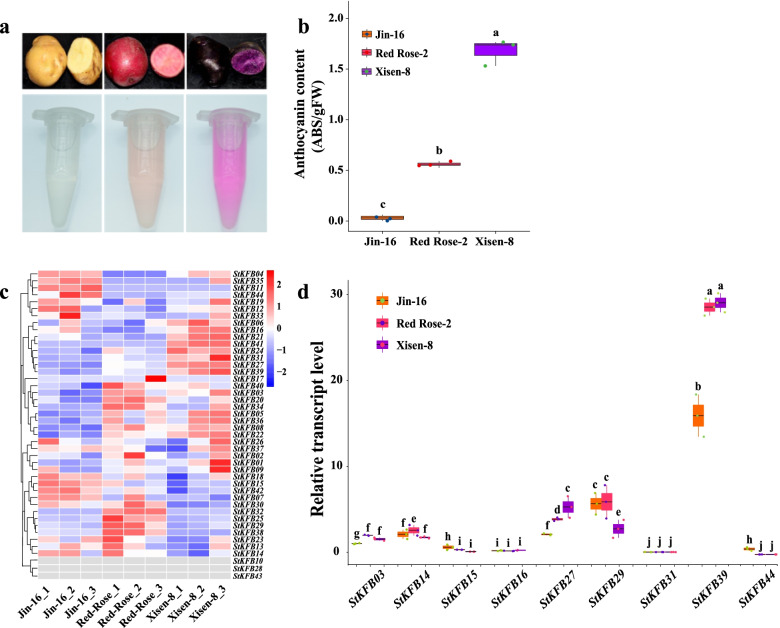
Expression of *StKFB* genes in three colored potato tubers. **a** The appearance, cross profile and anthocyanin extraction of ‘Jin-16’, ‘Red Rose-2’ and ‘Xisen-8’ tubers. **b** Relative anthocyanin content calculated as absorbance at 530 nm/fresh weight (g). **c** Expression profiles of *StKFB* genes in three colored potato tubers detected via in-house transcriptome sequencing data. Each variety had three biological replicates. **d** Expression patterns of 9 selected genes in three colored potato tubers detected by qRT-PCR. Values are means ± SD of three replicates in each experiment. Bars with different lowercase letters represent significant difference at *p* < 0.05

KFB proteins have been demonstrated to regulate phenylpropanoid biosynthesis via degradation of PAL and CHS, the key enzymes in anthocyanin biosynthesis [14, 29]. Therefore, we speculated that StKFBs may be involved in anthocyanins biosynthesis in potato. To explore the roles of *StKFB* genes in anthocyanin biosynthesis, the expression levels of *StKFBs* in potato tubers with different colors were investigated. The skin and flesh of ‘Jin-16’ tubers were yellow in color, while those of ‘Red Rose-2’ and ‘Xisen-8’ were red and dark purple, respectively (Fig. 8a). The anthocyanin contents in the flesh of tubers were also measured. The relative anthocyanin content of tuber flesh in ‘Xisen-8’ was significantly higher than that in ‘Red Rose-2’ (~ 2.7-fold) and ‘Jin-16’ (~ 103.5-fold) (Fig. 8b), suggesting that a different regulatory mechanism related with anthocyanin biosynthesis may exist among the three potato varieties.

The tubers of these three varieties were used as materials for RNA sequencing. After eliminating the low-quality reads, Illumina adapters and reads with unidentifiable base information, the clean reads obtained from each sample accounted for more than 95% of the raw reads (Additional file 1: Table S7). The clean bases generated from transcriptome sequencing were all above 12.00 G. In each sample, the number of filtered reads that could be mapped to the reference genome (DM v4.03/v4.04) made up more than 81% of the total clean reads (Additional file 1: Table S8).

By analyzing the RNA-seq results (Additional file 1: Table S6), we found that *StKFB* gene members were expressed at different levels in these potato cultivars (Fig. 8c). Specifically, *StKFB06*/*16*/*21*/*22*/*24*/*27*/*31*/*39*/*41* were highly expressed in ‘Xisen-8’ compared with the other two cultivars. The expression levels of *StKFB20*/*25*/*29*/*34* were specifically highly expressed in ‘Red Rose-2’. Genes like *StKFB03*/*05*/*08*/*32*/*36*/*40* were highly expressed in both colored potato tubers. The expression levels of *StKFB07*/*11*/*12*/*15*/*35*/*42*/*44* were relatively higher in ‘Jin-16’ than that in ‘Red Rose-2’ and ‘Xisen-8’. Accordingly, these 7 genes were positively correlated with each other, but negatively correlated with most of the highly expressed genes in colored potato tubers (Additional file 3: Fig. S3c).

To further validate the expression of *StKFB* genes in potato tubers, the qRT-PCR technique was used to detect the transcript levels of 9 randomly selected *StKFB* genes in different potato cultivars. Primer sequences of these genes were shown in Additional file 1: Table S9. And the primer specificity of each gene was presented by the melting curve (Additional file 3: Fig. S4). The expression of *StKFB03* in tubers of ‘Jin-16’ was set to 1 and the expression of other genes in different cultivars were compared with that (Fig. 8d). Generally, the expression trend of individual *StKFB* gene in different potato tubers shown in qRT-PCR was basically consistent with RNA-seq data (Additional file 3: Fig. S5). Among these selected genes, the expression levels of *StKFB16* and *StKFB31* were the lowest, and there was no significant difference among the three potato varieties. On the contrary, *StKFB39* had the highest expression level in three colored potato tubers, followed by *StKFB29*, *StKFB27*, *StKFB14* and *StKFB03*. Specifically, the expression levels of *StKFB03*, *StKFB27* and *StKFB39* were significantly higher in ‘Red Rose-2’ and ‘Xisen-8’ than that in ‘Jin-16”. While other genes, such as *StKFB15* and *StKFB44* witnessed decreased expression levels in ‘Red Rose-2’ and ‘Xisen-8’ in comparison with ‘Jin-16′. Additionally, the expression of *StKFB29* in ‘Xisen-8′ tubers was significantly lower than that in ‘Jin-16′ and ‘Red Rose-2′. These genes that were differentially expressed among ‘Jin-16′, ‘Red Rose-2′ and ‘Xisen-8′ ﻿are potentially involved in anthocyanin biosynthesis.

## Discussion

### The diversity and complexity of KFB structures make their functions diversified

Although both the F-box proteins and the Kelch containing proteins can bind to other proteins to mediate the substrates degradation via ubiquitylation pathway in all organisms, some studies have found that proteins that co-exist with the F-box domain and Kelch motifs were only observed in eukaryotes [41, 51, 52]. Compared to KFB in human and other animals, a large number of KFB members were identified in plants [12]. More than 103, 68 and 31 KFB members were identified in *Arabidopsis thaliana*, *Populus trichocarpa* and *Salvia miltiorrhiza*, respectively [1]. To date, multiple *KFB* genes have been isolated from chickpea, *Arabidopsis*, wheat and so on [14–16], but the potato *KFB* members have not been systematically identified and investigated. In this study, 44 *KFB* genes from potato (*Solanum tuberosum*) were identified and analyzed in phylogenetic relationship, extron-intro organization, motif composition, syntenic relationship and expression patterns. However, these 44 members may not represent all the *KFB* genes in the potato genome. The main reason is the lack of strictly conserved sequences in the F-box domains and Kelch motifs [3, 9], in which only a few amino acid residues are relatively invariant (Fig. 1, Additional file 1: Table S2 and S3). Therefore, it is possible that there exist other StKFB members that have not been detected.

By analyzing the protein sequences of F-box domains of StKFBs, we found that L at the 8th and 20th positions, P at the 9th position, I at the 16th position, and C or S at the 32nd position were highly conserved residues, which is consistent with the results of existing research [3]. Besides, D (aspartic acid), L, P, V (valine) at the 11th, 17th, 21st and 31st positions, respectively, were also conserved in F-box domains of StKFBs. However, due to the discontinuity of these relatively conserved amino acids, the sequence identity of F-box domain is low, which makes it difficult to identify KFB members.

Kelch motif is the secondary domain of KFB proteins [8], and characterized by 8 highly conserved amino acids: 4 hydrophobic amino acid residues, 2 glycine (G) and 2 aromatic amino acid residues (Y or W) (Additional file 1: Table S3). Multiple Kelch motifs would be folded to form a β-propeller with a pocket that coordinates ions required for enzyme activity and is the most likely site for KFB substrate binding [9]. The motif distribution of StKFB members were further analyzed. Based on annotation of the conserved motifs, Motif 1, 4, 10, 12 and 15 were predicted as parts of the F-box domains, while Motif 2, 3, 5 and 13 were Kelch domains (Additional file 1: Table S4). These different motifs belong to the same domain, showing the variability of this domain.

F-box domains and Kelch domains have been identified as essential components for degradation of regulatory proteins via UPS [12]. The F-box domain recognizes and binds with SKP1 to form the SCF E3 ubiquitin ligase complex; while Kelch domain is responsible for selectively interacting with target proteins [53]. Therefore, the variability of the Kelch domain is important for the recognition of different substrates, which has been demonstrated in both animals and plants. For example, α-Scruin, a Kelch repeat protein in Limulus spermatozoa, has been demonstrated to bind with F-actin and participate in actin stabilization and crosslinking. While β-scruin, having 67% sequence identity with α-Scruin, was located in the actin-free acrosomal vesicle and had different binding partners from α-scruin [9]. In *Arabidopsis*, AtKFB50 (At3g59940) and AtKFB^CHS^ (At1g23390) respectively recognized and bind to PAL and CHS, mediating their proteolysis [14, 29]. Besides, the number of Kelch repeats varies in different KFB family members, which may also be a vital factor that causes the difference in KFB functions [8]. In this study, most potato KFB members (30/44) contain 1–2 Kelch motifs, followed by those containing 3 Kelch motifs (8 members). StKFB members containing 4–6 Kelch motifs are the fewest, with only 6 members in total. Although it is known that β-propellers structure formed by multiple Kelch repeats can produce different contact sites and interact with different partners, the most key residues associated with substrate proteins remain unknown. Moreover, due to the low sequence similarity of the Kelch motifs, it is almost impossible to infer its function from the primary sequence of KFB. In addition, many of them have degenerated Kelch motifs, suggesting that they might be pseudogenes or their functions may be divergent [41]. Therefore, the binding substrates of these StKFB members and their functions need further experimental verification.

### The evolution of the StKFB family is relatively stable, and the duplicated genes may result in functional differentiation of StKFB members

Previous studies implied that KFB family originated before the branching of animals and plants, and may have undergone a rapid evolution in some land plants [12]. Sun et al. have found that one of the KFB subfamilies (G5) included large numbers of *KFB* genes in *Arabidopsis*, but had very few members in rice, pine and poplar, suggesting that a rapid gene birth of *KFBs* has occurred in *Arabidopsis* [41]. Also, a phylogenetic analysis of KFB proteins from *S. miltiorrhiza*, *Arabidopsis*, rice, human, mice and *C. reinhardtii* showed that 67 of 69 KFB members in Group I belong to *Arabidopsis* [1]. Similarly, in our results of KFB family evolutionary relationship among potato, *Arabidopsis*, rice and upland cotton, we found that 71 of the 76 members of Group I were *Arabidopsis* KFBs and only 5 KFBs were from other plants that we analyzed (Fig. 4).

One of the main driving forces of gene expansion is the occurrence of gene duplication events [12]. Multiple *KFB* genes in the G5 subfamily of *Arabidopsis* were found to be tandemly arrayed on the same chromosome, which probably led to the gene evolution [41]. Potato KFB family did not seem to undergo a rapid gene birth event like *Arabidopsis* KFBs. Forty-four *StKFB* genes were unevenly located on 12 potato chromosomes, including 2 pairs of tandem duplications (*StKFB15*/*StKFB16*, *StKFB40*/*StKFB41*) and 1 pair of segmental duplications (*StKFB16*/*StKFB29*) (Fig. 3). The Ka/Ks ratios of three pairs of duplicated *StKFB* genes were all less than 1, suggesting that the duplicated *StKFBs* might have undergone great selection constraint during evolution. Also, the Ka/Ks values of the orthologous pairs of *KFB* genes between potato and other plants were all less than 1, denoting that the corresponding homologous *KFBs* have not experienced positive selection (Additional file 1: Table S5). Besides, the syntenic analysis of *KFB* genes in different plants showed that the numbers of syntenic *KFB* pairs between potato and other dicots (*Arabidopsis*, pepper, tomato and upland cotton) were more than those between potato and the monocot (rice), indicating that potato *KFBs* had a closer syntenic relationship with those in dicots. Furthermore, multiple *KFB* orthologous pairs between potato and other two solanaceae plants (tomato and pepper) were arrayed on corresponding chromosomes and in corresponding orders, speculating that the syntenic relationship of potato *KFBs* was closer to the *KFBs* in tomato and pepper. The closely related gene members in the phylogenetic tree may have similar structure and function [33]. Therefore, phylogenetic analysis can be used as a preliminary method to study the potential function of the unknown *StKFBs*.

The existence of duplicated *KFBs* may result in redundancy of their function [41, 54]. For instance, two duplicated genes in *Arabidopsis*, *LKP1/ZTL/AtKFB98* and *LKP2/FKL2/AtKFB22*, were found to share redundant functions in controlling the circadian clock and flowering time [55]. Both *AtKFB29* and *AtKFB32* were involved in the anther development, indicating that they may participate in the similar biological processes and have redundant functions [41]. However, numerous studies have confirmed that gene evolution caused by gene duplication may also lead to the loss of original functions and the generation of new functions. Duplication events in the active and regulatory regions such as the CDS and the promoter region, may affect the function of family genes under evolution process [56, 57]. In tartary buckwheat, several duplicated *FtARFs* (like *FtARF7* and *FtARF13*) were highly expressed in different organs [50]. Similarly, many tandemly duplicated *AtKFB* members of G5 showed preferential expression in certain organs [41]. In this study, potato duplicated *KFBs* showed the different expression patterns in various potato organs and under diversified stresses (Fig. 7a and b). *StKFB41* was highly expressed in mannitol-treated potato plants, but *StKFB40* did not show obvious expression. Besides, *StKFB16* was mainly expressed in shoots and immature fruits, while its tandemly duplicated gene *StKFB15* was highly expressed in immature fruits and stolon. *StKFB29*, the segmentally duplicated gene of *StKFB15* was predominately expressed in stolon. It is possible that evolution leads to structural differences in proteins, such as the generation of degenerated Kelch motifs, and results in their divergent functions.

### Expression patterns and functional prediction of the *StKFB* genes

KFB proteins are widely involved in multitudinous biochemical and physiological processes in plants. The accelerated evolution of the KFB family may have contributed to more complex and varied protein-degradation mechanisms to improve plant adaptation to changing environments [12]. At present, the functions of some *KFB* genes have been deeply studied in *Arabidopsis*, rice and other model plants, while only a few *StKFBs* have been functionally investigated in potato. Therefore, the existing research results of *KFB* homologous genes in other species can be used as an important basis for the functional prediction of potato KFB family members. The functional annotations of *StKFB* members and their corresponding homologous genes in *Arabidopsis* are shown in Additional file 1: Table S10. According to the annotated information, we found that almost all KFBs may be involved in the degradation of specific proteins by UPS (Additional file 1: Table S10), thus playing an important role in different plant growth stages.

Primarily, the role of KFBs in different physiological processes of plant growth and development cannot be ignored. In this study, publicly available RNA-seq data was used to investigate the expression profiles of *StKFB* genes in several potato tissues and in potato plants with different treatments. The results showed that *StKFB10*, annotated as *S-haplotype-specific F-box* gene (*SFB*) (Additional file 1: Table S10), was specifically highly expressed in flowers (Fig. 7a), indicating that this gene may play an essential role in flower development. SFB specifically degrades non-self S-RNase through the formation of SCF^SFB^ complex with SCF, while its self S-RNase is not degraded. This inhibits the growth of self-pollen tubes by degrading ribosomal RNA (rRNA), thus presenting self-incompatibility in potato and other plants [58]. In addition, *StKFB08*, *StKFB13*, *StKFB20*, *StKFB22*, *StKFB28*, *StKFB33*, *StKFB35* and *StKFB36*, were also highly expressed in stamen or other flower tissues, indicating that they may also regulate potato flowering development. These studies provide evidence and direction for functional prediction of these *StKFB* genes, but the specific functional mechanism needs to be further studied.

*StKFB01* was a LOV blue light receptor gene (*StFKF1*) and was highly expressed in whole flowers, leaves and petioles in potato (Fig. 7a). It has been reported that StFKF1, StGI and StCDF1 would form a complex that mediates degradation of StCDF1 through ubiquitination pathway and ultimately induces the expression of *StCONSTANS* (*StCO*) [20]. *StCO* is essential for converting light and clock signals into flowering signals, thereby promoting flowering and inhibiting tuberization by regulating the expression of *StFT* and its homologous genes [59]. Therefore, *StKFB01* plays an important role in photoperiodic flowering and potato tuberization. Its orthologous genes *AtFKF1* (At1g68050) and *OsFKF1* (Os11g34460) also serve as photoreceptors that regulates flowering in *Arabidopsis* and rice [60, 61]. The similar function of these three KFB proteins may be attributed to the fact that they all contain a LOV domain belonging to the Per-Arnt-Sim (PAS) superfamily (Additional file 3: Fig. S6), which is a blue light sensing module [62]. Although StKFB27 belongs to the same group as these three KFBs, it is highly expressed in shoots and mature fruits (Fig. 7a), which may show different functions due to its lack of the LOV domain (Additional file 3: Fig. S6).

KFBs not only participate in the growth and development of organs and tissues, but also mediate plant defense signaling [12]. At present, the mechanism of F-box proteins response to stresses has been well investigated, while the regulation of KFBs in stress responses is rarely studied. It has been reported that multiple F-box genes, such as *ATPP2-B11* and *OsMSR9*, positively regulate salt tolerance in plants [63]. A nuclear KFB member in chickpea, named CarF-Box 1, was also found to have a positive response to salt stress [15]. In this study, *StKFB02/03/04/17/30/34/40* had up-regulated expression levels in salt-stressed potato plants compared with control group (Fig. 7b), implying that they may participate in salt stress response. For drought stress, the expression of *StKFB04/11/17/23/34/35/41* were up-regulated, while *StKFB06* was down-regulated in potato treated with mannitol. These genes may play positive or negative roles in potato drought tolerance. Similar results were found in other F-box proteins, such as TaFBA1 and GmFBX176, which are positive and negative regulators of drought tolerance in plants, respectively [64, 65]. Some *StKFBs* were also induced by heat, ABA, IAA and GA_3_, but the functional mechanism remains unclear. In addition, some *KFB* genes were identified to be involved in plant pathogen interaction as the “susceptibility” (S) genes, contributing to the successful infection of pathogens [12]. For example, KMD3/AtKFB39 (At2g44130), a KFB from *A. thaliana*, could be induced in roots by *Meloidogyne incognita* infection [27]. The expression of *BIG24.1* was induced by botrytis infection in grapevine [66]. However, in this study, we did not find any *StKFBs* that can be induced by *P. infestans* (Fig. 7b). Whether and in what way these *StKFB* are involved in potato response to *P. infestans* requires further investigation.

Additionally, some studies have clarified the involvement of KFBs in secondary metabolites production. OsFBK1 (Os01g47050) negatively regulated lignin synthesis by degrading Cinnamoyl-CoA Reductase (OsCCR), and thus affected the secondary cell wall thickenings of anther and root [67]. In *Arabidopsis*, Zhang et al. have elucidated that protein ubiquitination and degradation mediated by AtKFB01 (At1g15670), AtKFB20 (At1g80440), AtKFB39 (At2g44130) and AtKFB50 (At3g59940) regulated the proteolysis of PALs, thereby modulating phenylpropanoid metabolism [14]. In 2017, they also found that another KFB, named KFB^CHS^ (At1g23390), regulate the proteolysis of CHS and control flavonoid and anthocyanin biosynthesis in *Arabidopsis* [29]. However, there is limited understanding of the types of KFB interacting proteins involved in the ubiquitination pathway during secondary metabolism. Anthocyanin is one of the main secondary metabolites in the biosynthesis of plant flavonoid, which makes flowers, fruits and other organs show various colors under different pH conditions in plant vacuole [34]. Due to its outstanding free radical scavenging capacity, anthocyanin was demonstrated to have healthcare effects such as antioxidation, anti-aging, anti-tumor and immune activity regulation [68–70]. Purple-fleshed potato, accumulating large amounts of anthocyanin content, is regarded as high-value feedstock for food and industrial processing. To investigate which *StKFBs* might be involved in anthocyanin biosynthesis, transcriptomic analysis was performed on potato tubers with different colors, and its accuracy was verified by qRT-PCR on 9 randomly selected *StKFBs* genes. The results showed that most of the *StKFB* genes were differentially expressed in three colored potatoes. *StKFB15* and *StKFB29*, which were closely related with *AtKFB01* and *AtKFB20*, were down-regulated significantly in the purple-fleshed tubers (‘Xisen-8’) compared with the yellow-fleshed tubers (‘Jin-16’) (Fig. 8d). *StKFB07* and *StKFB23*, the homologous genes of *OsFBK1* and *AtKFB*^*CHS*^, respectively, also showed a downward expression trend in ‘Xisen-8’ tubers. ﻿Referring to the negative regulation of OsFBK1 and AtKFB^CHS^ in the synthesis of secondary metabolites, we hypothesized that StKFB07 and StKFB23 may also play a negative role in phenylpropanoid biosynthesis. Furthermore, other genes such as *StKFB11*/*18*/*30*/*38*/*42*/*44* were highly expressed in ‘Jin-16’ tubers and lowly expressed in the ‘Red Rose-2’ or ‘Xisen-8’. The different expression of the *StKFBs* suggested their potential roles in the modulation of anthocyanin biosynthesis. Notably, no expression of *StKFB43* was detected either in different potato tissues or potato plants under different treatments, indicating that this gene is likely to be a pseudogene. This result is consistent with the annotation of its homologous gene in *Arabidopsis*. These results provide a basis for predicting the functions of StKFB members, but their specific functions need to be verified by future experiments.

## Conclusion

In this study, a total of 44 *StKFB* genes were identified in potato genome. A series of analyses for these members, including gene structure, motif composition, phylogenetic relationship, duplication events, syntenic relationship and expression profiling were conducted. The *StKFBs* were classified into 5 groups according to the classification schemes of other plant species. Two pairs and one pair of genes were predicted to be tandemly duplicated and segmentally duplicated genes, respectively. The syntenic analysis showed that the *KFBs* in potato were closely related to the *KFBs* in tomato and pepper. Expression profiles of *StKFBs* manifested their distinct expression patterns in various tissues and in response to diversified stresses, and their potential roles in anthocyanin biosynthesis. These findings are helpful to screen candidate *StKFBs* for further functional characterization, and provide the basis for genetic improvement of potato agronomic traits.

## Materials and methods

### Identification of KFB family members in potato

The profile HMMs (Hidden Markov Models) of F-box domains and Kelch domains downloaded from Pfam database [36] were used to search the StKFB members from the annotated protein sequences of potato (DM v4.03/v4.04) [38] using Hmmsearch program in HMMER software package v3.0 [37] (http://hmmer.org/) with default parameters. Potato protein sequences were acquired from Spud DB Potato Genomics Resources (http://spuddb.uga.edu/). Furthermore, *A. thaliana* KFB protein sequences (TAIR10), downloaded from TAIR database (https://www.arabidopsis.org/Blast/index.jsp), were used as queries to blast against the potato protein sequences with E-value ≤1e-5. These putative StKFB members were analyzed in PfamScan database [39] (https://www.ebi.ac.uk/Tools/pfa/pfamscan/) to remove the KFBs lacking the conserved domains. The repetitive sequences were also eliminated after multiple protein sequence alignment by MUSCLE algorithm [71] as implemented in MEGA X software [72]. The chromosome location, CDS and genomic length of the predicted *StKFB* genes were obtained from Spud DB Potato Genomics Resources. The number of Kelch repeat motifs included in each StKFB protein was calculated using PfamScan website (https://www.ebi.ac.uk/Tools/pfa/pfamscan/). Multi-sequence alignment and secondary structures prediction of F-box domains were conducted by DNAMAN X software v10.0.2.128 and the online tool provided by NovoPro Bioscience Inc. (https://www.novopro.cn/tools/secondary-structure-prediction.html), respectively. The tertiary structures of Kelch motifs with different numbers were predicted by SWISS-MODEL website [73] (https://swissmodel.expasy.org/interactive).

### Prediction of physicochemical properties and subcellular localization of StKFB members

The numbers of amino acids, theoretical molecular weights (MW), isoelectric points (pI) and grand average of hydropathicity (GRAVY) of these identified StKFB proteins were computed using ProtParam software provided by ExPasy website [74] (https://web.expasy.org/protparam/). Subcellular localization of StKFB family members was predicted by Plant-mPLoc website [75] (http://www.csbio.sjtu.edu.cn/bioinf/plant-multi/) and ProtComp 9.0 [76] (http://www.softberry.com/berry.phtml?topic=protcomppl&group=programs&subgroup=proloc).

### Chromosomal localization and gene duplication analysis of *StKFBs*

All *StKFB* genes were mapped on the potato chromosomes using Circos software [40] (http://circos.ca/software/download/) based on the physical position information obtained from the Spud DB Potato Genomics Resources (http://spuddb.uga.edu/). Gene duplication events of *StKFBs* were analyzed by Multiple collinear scanning toolkits (MCScanX) [77] (https://github.com/wyp1125/MCScanx) with default parameters. The synonymous substitution (Ks) and non-synonymous substitution (Ka) was calculated by TBtools software [78] (https://github.com/CJ-Chen/TBtools/releases). The divergence time of duplicated *StKFB* genes was estimated according to the method of Shen and Yuan [43].

### Analyses of conserved motifs and exon-intron organization

Conserved motifs of the putative StKFB proteins were identified by Multiple Em for Motif Elicitation (MEME) software v5.3.0 (http://meme-suite.org/meme-software/5.3.0/meme-5.3.0.tar.gz) [46]. The parameters were set as follows: ﻿the number of motifs searched was set to 20 and the range of the motif length was set to 6–200 residues. All motifs were further annotated with InterProScan (http://www.ebi.ac.uk/interpro/) [47, 48].

The CDS file and genomic sequences file of 44 *StKFB* genes were downloaded from Spud DB Potato Genomics Resources (http://spuddb.uga.edu/). The exon and intron distribution of *StKFBs* was depicted by comparing the CDS of *StKFBs* with their corresponding genomic DNA sequences using Gene Structure Display Server (GSDS 2.0) [79] (http://gsds.gao-lab.org/).

### Phylogenetic analysis and classification of KFB family members

The protein files of potato (DM v4.03/v4.04) [38], *Arabidopsis* (TAIR10), rice (*Oryza sativa* v7.0) and upland cotton (*Gossypium hirsutum* v1.1) were downloaded from Spud DB Potato Genomics Resources (http://spuddb.uga.edu/), TAIR database (https://www.arabidopsis.org/download/index-auto.jsp?dir=%2Fdownload_files%2FProteins%2FTAIR10_protein_lists), Rice Genome Annotation Project database (http://rice.plantbiology.msu.edu/), and Cotton Research Institute database (https://mascotton.njau.edu.cn/info/1054/1118.htm), respectively. Then the identification method of potato KFBs was used to search for KFB members of other species. A total of 284 KFB proteins, including 44 StKFBs, 115 AtKFBs, 39 OsKFBs and 86 GhKFBs, were considered for construction of an inter-species phylogenetic tree. Multiple sequence alignment of these KFB proteins was performed using MUSCLE algorithm [71]. The maximum-likelihood (ML) method [80] of IQ-TREE software v2.1.4 [44, 45] (http://www.iqtree.org/) was applied to construct the phylogenetic tree with 1000 bootstrap replicates. The model VT + F + R7 was automatically evaluated as the best-fit model through ModelFinder [81] analysis. Potato KFB members were categorized into different groups based on the KFB classification schemes of *Arabidopsis*, rice and upland cotton.

Moreover, a phylogenetic tree of KFB proteins from potato was also constructed and analyzed. Multiple sequence alignment of the 44 potato KFB proteins was carried out using MUSCLE algorithm [71], and the phylogenetic tree was constructed by the unrooted neighbor-joining [82] method with 1000 bootstrap replicates using IQ-TREE software.

### Synteny analysis of *KFB* genes in potato and other plant species

Protein sequences of potato, *Arabidopsis*, rice and upland cotton were obtained using the method described above. Protein sequence files of tomato (*Solanum lycopersicum*) (ITAG4.0) and pepper (*Capsicum annuum*) (ASM51225v2) were obtained from Phytozome v13 (https://phytozome-next.jgi.doe.gov/) and EnsemblPlants (http://ftp.ensemblgenomes.org/pub/plants/release-52/fasta/capsicum_annuum/), respectively. The Makeblastdb program [83] (https://www.ncbi.nlm.nih.gov/books/NBK569841/) was applied to build local databases of protein sequences from these six plant species, and the potato protein sequences were then compared pairwise with those of five other species using Blastp [84]. The Syntenic relationship was analyzed by MCScanX software [77] (https://github.com/wyp1125/MCScanx).

### Plant materials and anthocyanin determination

In this study, three tetraploid cultivars (‘Jin-16’ with yellow skin and yellow flesh, ‘Red Rose-2’ with red skin and red flesh, and ‘Xisen-8’ with purple skin and purple flesh) were used as plant materials. The tissue culture plantlets of ‘Jin-16’ were preserved in the College of Agriculture, Shanxi Agricultural University. The virus-free seedlings of ‘Red Rose-2’ and ‘Xisen-8’ were kindly provided by Leling Xisen Potato Industry Co. Ltd. (Leling, Shandong, China). They were cultured in MS medium at 22 ± 1 °C under 16 h light/8 h dark regime. The 1-month-old tissue culture plantlets were transferred into pots with soil and grown in the greenhouse at 22 ± 1 °C under 16 h light/8 h dark regime. After 3 months, fresh tubers were harvested for anthocyanin determination and RNA extraction.

Three potato tubers with similar sizes were selected from each potato variety and blended separately. Anthocyanin was extracted according to the method used by Wang et al. [85]. The potato flesh from each tuber was ground into powder and then exposed to HCl-methanol solution (1:99 by volume) at 4 °C for 6–8 h under darkness until the tissues were completely decolorized. After centrifuging at 12000 rpm for 10 min, the absorbance values of supernatants were determined at 530 nm using a UV-2450 spectrophotometer (Shimadzu, Kyoto, Japan). Each sample had three replicates to ensure the results reliable.

### Total RNA extraction, library construction and transcriptome sequencing

Total RNA was isolated from the collected samples using the Quick RNA Isolation Kit (Huayueyang, Beijing, China). Electrophoresis was then performed with 1% agarose gel to monitor the presence of RNA degradation and DNA contamination. Nanodrop 1000 spectrophotometer (Thermo Scientific) was utilized to measure the purity and concentration of RNA samples. After integrity testing by Agilent 2100 BioAnalyzer (Agilent Technologies), the total RNA samples were used for the construction of cDNA libraries and validation of deep sequencing results. ﻿.

Total RNA with ribosomal RNA removal was trimmed into shorter fragments of 250 ~ 300 bp using fragmentation buffer. The first strand of cDNA was synthesized using fragment RNA as template and random oligonucleotide as primer. The second strand of cDNA was subsequently synthesized using dNTPs as raw materials in the DNA polymerase I system. After end-repair, 3′ end adenylation and ligation of the Illumina sequencing adapters, the double-stranded cDNA fragments were purified and amplified by PCR to construct the final libraries. Three biological replicates were set for each potato cultivar. Therefore, 9 libraries were constructed, containing Jin-16_1, Jin-16_2, Jin-16_3, Red Rose-2_1, Red Rose-2_2, Red Rose-2_3, Xisen-8_1, Xisen-8_2 and Xisen-8_3. After quantitative and qualitative determination of all libraries, RNA sequencing was carried out on an Illumina novaseq 6000 platform provided by Novogene Bioinformatics Technology Co. Ltd. (Beijing, China), and ﻿150 bp paired-end reads were generated. The obtained raw reads were processed by getting rid of the low-quality reads, the reads with sequencing adapters and poly-N sequences. The clean reads were acquired and aligned to a potato reference genome (DM v4.03/v4.04) using HISAT2 software [86]. The mapped reads were spliced and assembled into transcripts using Stringtie software [87] and Cuffmerge software [88]. The obtained transcripts were annotated by Cuffcompare 2.2.1 (http://cole-trapnell-lab.github.io/cufflinks/manual/). The FPKM values (fragments per kilobase of transcript sequence per millions base pairs sequenced) of genes were calculated using Stringtie software. The dataset was deposited in the NCBI Sequence Read Archive under the Bioproject accession PRJNA729884 (available from https://dataview.ncbi.nlm.nih.gov/object/PRJNA729884?reviewer=ntlkjmravag9c9ousg57ps9k86).

### RNA-seq analysis of *StKFB* genes

The publicly available dataset for FPKM values of all the representative transcripts across 40DM and 16 RH libraries: DM_RH_RNA-Seq_FPKM_expression_matrix_for_DM_v4.03_13dec2013_desc.xlsx (http://spuddb.uga.edu/pgsc_download.shtml) [38] was used to examine expression patterns of 44 *StKFBs* in 13 potato tissues, including roots, shoots, leaves, petioles, stolon, tubers, stamens, sepals, carpels, petals, whole flowers, immature and mature fruits. This dataset was also applied to analyze the expression levels of *StKFBs* in whole potato plants with different treatments. For abiotic stresses, the plants were exposed to stresses for 24 h including salinity (150 mM NaCl), drought (260 μM mannitol), heat (35 °C), as well as hormone treatments like ABA (50 μM), IAA (10 μM) and GA_3_ (50 μM). For biotic stress, the sequencing data was obtained from mixed samples of potatoes infected with *Phytophthora infestans* for 24, 36 and 72 h. In addition, the transcriptome sequencing data obtained in our lab was used to perform the expression analysis of *StKFBs* in tubers from cultivars containing various levels of anthocyanin content (cultivar ‘Jin-16’, ‘Red Rose-2’ and ‘Xisen-8’). Each variety had three biological replicates. The lg (FPKM+ 1) values were normalized using Scale function and displayed in heatmaps using tidyverse v. 1.3.1 (https://search.r-project.org/CRAN/refmans/tidyverse/html/tidyverse-package.html), ggplot2 v. 3.3.5 (https://cran.r-project.org/web/packages/ggplot2/index.html) and pheatmap v. 1.0.12 (https://CRAN.R-project.org/package=pheatmap) packages in RStudio. Furthermore, the correlation between the expression patterns of *StKFB* genes was analyzed based on the Pearson correlation coefficient [89] and graphically presented using corrplot package v. 0.92 (https://cran.r-project.org/web/packages/corrplot/).

### Expression analysis of selected *StKFBs* by qRT-PCR

Quantitative real-time polymerase chain reaction (qRT-PCR) was carried out with the TB Green™ Premix Ex Taq™ (Tli RNase H Plus) (Takara, Dalian, China) on CFX96 PCR System (Bio-Rad, USA). Primers of these *StKFB* genes were designed by Primer-Blast [90] in NCBI website (https://www.ncbi.nlm.nih.gov/tools/primer-blast/), and their specificity was tested by dissociation curve analysis. The 10 μl reaction volume samples, containing 5 μL TB Green, 1 μL diluted cDNA sample, 0.4 μL 10 μM solution of each primer and 3.2 μL ddH_2_O, were used for PCR with the following cycling program: 95 °C for 3 min, followed by 40 cycles of 95 °C for 10 s, 60 °C for 30 s, and 72 °C for 20 s. Dissolution curves were obtained by heating the amplicon from 60 °C (5 s) to 95 °C (50 s). The relative expression of selected *StKFB* genes was calibrated against the reference gene *EF1α* using the method of 2^-∆∆Ct^ [33]. ﻿Three tubers selected from each potato cultivar were mixed into one sample, and each sample had three replicates. The relative expression amounts of genes were displayed in boxplots using tidyverse v. 1.3.1 (https://search.r-project.org/CRAN/refmans/tidyverse/html/tidyverse-package.html), cowplot v. 1.1.1 (https://CRAN.R-project.org/package=cowplot), ggplot2 v. 3.3.5 (https://cran.r-project.org/web/packages/ggplot2/index.html) and ggsci v. 2.9 (https://CRAN.R-project.org/package=ggsci) packages in RStudio. Results were presented as means ± SD. The one-way ANOVA ﻿of variance was used to conduct the statistical analyses of qRT-PCR results by SPSS software v26 [91]. ﻿The Duncan’s Multiple Range Test (DMRT) post hoc test was used to measure specific differences between pairs of means ﻿at 0.05 level of significance. The Bonferroni algorithm provided by SPSS software v26 was used for *p*-values correction [92].

## Supplementary Information

**Additional file 1 Table S1.** The information of profile HMMs of F-box and Kelch domains in Pfam database. **Table S2.** The sequences and positions information of F-box domains in 44 StKFB members. **Table S3.** The sequences and positions information of Kelch motifs in 44 StKFB members. **Table S4.** The 20 conserved motifs in StKFB proteins identified by MEME software and motifs annotation analysis by InterProScan. **Table S5.** The orthologous *KFB* genes identified by comparison between potato and other plants. **Table S6.** Expression profiles of 44 *StKFB* genes in different potato tissues, in potato plants with different treatments and in tubers with different colors. The FPKM values of 44 *StKFB* genes in different potato tissues and in potato plants with different treatments were extracted from RNA-Seq Gene Expression Data: DM_RH_RNA-Seq_FPKM_expression_matrix_for_DM_v4.03_13dec2013_desc.xlsx, the excel file of FPKM values of all the representative transcripts across 40 DM and 16 RH libraries (http://spuddb.uga.edu/pgsc_download.shtml); the FPKM values in tubers with different colors was extracted from RNA-seq data in our lab deposited in the NCBI Sequence Read Archive under the Bioproject accession PRJNA729884. **Table S7.** Quality of transcriptome sequencing of potato tuber with three colors. ﻿Raw reads: Number of reads in raw data; Clean reads: Number of reads filtered from raw data; Raw bases: The number of bases in the raw data; Clean bases: The number of bases filtered from the raw data; Error rate: Error rate of data sequencing; Q20: Percentage of bases with a Phred value greater than 20; Q30: ﻿Percentage of bases with a Phred value greater than 30; GC content: The percentage of G and C in clean reads. **Table S8.** Sequence alignment results of reads mapped to the reference genome (DM v4.03/v4.04). Total reads: the number of clean reads used for mapping analysis; Total mapped: the number of reads that could be mapped to the reference genome; Multiple mapped: the number of reads mapped to multiple locations in the reference genome; Uniquely mapped: the number of reads mapped to single location in the reference genome; Read-1 and Read-2: the number of reads mapped to the reference genome in Read 1 and Read 2, respectively; Reads mapped to ‘+’ and Reads mapped to ‘-’: the number of reads mapped to the positive and negative strands of the reference genome, respectively; Non-splice reads: the number of reads with the entire segment mapped to exons; Splice reads: the number of segmented reads mapped on two different exons; Reads mapped in proper pairs:the number of reads paired mapped to the reference genome; Proper-paired reads map to different chrom: the number of paired reads mapped to different chromosomes in the reference genome. **Table S9.** All primers used in qRT-PCR. **Table S10.** The annotation of 44 *StKFBs* and their corresponding orthologous genes in *Arabidopsis thaliana*. The potato StKFB protein sequences were aligned with those of *Arabidopsis thaliana* using Blastp.

**Additional file 2.** CDS and protein sequences of 44 StKFBs.

**Additional file 3 Fig. S1.** Gene duplication analysis of potato genome. The local database of potato protein sequences was established by Makeblastdb program. And pairwise comparisons were made between potato protein sequences by Blastp with E-value ≤1e-10. The gene duplication analysis result was obtained by duplicate_gene_classifier program provided in MCScanX software. Singleton: single copy genes; Proximal: adjacent but discontinuous repetitive genes on the same chromosome; Tandem: tandem duplications; WGD or segmental: whole genome duplications or segmental duplications; Dispersed: dispersed genes. **Fig. S2.** Sequence logos of conserved motifs in StKFB proteins. The 20 conserved motifs of the putative StKFB proteins were identified by MEME software v5.3.0. **Fig. S3.** The correlation analysis between the expression patterns of *StKFBs* in diverse potato tissues (**a**), in potato plants with different treatments (**b**) and in three colored potato tubers (**c**). ﻿The correlation between the expression levels (FPKM values) of *StKFBs* was analyzed by Pearson’s correlation coefficient and plotted using the corrplot package v. 0.92 (https://cran.r-project.org/web/packages/corrplot/). **Fig. S4.** Dissociation curves of primers for qRT-PCR. Dissolution curves were obtained by heating the amplicon from 60 °C (5 s) to 95 °C (50 s) on CFX96 PCR System (Bio-Rad, USA). **Fig. S5.** Comparison of the expression levels of the 9 selected *StKFB* genes determined by qRT-PCR and transcriptome sequencing. The boxplots were plotted using tidyverse v. 1.3.1, cowplot v. 1.1.1, ggplot2 v. 3.3.5 and ggsci v. 2.9 packages in RStudio. Values are means ± SD of three replicates in each experiment. Bars with different lowercase letters represent significant difference at *p* < 0.05. **Fig. S6.** Conserved domain analysis of StKFB01, AtFKF1, OsFKF1 and StKFB27. The conserved domain analysis was conducted by Conserved Domain Search tool (https://www.ncbi.nlm.nih.gov/Structure/cdd/wrpsb.cgi) in NCBI.

## Data Availability

The datasets generated and/or analyzed for this work were deposited in the NCBI Sequence Read Archive under the Bioproject accession PRJNA729884, available from https://dataview.ncbi.nlm.nih.gov/object/PRJNA729884?reviewer=ntlkjmravag9c9ousg57ps9k86. Other datasets used in this study are included in this published article and its supplementary information files.

## Abbreviations

AFR: Attenuated Far-red Response
ABA: Abscisic acid
CCR: Cinnamoyl-CoA reductase
CDS: Coding sequence
CDF: Cycling Dof Factor
CFK1: COP9 interacting F-box Kelch 1
CHS: Chalcone synthase
CO: CONSTANS
CTG10: Cold Temperature Germinating 10
DMRT: Duncan’s Multiple Range Test
E3: Ubiquitin-ligation enzymes
FKF1: Flavin-binding Kelch repeat F-box 1
GA_3_: Gibberellin A3
GRAVY: Grand average of hydropathicity
﻿GSDS: Gene Structure Display Server
HMM: Hidden Markov Model
IAA: Indole-3-acetic acid
KFB: Kelch repeat F-box
Ka: The number of non-synonymous substitutions per non-synonymous site
Ks: The number of nonsynonymous substitutions per nonsynonymous site
LKP2: Light, oxygen or voltage Kelch protein 2
LOV: Light, Oxygen or Voltage
MeJA: Methyl jasmonate
MEME: Multiple Em for Motif Elicitation
ML: Maximum-likelihood
MW: Molecular weights
Mya: Million years ago
NJ: Neighbor- joining
PAL: Phenylalanine ammonia-lyase
PAS: Per-Arnt-Sim
PGSC: Potato Genome Sequencing Consortium
pI: Isoelectric point
PIF1: Phytochrome Interacting Factor 1
qRT-PCR: quantitative real-time polymerase chain reaction
rRNA: Ribosomal RNA
SCF: Skp1-Cullin-F-box
SFB: S-haplotype-specific F-box
SKP1: S-phase Kinase-associated Protein 1
UPS: Ubiquitination-26 s proteasome system
ZTL: ZEITLUPE
