# Supplementary material for: Genome-wide identification, evolutionary and functional analyses of KFB family members in potato
Source: BMC Plant Biol. 2022 May 2;22:226. doi: 10.1186/s12870-022-03611-y (PMC9063267; doi:10.1186/s12870-022-03611-y)
Supplement: Supplementary file 2 — Additional file 2. CDS and protein sequences of 44 StKFBs. [file 12870_2022_3611_MOESM2_ESM.docx]

**Additional file 2 CDS and protein sequences of 44 StKFBs**

**CDS of 44 *StKFBs***

>*StKFB01*

ATGGAAGAAGAAGAAGAGAACATGAGAAGAGAAAAGAGATTGAAATGCATAAACATGGAGGAAGATGAAGATGAAGATGAAGAAGAAGGAGAAATTCTCGATGACGACGATGACGATGATGAATATGAAGAAGAAATTGAAAATATTGAAGTTCCGTCACAACCAGTTGGATTTTTCTATCCTTCAACGACGCCGTCTTCAATCGTCGTCTCCGATGCATTAGATCCTGATCTTCCGGTTATATATGTGAATTCTGCGTTTGAAAGCTCTACTGGTTATCGCGCTGACGAAGTCCTCGGTCGTAACTGTCGGTTTTTGCAATTTAGAGATCCACGTGCTCAAAGGCGGCATCCTTTGGTGGATCCTGTTGTTGTTTCTGAGATAAGAAGATGTCTTGAAGAAGGTGTTGATTTCCAAGGGGAGCTTCTCAACTTTAAAAAAGATGGTACACCTGTGGTGAACAGGCTAAGGCTAGCACCCATACATAGTGATGATGGCACAGTGACCCATATTATAGGGATTCAAATGTTTTCTGAAACAAAAATTGACCTGAATACTGTGTCATATCCCGTTTTCAAAGAAACTTGCCAGCCTCACTGTGACGAGTCCAGTGAGTACTCCATTAAAAGTGGTAATTTATTGCACCGTGAAATGTGCGGTATTCTTCAGCTCTCTGATGAAGTTCTAGCTCACAACATTTTATCTCGATTGACTCCAAGGGATGTTGCATCCATTGGTTCTGTTTGTAGAAGGATACGCCAATTGACGAAAAATGAGCATGTGAGAAAAATGGTCTGTCAAAATGCATGGGGAGCTGATGTCACAGGTGTGCTGGAACACATGACAAAGAAGTTAGCTTGGGGGCGTCTGGCTAGGGAGC

TAACCACTCTTGAAGCAGTTTGTTGGAAGAAACTGACAGTAAGAGGTGCTGTAGAGCCTTCACGTTGCAACTTCAGTGCATGTGCTGCAGGGAACAGGCTAGTGTTATTTGGAGGGGAAGGTGTTAATATGCAGCCAATGGATGACACATTTGTTCTCAATCTTGATGCTGCTAATCCAGAATGGCGACGAGTGAGTGTCAAATCATCTCCACCAGGACGTTGGGGCCATACTCTCTCATGTCTTAATGGTTCCTGGTTGGTGGTGTTTGGTGGATGTGGGAGGGAGGGATTGCTTAATGACGTGTTTGTTCTTGATTTAGATGCTAAACAGCCTACATGGAAAGAAGTATCTGGTGGAACTCCCCCCCTTCCTAGGTCTTGGCACAGCTCTTGCACGATGGAAGGCTCAAAGTTAGTTGTTTCAGGTGGATGCACAGATGCGGGTGTACTTCTCAGTGACACATATTTGTTGGATCTGGCCAATGACAAACCTACCTGGAGAGAAATTCCAACTACATGGGCTCCTCCATCTAGATTGGGACACTCACTCTCAGCTTATGGGAAGACAAAAATTCTAATGTTTGGTGGCCTTGCCAAAAGCGGTCACTTGCGTTTAAGATCAGGTGAATCATACACTATTGATTTGGAGGATGAAAGGCCACAATGGAGGCAACTTGAGTGTGGTGCGTTCACAGGAGTAGGAAGTCAAAATGCTGTGGTTCCTCCTCCTAGACTTGATCACGTTGCTGTAACCATGCCTTGTGGCCGTATCATCATTTTCGGAGGTTCAATTGCCGGACTGCACTCTCCTTCACAACTCTTTCTACTAGATCCTTCAGAGGAAAAACCGTTATGGAGGACTCTCAATGTACCTGGGCAACCTCCAAAGTTTGCTTGGGGTCATAGCACATGCGTGGTTGGAGGAACAAGGGTCTTGGTCCTAGGAGGCCATACCGGGGAGGACTGGGTTCTGAATGAATTGTATGAATTGTGCTTAGCGAGCAAGCAAGATTCTGATGCATGA

>*StKFB02*

ATGGACCAAACAATTGAGAGGTCTCCAAATGCACACAGGGGTTTTAGAGTTCAAGCTCCACTGGTTGATTCCGTATCATGCTATTGCAAGGTTGACTCAGGGTTTAAGACAGTTGTAGGGGCAAGAAAGTTTGTCGCAGGATCAAAAATTTGCATCCAGCCGGACATTAATCCTCACGCACACAAGACTAAAAACTCCCGTAGGGAGAGGTCAAGAGTGCAGTCACCTCTTCTGCCTGGTCTACCAGATGATCTTGCAATTGCTTGTCTAATACGTGTTCCTCGTGTTGAACACAACAAGCTCCGTTTAGTCTGCAAAAGGTGGTATCGGCTTCTTGCTGGAAATTTCTTTTACTCTCTCAGGAAGAGTCTTGGAATGGCGGAGGAGTGGGTATACGTGATAAAGAGAGATCGTGACGGAAGGATTTCATGGCATGCATTTGATCCAACATACCAACTATGGCAGCCACTTCCACCTCTTCCTGGGGAATATAGTGAAGCCCTTGGATTTGGTTGTGCTGTTCTTAGTGGTTGCCATCTCTACTTGTTTGGAGGAAAAGATCCAATCAAGGGGTCTATGCGACGGGTAATCTTTTATAGTGCTCGTACAAATAAATGGCACAGGGCACCAGACATGCTTCGTAAACGTCATTTCTTTGGTTCTTGTGTAATCAACAATTGTCTTTATGTTGCTGGTGGAGAATGTGAAGGAATCCAAAGGACTCTCCGCTCAGCTGAGGTTTATGACCCCAATAGGAACCGCTGGACCTTTATTACTGATATGAGCACAGCTATGGTGCCCTTTATTGGGGTTATTTATGACGGGAAATGGTTTTTGAAAGGGCTGGGATCCCACAGAGAAGTTCTAAGTGAAGCCTATAACCCGGAAACCAATGGATGGAGCCCTGTCACTGACGGGATGGTTGCTGGTTGGCGCAACCCAAGCATTTCCATGAACGGTTGCCTTTATGCTTTGGACTGTCGTGATGGGTGTAAACTTAGAGTATATGATGGAGCCTCAGATTCGTGGAATAGATTCATTGACAGCAAACTCCATCTTGGAAGTTCTCGTGCTTTAGAGGCTGCAGCTCTGGTTCCTCTTAATGGTAAACTCTGTATAATTCGTAACAACATGAGCATTAGTATGGTTGATGTATCAAGCCCTGATAAACAAGTGGAAACTAACCCACATCTTTGGGAGAACATTGCTGGTAAAGGTCACTTCAGAACTCTATTTACTAATTTATGGTCAAGCATCGCAGGACGAGGAGGGTTGAAGAGCCATATTGTGCATTGTCAGGTTCTACAAGCCTGA

>*StKFB03*

ATGGACCAAACAATTGAAAGGTCTTCAAATGCACATAGGGGTTTTCGAGTTCAGCCTCCACTGGTTGATTCTGTATCATGCTATTGCAATGTTGATTCGGGCTTAAAGACAGTTGCTGGGGCAAGAAAATTTGTCCCAGGTTCAAAACTTTGTATCCAGTCAGACATCAGTTCTCATGCACACAAAAGTAAAAACTCTCGAAGGGAGAGGTCAAGAGTGCAGCCACCTCTTCTGCCTAGCCTACCTGATGATCTTGCAATTGCTTGTCTAGTACGTGTTCCTCGTGTTGAACTTAGCAAGCTCCGTCTAGTTTGCAAAAGATGGTATAGGCTTCTTGCTGGTAACTTCTTTTACTCTCAAAGGAAGAGTCTTGGAATGGCTGAAGAGTGGGTATATGTGGTTAAAAGAGATCGTGATGGACGGATTTCATGGCATGCATTTGACCCAACTTACCAACTTTGGCAGCCACTTCCACCTGTTCCAGGGGATTATAGTGAAGCCCTTGGATTTGGTTGTGCTGTTCTTAGTGGTTGCCATCTTTATTTGTTTGGAGGGAAAGATCCAATCAAGGGGTCTATGCGAAGGGTAATCTTTTACAATGCTCGAACAAATAGATGGCACAGGGCACCAGACATGCTCCGCAAACGCCATTTCTTTGGCTCTTGTGTAATTAATAATTGTCTTTATGTTGCTGGTGGAGAATGTGAAGGAATACAGAGGACTCTTCGTTCAGCTGAAGTTTATGACCCAAACAGGAAGCGCTGGAGTTTTATTGCTGATATGAGCACAGCTATGGTGCCTTTTATTGGGGTAGTGTATGATGGGAAGTGGTTTATAAAAGGTTTGGGATCCCACAGAGAAGTTCTCAGTGAAGCTTATAACCCTGATACAAATGCATGGAGCCCAGTTAACAACAGGATGGTTGCTGGTTGGCGCAACCCAAGCATCTCCATGAATGGTCGTCTATATGCTTTGGATTGTCGTGATGGGTGCAAACTTAGAGTATATGATGAATCTACTCAGTCATGGATCAGATTTATTGATAGCAAGCTCCACCTTGGAAGTTCTCGTGCTTTGGAAGCTGCTGCTCTGGTTCCCCTTAATGGTAAACTTTGTATAATTCGTAACAACATGAGCATCAGCATCATTGATGTGTTGAGTCCTGATAAGCGTGTGGAAACTAACCCTCATCTTTGGGAAAACATTGCTGGTAAAGGTCACTTCAGAACTATGTTCACAAATTTATGGTCAAGTATTGCAGGACGAGCAGGTTTGAAGAGTCATATTGTGCACTGTCAGGTTTTACAAGTTTGA

>*StKFB04*

ATGGCGCATGAATCAGAATCAGTGTCACTTCTTCCTCCAGAAGTAATATCTGAAATACTGGTAAGGCTACCCGTGAAATCCTTGTTGATGATGAGGTGCGTTTCTAAATCTTGGCTTTCTCTAATCTCAACTCGCCAATTCATCAAAACCCATTTAGAATTTTCAACCAACAAACAAGATTTTGCTCACGACATCCTTCTTCTTAGTAGCTCTTCTTATGAGCATACCCTAAGATTTTACACTTGCTCTCTTTATGCAATTATGTATCAAGAATCTCCCCATGTACCTGATGACCTTGACTTTCCTTGCAAAGACCCACTCGTTGAATATAACTTTGTGGGTTCATGTGACGGGTTGCTCTGTATTTCTGGTGGTGCTCGTGATCTATTTCTATGGAATCCGTCTATAAGAAAGTCGAAGAAACTGCCAATGTCAGGAAGTAATGTGCATTGTAGTTCGTATTTGGCTTATGGTGTTGGTTATAATGAGTGTCAAGATGATTACAAAGTTGTAGAAGTTATGGGAAGTTCGCATAGTGAGTATGGGTTTCAGAATGAGTTTAGGGTTTATAGTCTAAGAACTAATTCTTGGAAAATGATTCAAGAGTACCCCGGTGTTATATTTTGCAATGATCCTGCTAAATTTGTGAATGGAAGACTTCATTGGATTGCTACTCGTGTTAGTGATAAGAATGACTCATGGTTTATCTCTTCATTAAACCTCGTCGATGAGACATATGAAAATGTAGGATTGCCTGATCTTGTTTATGGCAATTTTGATTGGGAATTAGGGATTTTAGGTGGTAATCTATGTGTATTTTGTGACTATTATAAGGTTCAGATGGATGTGTGGGTAATGAAGGCATATGGACTTGTGGAGTCGTGGACTAAGGTGGCTTCAATCCCTTATTTTAGAGCTATTGAGCACTCGCCTTTCCCGGTTTTTATCTCCCATAATGATGAAATTTTGCTGCAACATGGTTCAAGTTTATTGATATATAATTCAACAGACAACACTTTCAAGCATCCTCAGGTTCAGATTCATCATGGTTATGAAATTCAATTTAGTTTGTACACGAAAAGTCTTGTTTCACCACATTTTGTAGAGGATTGA

>StKFB05

ATGAGCAAAGGTGAAATTCTACCTCAGGATATCATAATTGACATACTATCTCGTCTTCCTGCAAAATCCATCGGCCAATACAGGTGCGTATCAAATCAATGGTACAATTTTCTCTCACACCCACAATTCATCAAATTTCACTTCACTCTCCATGCTCATAAACAAGAAACTAAACTCATTTTCATCTCTGATTCTGACGATCTTCACACTATCACCTTTAATCGTAACCCCCAAAATATAATACTCGATGCCATTTCAACGAACCTTAATTTTCAGAACAACTGGTTAAGTATTGCATGCTCATGTAATGGGCTGGTGTTGGTGGAAAATCAGGAACATATTATGTACTTAATTAACCCCACAACCTTAGATTACCATAGAATTCCAGTTTTTCATTTGGGTCTTCCTCAGCAAAGTAGCTACAGAGAGTATGGTTTTGGGTATGATTTTGCTAGTGATGATTATAAGGTGGTTAATCTTTCTCGATATCGAAAAGGGAATATTGACACTACTTTTGTTGATGTCTATTCGGTGAGAATGGGTTTATGGAGGAGACTAGAGAGTTTACCTTACGATGATGTACTTTCGGAGCGAGGAGGGGCTTCTGGGGTGTTGGTAAATGGGGTTTTGCATTGGATGGCAAGTAAAGCTTCTTCATTCGTAATTATTGGTTTTGATTTAAGTGATGAGAAATTTTTCGAGGTACCAGCACCTACTAATCTTTATGGAAATGAGTTGGATTGGTATGAACTTAGGAGTTTTAGAGGGTGTCTTTGTATGTTTTGTGCTTTATTAGAAAGCGAAATTGATGTTTGGGTGATGAAAGAGTATCGAGTTGAGGAGTCTTGGACTACATTTAGGATTGATAGAATGGATTTAGAGAATGGTTCGGTACCATTTTGTCCGATTAGTGATGATGATGTTGTATTGAGTGTGGATAGGGATAGGTTGACTGTCTACAACATCAAAGAGGATCAATGGAGATATATGGAGGTAGATGGATTAACTTATATGTTTGAAAGGACTGGAATTTTCATCGAGAGTCTTGTCTTGCCGATGCTTGGCAAGGGAACTGAGGGTTACCATATTGCTTGA

>StKFB06

ATGCAGTCCTGCAAAGCCGCTCCCAGTAACCCACAGGGTGCTGGAACAAACACGGAACAGGTGTATTCTGAATCGGAGAATTTTGTTGTGACAGAAAATAGTTTGGATAATGACCAGTCTGTGGATGTTGAGGACAGTGGTTCAGGGGATAATAGTGAGTTGGATCCAGAGGCATCACGATTAATTCCTGGGCTTCCTGATGACATTGCACTTTTCTGCTTGGCAAGGGTTCCTCGAAGGCATCATGTGCTTCTGAAATGTGTGTCAAGAAAATGGAGAGACTTGGTTAGTGGTGAAGAGTGGTACTCCTATAGAAAAAAACATGATCTCCAGGAGAGTTGGATTTATGCCTTAGGTAGAGACAAGTCTGAACAGCTGTGTTGTTATGTTTTGGATCCAACTCGATTAAAAAGGGGATGGAAGCCCATTCTAGGACTTCCACATCGCTGCATAAGGAGAAAAGGTGTAGGCTTTGAAGTGTTAGGGAAGAAACTATTCCTATTCGGTGGATGTGGCTGGATTGAAGATGCTACTAACGAAGTCTATTGCTATGATGCTGCCATGAATAAGTGGAATCAAGCTACTTCCTTGGTGGTTCCAAGGTGCTACTCTGTATCTGAAGTGTTAGATGAAAAAATCTATGCAATTGGGGGAATAGGGCCAAATTCCAATAATTTACCTTCCTGGGAAACTTACAACACGGAAACAACAAGTTGGACTTTGCATGAAAACCCTAACATTTTCCCTGATATTGAAGATTCTATAGTCTTGGGTGGGAAGATTTACATTCGCGGGGGTTCTTCACCTCTATCTTCTTTAGTGTCTGCATTTGTCTATGAACGATCAAGCAACACATGGCAGCCAGCAGCTTCTGAATTAGTATCAGGTTGGTATGGTCCAGCAGTTGTCGTAGATGGGACGCTTTATGTGTTAGATCAGTCTTCAGGTACCAGGTTGATGATGTGGCAAAAGGATATTAGGGAATGGGTGGCAGTTGGGAGATTGTCACCCCTATTGACAAAGCCTCCCTGTCGGCTTGTGGCGGTGGGGAATAATATCTTTATTATTGGAAAGGGCCTTAGCACTGTCGTTTTTAATGTTGAAAACGCAAGGAACATGGATGGTGTGTTAGTGAGCACTTCCATTCCAAAGTCAATATCTGACGATGATGTAATAAGTTGTAAAGCAATCACAATCTAA

>StKFB07

ATGGAAGGGGAAACCTCTTGGGTCAGTCATTGCCCTGATTACGTTGTACCAGCCATGGTTGAGTTTGATTCATTTTCAGAACTTAACGATGAAGAAAATAGAGAAGCTTCCTCAGTTCCTGTGGATTTGATACTGCCTGATGATTTACTGGAACGAATACTGGCCTATCTTCCCATTGCTAGCATTTTTAGGGCAAGTTGTGTGTGTAAAAGATGGTATGAGATAGTGAGTTCAAGAAGGTTCTTATGGAACTTCTCTCAGGTGTTGTCTCAAAAACCGTGGTACTTTATGTTCACAAGCTCAGAGGAGCCAGTTGGTTATGCCTATGATCCTTCCCTTCGAAAATGGTATAGTATTGACCTCCCTTGCATTCAGACATCCAATTGGTTCATTGCTTCTTCATGTGGATTAGTTTGCATCATGGACAATGACAGTAGAAGCGAACTATATGTTTGTAACCCAATAACCAAATGCAGCAAGAACCTTCAGGAGCCTCCTGGTCTCAAGTTTTCTGATTACAGTGCATTGGCTATCTGTGCAAGCATGAAAACTTTTTGTTACAGTGTCGCCATTGTTAAATCTAAGCAAGTACCAGGTAACTTCTATCAGTGGGATCTCTCAATCCACATATATGATTCTGGAACAATGAAGTGGTTGACCCCTTTGACTGAGGTTCTAACAGGCTGGAGAGGTGGGGATGAAAGTGTCATCTGTGATGGTGTTTTGTACTTCCTGATCTATGCAACTGGAGGTGGTGGTCTAGAAAGTCGTCACGGTCTGATCACTTACAACCTCTCAAGCAGATCATCCCATTGTTCGTTAATAAAGACTTTCATTCCCGTGCCATGTTCTCTAACATGTGGCCGATTAATGAACCTCAAGGAAAAGTTAGTAATGGTGGGAGGGATTGGGAAACCAGATCGTCCTGACATAATTAAGGGGATTGGCATATGGGTACTTAAGGGGACAGAATGGCAAGAAATTTCCCGCATGCCACACAAGTATTTTCAAGGTTTTGGGGAATTCGATGATGTTTTTGCCAGCAGTGGCACTGATGATCTCATATACATTCAGAGTTATGGAGCTCCTGCTCTTCTTGTTTTTGATGTGAACCAGAAACAGTGGAGGTGGTCACAGAAATGTCCCGTGACAAAGAGGTTTCCCCTTCAGCTCTTTACTGGTTTCTGCTTCGAGCCAAGGCTTGAGATGTCTCCCTGA

>StKFB08

ATGGGTGGCATATTGAGTCGGAGTAACCACAACTCTAATGTGGGGGATCTCATTGAGGGCTCCCAAAGTGCATCATGCAAGAGACAAAGGACATCAGATAGCTTCTGGGAGCATAGCCCACGATTGATTCCAAGTCTGCCTGATGAAATATCTATTCAAATCCTCGCCAGACTTCCTAGAATACACCACTTAAATGCAAAATTAGTTTCGCCTAGCTGGAAAGGTGCTATTATGAGTCCAGAACTTTATAGGTGTAGAAAAAAACTTGGAACAACAGAAGTGTGGCTCTATCTTTTGACAAAGACTGAAGGTGATAAGTTTTTGTGGTATGCTTTTGATCCGATCTCCGTGAGATGGCAAAAGTTGCCACCAATGCCCGCAATTGCGGTCAATGATGAACCTAAAAGTGGTTTATCTGGAATTCGGGCATGGAACATGGCAGGTTCAAGTATAAGAATTGCTGATGCCATAAGGGGTTGGCTTGGAAGGAGAAATGCTCTGGATCAAGTTCCATTTTGTGGTTGTGCTATTGGAGCTGTTGATGGATGCCTCTATGCCCTTGGGGGATTCTTCAGAGCTGCAGCCATGAGATCTGTCTGGCGTTATGACCCCATTGTAAATGCCTGGAATGAAGTAAGTCCCATGTCTACTGCAAGAGCTTATTGCAAGACAGGTGTCTTGAATGGAAAACTTTATGTAGTAGGAGGTGTTACTCGGGATCGTGGGGGACTTACTCCCCTTCAATCTGCAGAAGTATTTAATCCGCATACTGGTATCTGGTCTGAAATCCCAAGCATGCCATTTTCAAAAGCTCAGATGCTACCCACTGCTTTTCTAGCTGATCTACTCAAGCCTATAGCTACAGGGATGACATCTTATCGGGGTAAACTATATGTTCCTCAAAGTTTGTATTGCTGGCCTTTTTTTGTTGATGTTGGAGGAGAAGTATATGATCCCGAAACAAATGCATGGATTGATATGCCACTTGGCATGGGAGATGGCTGGCCTGCTAGGCAGGCAGGAACAAAGTTGAGTGTCACTGTTGAAGGAGAGTTGTATGCGTTGGATCCTTCTAGTACTCTAGATAGTGCTAGAATAAAAGTTTATGATCATCAAGATGACACTTGGAAGGTGATTGAAGGAGATATACCTATTAATGACAATTCAGAGTCTCCTTATCTTCTTGCTGGTTTTCTTGGAAAGCTCCATGTGATCACTAAAGATGCAAATCACAATATCATGGTCATGCAGGCTGATAGGCAAAATCATTCAGCTCCTTCTCCATCAACCTCAGCAAACTCTTCACAAAAAAACTTACACGAAGTTCCAGAACCGGTTTTAGGATCTGAAGCGAATATGTGGAGAGTTGTTGCTTTGAGAAGTGGTGGAACTGCAGAGCTGGTTAGCTGCCAAATCCTAGATGTATAG

>StKFB09

ATGCAAAGAGTTAGGGTATCATCACATCAAGCTCCAGTGCAAAGGCTAGGAGATTCACAAATGACATTGTCTCCAAAGTTTAGGTTAGCTGCAAAGCAATCTGATTTACTTGATCCTTCATTTGACTTAGAAATGTGGCGAAAGGGAGAACCCTTAATTCCTGGACTTCCAGATGATGTTGCTCTCAACTGTCTCCTTAGGATACCTGTTGATGACCATATGAACTGCAGGGTTGTATGTAAGCGATGGTATTCGCTCTTTGCTACGAAAGATAGATTCTTTTCTCGAAGGAAGGAGCTTGGGTTTCATGATCCTTGGCTTTTTGTCTTTGCCTTTCATAAAACTAGTGGGAAAATTCAGTGGAAAGTTTTTGATCTAAAAAACTCTTCTTGGCATACTATCCCTGCGATGCCGTGCAAGGAAAAAGTGTGCCCCCATGGATTTAGGTGTATCTGCTTTCCCCATGATGGTGTTCTCTATGTTTGTGGTGGGGTAGCCTCTGATGTTGATTGTCCCCTTAATTTGGTGGTTAAGTATGAAGTTCGGAGAAACCGTTGGACTGTTATGAAGAAGATGATCACAGCTAGGTCCTTTTTTGCCAGTGGAGTTATTGATGGGATGATTTATGTTGCTGGAGGAAACAGCACACATCTTTTTGAGCTGGACTCTGCTGAAGTCTTGGATCCTAATAAAGGAATTTGGTGCCCTGTTGCAAGTATGGGAACAAATATGGCCTCATATGACTCTGCAGTTCTTAATGGGAAGCTTCTAGTAACTGAAGGCTGGTTTTGGCCATTTTATGTTGTGCCTAGGGGCCAGATTTATGACCCTCAAACGGGTAATTGGGAGAACATGGCTTCTGGACTTCGAGAAGGTTGGACTGGTTCTAGTGTTGTGCTATATGGGCAATTGTTTGTAGTTTCCGAACACGAACGCACAAAACTTAAGGTTTATGATCCGGAAACTGATTCCTGGGACACTGTTGAAGGACTTCCACTGCCAGAGCAGATATGTAAACCTTTCTCTGTAGATTGCTGTGACAACCGGATTGTTGTCGTAGGCCGAAATCTTCATGTTGCTGTAGGACATATCAAGAGCCTGCAGCCATCTAGCAAACGTTGCTCATTTGCTGTTTACTGGCAAGTGGTAGATGCTCCTGCATCCCTCTCTGACTTCACGCCGTCTAGTGCACAGGTTCTATTTGCTTAG

>StKFB10

ATGGATGGCCTCCCCCATTTGCCTGGAGATATTGTCAACTCCATCTTCTTTAAGCTTCCTGTGAAATCTCTTATACGATTCAAAAGTTGTTGCAAATCATGGTATGGTTGTATCGATGACTCCGACTTCATCAAGTCACATCTACATAAATCGTCTATTGATATTAGTCGTAAAAAATTTGTCTTGGTTAATTCAATCCTTCTACATAGAGAAGGAACACGCAAATTTAAAATTGTGTCAACGGAAGCATCAATTAATGCTGATTCAAAAGTTGTGTATCTGAATATCCCAGAATACTTTAGTGACTATTTTTCATTACAAGTGTTTTCATGCAGTGGCTTGATCTTTATGACGTCATACGATCTTGGCTATTGTATGACATTATTGAATCCTGTGGTTGGAAAATACAAACTCATTCAAAATTCTCTTTTCAGTCAAAACACAAAGACAAATCGTTGTTCCACATCTCCAATATTTGGTTTTGCCTATGATTTTGTGGCTGAAGATTACAAGGTTATATGTGCACATTACTTGATAAACAAATACTTCAACGTTGTTGAAGTATACTCGGTTAAAAATCAATGTTGGAGAGCAATTCACAATACTTTTCCTGTTTCCCCTGATTCTTATAACCAGCACCTATACAGTAATCAAGTTTCATTAAACGGTGTCATTCACAGGATGTCATACAACAGGGCAGTTATATCTTTCCATCTAGTAGACGAAAAAATTTACCTGTAA

>StKFB11

ATGCCTCCTAAAGGCAAAGGAAATGGGAAGAAGAAAGGTAAAAGTAAAGGTAAAGGAAAAGGGACTTCAAAAGAACCAAAATGCAGAGCTGCAGCAGAACCAGAACCAACTTCCCACTTCTATTTCCCAAGAGAAATCATCTCTAACATCCTTTCCCGTCTCCCTGTCAAAACCCTTTTACGATTCAGGTGTGTTAGCAAGCAATGGCGAAACCTCATTTCCAAACCCGATTTCATCGCCTCACATTTCCGTCACTCTTCTTCTTTGCAGTTCTCCGGTTCATCTATTCTTATAGGCAGCCGTCATCGCGAGTCTAATCATCATGTAGTCTCACTATACAACCCGCCGGAATCAGTTGTCCAGGTGGACAGCCCTTTCCCCTGTTTCTTCCCCAATATGTACATTGTGGGTCCTTGCAATGGCTTTATCTGCCTTTTTAATCCACCATGGGGTGAACTGATTACCCTTTGGAACCCGGCGATGAGGAAGTATAAGATGGTGGAGCTCACTGATAGCTTGCCCCGTCAGGGACTGCACTTTTTGGCGTCTATTGGAATGGCTTTTGATTTCCAACATAATGATTTGTTGATCTTGAGAATATTTTGTGTGGGCATAATGTATGCAGTCCCGAACCATGTTGAGATGTATTCGAGTAAGAGTGGGAAATGGAAGAAGCTGAAAAATGAGATGATTTTTCATATTCTTGAGTTTACTTGCAATGTGATTGTTAAAGGGGTGGCGTATTGGTTGGTTTGTATGCCTGATAAGTTCGGATCGCGTGCTGTGTTTGTGCGATTTGATGTGGGGAAACTAGTTTTTGAGAAGTTACCTTCGATAGGAAGGCGTAAGAAGCATCAATATCTTGTGGAATTGGAGGGTTCTCTTTGTATGTTAGACTGGGATCATAAAGATGATTGTCATATGGATGTTTGGGTAATGGATGATGTAGATGGTTGGAGTAAGAAATACAGTGTTGGACCGTTAGTCGGGTTTGACCTAATATTGGGCTGTTTGAGGAATGGTGACATTGTAGCTAAGAATGAAAATGGAGTGATATTCTTGTGTGATCCCATAACTAGTTCAATCAAGGAAAAATTCAGCTTTGATAATAATAAGGATGGATCATATGTGATTGTTGATTATTCAGAGAGCCTATTTCTGATTGGAGGGATGCTACCTGTTAAGAAGCAAGATGCTCAAGATAAATTGGCGCGCAAAAGAGTCACAAGGAACTCCGGGAACTTAGCACATTAA

>StKFB12

ATGGTTGAAATAGCGGAATCTTCGGAATCTGGGTCTGGGTTGTTGAACTCGACACAGGTAAAAATTGGGTCTTTGCCAGAAGAGGACAGTATTCACTGGCAAGTTAGCAGCTTTGGAAGTAGTGGCTCCAGGAATACAAGCCCATTAGGGCGTATTGGATCAAGAAACACAAGCCCTTCAAGGCAGAAAGTGGTTAAGACGAAACCAAGGGGATTGGATGAAGAGACTGTAACCACTTTTGCTAAAGCGGTTCAACCAGATGTTCAAATGGAAGATAATATCTGGGCCATGCTCCCAGAAGATTTGCTGAATGAAATCTTAGCTAGAGTTCCACCGTTCATGATATTTCGGCTCCGATCTGTTTGTAAAAGGTGGAATTCAATTTTGCAAGATCATAGCTTTTTGAGGTTTCACTCCCAAGTGCCCTCCCATGGACCCTGCCTTCTTACATTTTGGAAGAATTCACAGACCCCTCAGTGTTCGGTATTTAGCTTGCCACTGAAACAGTGGTTTAGAATACCATTCACTTTCTTACCACAGTGGGCATTTTGGCTAGTTGGGTCTTCAGGAGGTCTTGTTTGTTTCTCAGGATTAGATGTGTTGACATTCAAAACTTTAGTTTGTAATCCCTTGACACAGACTTGGAGGACATTGCCTAGCATGCATTATAATCAGCAGAGGCAGTTGCTTATGGTTGTTGATAGAAAGGATAGGTCATTTAAAGTTATTGCCACCAGTGATATTTATGGTGACAGGTCTTTGCCAACGGAGGTTTATGATTCAAAGATCGACAAATGGTCGCTTCACCAGACAATGCCTGCTGTAAACCTTTGTTCCTCCAAGATGGCATTTTGTGATTCAAGGCTGTATTTGGAAACTCTCTCCCCACTTGGTTTGATGATGTATCGGCTGGATACAGGGCAGTGGGAACACATCCCTGCTAAGTTTCCACGCTCATTGTTGGATGGGTACCTAGTTGCTGGAACTCACAAACGCCTATTTTTGGTTGGAAGGATAGGGCTCTATAGTACTCTTCAAAGTATGAGGATATGGGAACTAGATCACACAAAAGTTGTCTGGGTTGAGATAAGCAGGATGCCTCCAAGATATTTTCGTGCACTTTTAAGACTATCTGCTGAAAGATTTGAATGCTTTGGACAGGATAACCTAATATGTTTCACATCTTGGAACCAAGGAAAAGGCCTTCTTTATGATGTCGATAAGAAGGCGTGGTCTTGGATTGCTGGGTGTGCTCTTCAATCATATAACAGCCAAGTCTGCTTCTATGAGCCAAGATTTGATGCTTCAATATACTAA

>StKFB13

ATGTGGAGCAACCTGCCTTTTGAACTCCTAGCCAACATTTTCTCTTATCTTTCTCCTGATTCATTAGCCAGGGCTAAATCCACCTGCAAGAGTTGGCACACATGTGCTAACAATTCACTTTCATGGGCAACGCTGCCATGGCGGCAATATCCGCCGTGGTTTCTAGCATTGCCCACACGCAACCATGGGCATTTTATTTGTGCTCACAACCCAATTAAGGATTCTTGGCATCTATTGCCTCTTGACTTCATTCCCAACCCAATTCGCCCAATTGCAGCAGTTAATGGCCTAATACTACTGAGAGAAACCACAACTACTGCTCTTCAATTAGCCATATGCAACCCTTTCACTCGCCAATTCAGGAACCTCCCAAAGCTAAATGTCACGAGAACTAATCCAGCTGTTGGGGTAATATCACTGAATTCAGCAAACTTCCAGGTCTATGTTGCCGGAGGGATGTCAGAGGCAAGCAGCGTCGGAGGAGGTGCTTCGTACGAGCCCAGCTTAGAAGTGTACGACTCTGTTCACGAAAACTGGAAAACAATAGGATCAATGCCGATGGAATTTGCAGTAAGGCTAACAGTTTGGACCCCAAATGAGAGTGTTTACTGCAACGGCATCCTGTATTGGATCACCTCAGCCCGGGCTTATACCGTAATGGGATTCGAAATTAGGAACAAAAATTGGAGAGAATTAGGAGTGCCGATGGCTGACAGGCTTGAATTTGCAGCACTGGTAGAAAGAAATGGGAAATTATGTCTTGTTGGTGGAACTAGTGATGCAGGGGCATGTATATGGCAGCTTGAGGAATCAAATAATTGGAGAATGATTGAGAAGGTGCCACAAGAATTGTGGGCAAAATTGTTTGGAGGTAAAGGAAGATGGGGTAGTATTAACACCAGATGTGTGTGCATTGGTGGGGCAATGTGCTTGTACAGAGATCTTGGATCAGGAATGTTGGTATGGAGAGAATGTGCAGAAAATGGTACTAAATGGGAATGGCATTGGATTGAAGGGTGTGGTACAATTAAAGGGGTGCACTTGCAGAATTTCCCCATTAAAGGACTGTTGCTACATCCCTATCTTGCATCCTCCAACTTCTTGCTGAATGAATGA

>StKFB14

ATGAGACGGCGTTCAAAGACTAATCGGCGTCGGCGTGTTGTGGAGAAGAAAAAGAAACAATTTTTTTTCAAAGATCTCAGTGATGAACTGCTGATCGAAATACTTATCAGATTACCAAGCAGCAAAGAAGCAACTCTATGCAAATCCGTTTGCAAACGCTGGTTTGCCCTAATCTCATCTGATAACTTCCGTAAAATCTCTCTAACTCATAATCGCAATTGCGACAAAAAAACACTCATCCCCTTTACTTTTGTTTCAATTGATTACAACTATTTTCATTATCTTTCAGATGATACGGATTTATATGTATCTGAGTTTAGCCCGGAAAATGGTTTTTCAAGACGGGTCAATTCAGGGTTTCTCTATTCGAATCTCCCCCCTGTGAACTATATTAGTTTGATAGAATCATGTGGTGACTTAATTTATTGTTCTGGAGGGACATCTGATCGTATTGATTATTATATTGTTAATGTTCTGACCAAGCAGTGGTTTCTGCTTCCTCGGACTCCTCTAGAAAGCAACACACATTTTATGAGTACTAGTGAAAGAGTTGGTTTCTTGATTGAGCCTAGTTCTGTGGATAATGCTCCATGCCAGTATCTGGTGTTAATGTTTATTTCTTGGGGTGATTCCAAGTTTAGTATCCACGTGTTTTCGTCTAAGAAAGGTAACTGGACCAGAATGGTTGTTACATCTCCAAGGAATTTGAATATGTTAACACGTAGGACTTCGATTGTTGCCTGTGGAAGGATGTTTTATACATTCACTTATGAGAGAAATGATGTGGTTGACTGTGTTCTTGCATTTGATCCATTCACTAATGATCCTGCTCAGTTTCTATCTGTTATTGATTTCCCACCTGAGGCTCGTGATAAACCTTGTTTGACTTGTAAATTGGGGGTGTGTGGAGGTCGTCTACGGTTTGCTCGAATAGTTCTCCTACCAAGTAGATACCTTTACCCTTGTATAAGTATATGGGAACTTGAGGATGATTACAGAACAGGGAAATGGACATTGGTGCACCAGAGAGTCCCTACTGACACAGTGTTTAGAGTCCCTACACTTGCCACAAAATGGGTTTCTGTACTAACTTTTCACCCATATAATGAGGATCTTATATGTTTTCTTGTAGGCAATGATCATATCGTGTACAATATACAAACAGATAAATTGGAAAGCTCTACCCTCACTTCTCTATTCAAGAAATCACTAGATGTTCATGTGGTACCAATTACCCGCAATTGGTGGCCAACATCCCTTTGA

>StKFB15

ATGGATCTTCTTCCGGGTCTTCCTAATGATATTGCTCTTGAATGTCTAATTCGTCTTCCTCTCCATCAATTCTCTAAAGCTGCTTCCGTATGCACCAGCTGGAAAACCGAGATCAAGCATCCCCTGTTTCGACAACGTCGGAAAGAATCGGGTCTTACCCGACCCGTTTTCGTATTAGCCCAAGCCATGGTTACTACCATTCGAAAACCTTATGGCATTACCAGCCTTTCATCTACTCAGTTCTATAGACTCACCCTTTATGATCCGGAAAGGGGATGTTGGTACGATTTGCCGCCGATACCGGAGTTAATTGATGGATTGCCCATGTTTTGCCGGGTTGTAGGAGTCGGATCGGATGTAATGGTGATAGGCGGGTGTGACCCGGTTAATTGGAGGGTTATGGACTCTGTTTTCATCTACAATTTCGTATCCGGTTCGTGGCGTCGTGGGACGGATATGCCGGGAGGACAGAGGCTTTTTTTTGGATGTGCGTCGGATTCGGAGAGATTTGTGGTTGTCGCCGGCGGACATAACGACGAGAAGAATGCGCTGAGATCGGCTCTGTTGTACGACGTGGCGGAAGATGAGTGGATTACACTGCCGGAAATGGCCACTGAGCGTGACGAGTGCAAGTGCGCATTTCACCGCGGTGAATTCCACGTCATCGGAGGCTATCCTACGCATGCACAAGGTCAATTCCATCGCAGCGCTGAGGTATTCAACTCCGACACGTCCCGGCAGTGGCGTCTGGAGGAGGACTTCCTGGGGGCCGACACGTGTCCCCAGACCTGCATAGAAGGCGACGACGGGAGACTCTACATGTGCCGAGACGGTGACGTGGTCGTAAAGATAAACGCCACGTGGAAACACGTGGCGAGACTGCCAGGTGGAGTATCCAACGGGGCGTATCTGACAGCGTGGCAGGGGAAGCTGTTAGCAGTGGGAAATTCAATATTAGATGAAATTCACAGTAGCTATGAACTTGATGTGAACAGTGAGAGTAAAGAGAAGACATGGAGAAAATTGGATGCTCCAAATGAATACTGTGGACATGTTCAATCAGTTTGTTGGTTAGAGATATGA

>StKFB16

ATGGAGATTATTCCCACTCTTCCTTACGATATCGGACTCGAATGTCTTATTCGAGTTCCTTATTATAATTTCTCATCGGTTACTTCCGTCAGTAGAAACTGGAAGCTTCAGATTGAGCTTCCCGAGTTTTGGAGGCGAAGAAGAGCTACTGGTTCCACCCGTCAGGTAATTTTAATGGCGCAAGCCCGAATTGACCCGAGATTGAAACTCGGGTCATTCAAGTACTCTGCTTTTTCGGTTTACAAGCTTACGCTTTACGAACCGGAATCGGGTTATTGGGCGGAACTACCACCGGTTCCTGGTATTTCCGATGGATTGCCGATGTTTTGTCAGCTCGTCGGAGTTGGATTGAATCTGGTGGTGATGGGCGGGTGGAACCCTCTTACTTGGGAACCTTCAAACGCTGTTTTTGTTTTTAGCTTTGTGTCCGCCACGTGGCGACGTGGAGCCGACATGCCTGGTTGCCGGAGATCGTTTTTCGGTTGTGCGTCGGATTCTGAACGGACGGTGTACGTCGCCGGAGGTCACGACGAGGAGAAAAACGCACTTAAATCGGCGATGGCGTATGACGTGGCAAGGGATATGTGGGTCCCAATGCCTGACATGGCAAGTGAAAGAGATGAATGTAAATGCACTTTTTTTCAAGATAAATTCCACGTCATCGGCGGATATGACACGAGCATGCAAGGCCAGTTCGGTACAAGCGCTGAGTCATTCGATCCTTCCACGTGGCAGTGGGACCAAGTCAACGAACATTTCTTTGAATCTGCCACGTGTCCAAGAACCTGCGTTGAGGGTGGGGACGGAAAATTGTACTTGTGCCGGGACGGTGATGTGCTGGCACTCGGAAAGTCTACGTGGCAAGCTGTGGCGGCGATTCCGGTGGAGCTCCGGAGCGTAGCTTTTGTGACAGCATGGAGGGGTAAAATTCTGATGACTGGTTCTATGGGATTTAATGAACCCCACAATACTTATGTTCTTGATTTACAGAGTTATAAATGGACCAAAATGGATACTCCGGCGAACTTTTCCGGCCACGTTCAATCCGGCTGCTGCCTGGAGATGTAA

>StKFB17

ATGCCTCCTGTCCGAGCTAGAGCTAGGGGTGATACGCCTGCTACATCTGAAGCACCTCCTTTGGTGGCAGTTGTTAATAGTGAATTGTATGCTATTGGTTATGCTAACATGGAGGTGAGGAAGTATGACAAGAAAATTAAAGCATGGGCTACCATAAGAAGATTACCTAGAGCAGCTTCAATGGATGATCGGGGTTTGGCTTTTCGAGCATGCGGTGATAGGCTAATTGTAATTGGAGAGCCTATAGCCATGGGTGTAGGGCCTTCTGAAGTGAGTTCATCGGACGCCCTCAGTGGAACTATCTTTGATGAAAGCGATCAGGATGCTTTGTGTATAACTGTACTGTCATGGGTTGCTGATCATGAGATATTTGCTAATCGAGACAGAATGGCCTCTGTTTCATAG

>StKFB18

ATGGCGGATCTTAGCTTACAATCTCAGCTCATCCCTAACCTCCCAGACGACATTGCCTTACAGTGCTTAGCCAGAGTCCCACGTTCCCATCACCCCATTCTCTCCCTCGTCTCCAAATCTTGGCGTTGCATCCTAAGCTCCACTGCACTTTACACCACCCGATCAATCCTCCGTACTACCGAAACCTTCCTCTACCTTAATATCCGGGTAAACTCCACTTTCCACTGGTATACTCTTTTCCACAACCTTACTTTCACAAACCCAGAAAAACCCAGAAAGCTTTTTCCCCTCTCTTCAATACCCACTAAGCCGATTGGACCGGCTTTTGCAGTTTTGGGCTCGAGAATATATGTGATTGGTGGGTCTATTGGTGAAATTCCGTCGAATAATGTGTGGGTTTTTGATTGTAGGTTGAATTGTTGGGAAATGGGTCCGAGAATGAGAATTGGTAGAGAGTTTGCAGCAGCTGGTGTTGTGAATGGGAAGATTTATGTAATGGGTGGGTGTGTTGTTGATAATTGGGCGAGGTCGATGAATTGGGCTGAGGTTTTTGATCCGATGACGGGGTTGTGGACTGCGTTACCGAGTCCGATTGAAGTTAGGGATAAGTGGATGCATGCTAGTGCAGTTGTGGGTGAGAAAATGTATGCAATGGCGGATAGGGGAGGAGTGGTGTATGATGTAGGGGGATGTGAATGGGGAAGTGTGTCGAAAAGGCTTGATTTGGGTTGGAGGGGACGGGCGGCAGTGGTGGGAGGGGTGTTGTATTGTTATGATTATTTGGGTAAGATTAGAGGGTATGATGTGAAGGAAGATGTGTGGAAGGAACTGAAAGGGGTGGATAAGGGGTTGCCTAAGTTTCTGTGTGGTGCCACGATGGTGAATTTTGATGACAGGTTGTGTGTTGTGTGGGAAGGCAAGGGGAGGGGAAAGGAGGTTGACATTATGTGTGCAGAGATCGAAGTGTGGAAGGATGAAGATGGAGGGTTGAGTGGGAACATTTTGTGGTCGGATATGATTCTTGTTGTTCCTAATGGTGCCTCAATTGTGCAGTGCTTGGCAGTTGACTTGTGA

>StKFB19

ATGGAGTTTTCCCCTCTGAATCGTCTACCTCAAGATACCCTTCATCAGATCTTTTCCCATTTAACCCTCCGTGAAATAATCGTTTCCAAATGTGTCTGCAAATGTCTCAACACTACTCTCTCTTCTCCGGCGTTCCTCCACCTTATCTCCACCCAACAACCACCTCTCTCACTCCTTGCCCTCCGACCATCTCACCGTACCCACACTCATACACACAACAATTCATCTTCTCATTGTGCACTCCATGTGTTCGACACCATGCTTAACTACTGGTTCCGATTCCCTCTTTCTTTCCTTCCTTTTAGATCTCACTACCCCATCACTTCCTCTCATGGTCTTCTCTATCTCTGGGCTGAAGGACCCACTTCCGTTTCACCTCCTGGTAACAATAGCAAAACCCTAATCGTGTGTAACCCTTTAACTCGTCAATTCAAGCTGCTTCCTCAATTGGGTTCAGCTTGGTGCAAACATGGGTCGGTTCTTGTGGGTTCACCGAATCAAGTTCTAGTCTTGACTGAGCTAGCTGCTATTTACTTCTCTGGGTCGACAACTTCCAATAATTGGCTGAAATTCTCCTCAAATTTACCATCAAAACCTAGGAGTCCAATCTTGATTTCTGATACCATTTTAGCCCTTTGTGATGTTGGATCCCCGTGGAGGTCTCAATGGAAGCTGTTTAGGTCAACAGTTAAGGATTTGCAGTTTAGTCAACAATGGACTAGATTAGAAAAGCATGAATGGGGTGATATTTTTGATATAATGAAACGACCCAGATTGCTTGCTGGTAAAAATGATAAGGTGTTGATGATTGGTGGTTTGAAGTCGTCCTATTCGTTGCATAGCACATGCTCGACAATTTTGATACTTAGATTGGATATGGAGTCTTTGGAATGGGAAGAAGCTGGGCGAATGCCGCCTGAGATGTTTAGGTATTTTCAAGATTCTAGTAAGTTTAAGGTGTTTGGCGGAGGGAGTAGAGTTTGTTTTTCGGGTAAGAGGGTGGGAAGATTGGCGCTTTGGGAAGAAAATGAGTGTGGGAAAGGGGAATGGCGGTGGATCAGTGGCGTCCCTGGGAATAGTGATGGACTTTATCGTGGATTTGTGTTTGAGGCTCGGCTAAATGTGGTGCCTTAG

>StKFB20

ATGAACTACAACAGATATTCACTAACAATGGAAAACAAGAAAATGATACAATCAAATCAAATGGATCCCAAAATATGGAGTCGATTACCAGAAGATGTATTAGAGCATTTGCTTTCATTTCTTCCATTAAAAACATTCTTGAAACTCAGATCAACTTGTAAGCATTTCAAAACTCTTCTTTTTTCACCCCCTTTCATTTCTAAATCTTCTTCTTCTTCTTCTTCTTCTTCTTCTTCACCTTTTTCTTCATTTTTCTTACTTTCACATCCACAATTCCCTAGACAATACCCTTTATTCGATACTGTTCATAACAACTGGCGGAATTTATCTCTGTGTATTTCCCCTGTTTTGCCATCTTCTTCCTCTGTTCTTTTATCTTCTTCTAATGGCTTGCTTTGTTTCAATAGTTCAAATTCTAGTTCTTTCATTATAACTAATGTTTTAGCTAGATCTTCTAGGGTTGTAAAATACCCAAATTTGCCTTTTTCTTTTGAGTCTGTTACTTTGATTTCTTCATCTAATAATGGGTATAAGCTGTTTGTGATGTCTGCTTTTGGATCTTCAAGTCAAGTTTTTGTTTATGATTCTTTGGTTCATTCTTGGAGTCAATTTGGTGGATTTGATCTGATTTTAAATGAGAATCATCATCAAGAGGGGGTTTTTCATGATGGGTATTTGTGGTTTATTACACCAGAGCCTTATTTTACAGTTTGTATGGATCTTGATAATGGGGTGTGGAAAAGATCAAATTTTGAGCTGCCTAGTGAGGTTACTTTTGCTAGATTGGTGTGTGATGGGGATAAAAAAATGTTCTTGGTTAGTGGAAATGGGAGTAATGGGATTTCAAGAAGCATGAAGTTGTGGGAATTGAATGGGGATTCTAAGATTTGGGTTGAAGTTGAGAATGTGCCTGAATTGATTTGTAGGAAGTTTTTATCTGTTTGTTATCATAACTATGAACATGTTTATTGTTTTTGGCATAAAGGGTTGATTTGTGTTTGTTGTTATTCTTGGCCTGAGATATTGTATTATAAGGTTTCTAGAAGGACTTGGCATTGGCTTCCTAAATGCCCTTCATTGCCTGATAAATGGAGTTGTGGATTCAGATGGTTTTCCTTTGTTCCTGAGCTATATGCCTTTGTGTAA

>StKFB21

ATGGAGTCGCCGCCGCCGCCGCCAATGGAAACCTCGAAGCGCAGAGCAGCAGCAACAGCATCAATTGCTAGCTTAACCAATGATAATTCAGTTCTTCCTATAGAACTCATATTTTTTGAAATACTCATAAGGCTGCCTGTGAAAACCTTGCTAAAGATGAGATCTGTTTCAAAATCCTGGATTTCTCGCATCTCAACTCCCGAATTTGTCAAAGCACATCTAAATTTTTCAGCCAATAATCGAGAATTTGCTCACCACAGGGTTCTTAGTATCAGGTCAGGTTCTCACATTCACGACGACGGACATATTACTAGATGGAGATATTTCCGTACCTTTTCTCTTTACGCAATTCTATATGGAGAATCTCCCTGTTTTCCTGTTGAGCTTCATAATTTTGGTGTAAGTTATAATGTTTTGGGTTCATGTGATGGATTGTTCGTTATTTCAAAAACCTGGTATGATAATGACATTGAGAATCTGTTTCTATGGAATCCGTCCATTAGAAAGTTGAGTAAACTTCCTTATTCAGGAATTGATGCGCGCAAACGTAGATTTGCTTATGGATTTGGTTATATCGAATGTCAAAATGATTACCAGATTGTTGAAATTGTGGCAAGTAAACCTAGTTATCTCATTGCTGATATTAGTGTTTATAGTTTACGAAATAATTCGTGGAAAACCATTCAAGAGTTCCCAATCATCTCTTTGCCTGAAAATGTTAAATTTGTTAAAGGAAAGCTTCATTGGATCACTGGTGGTAGTAGCGGTAATAACGCGACATGGTTCAACCCCGGAGATGAGAAGTTTGGCAATGTAGCATTGCCGAACCCAAGTGGTGACACTTTTAACTGGAAATTTGTGTCTTCATCTGGTAATCTATGTATGACTTGTGACTATAGAAACAAGATAGATGTGTGGATAATGAAGGAGTATGGACTTGCAGAGTCATGGACCATTGTGGGTTCGATCCCTAAATTTGTAAATAAGGTGGTTCGGCCAATTTTCATCTCTCATAATGATGAAATTCTATTGCAAGACGTTTCAGGTTTATTGTGGTGGTATGTTTCAAGAGATGATGGCAGTTTTGATCGTCCTGAGGATCAAACACGTTGTGAATATGATCGTGGAAGTGAGCTTAATCTATACATTGAAAGTCTTGTTTCGCCAAATTCTCCTTGA

>StKFB22

ATGACATCAGAAAGATTAACAGGAGAAGAATCTCTTCAACAAGATCTGGAGTCTTTAAGCGTGTCTAAAAGGTTAGTCAGAAGTGTGAGCCAAAAGTTGAAAAAGAAGAACCATAGAAGTGGAGGAGAAGAAGAGGATGACAGCAAGGGTATTTCCTTGAGATGTCTAACGCTATATGGCAGAGGGGGTGGTTGCAAAGTGGGTGCTGACACTGGTGACGACCTAGGGGATTCATGCGGTAGAAGAAGATCAAATGCAAGTGAGGAAGGCAAAGGGTACAACCCGATATGTGGAAATGAAGAAACGTCAGTAGATTGCTTCTCTTATGGGATGAGGGAAAAATTCTGGAGGAGGTCTAATAGGAAATCTCTAGAGCTTGAAGCAGCACTCCAGAACAAAAGCATGAATGTGTTTTTGCCAGATGATATCCTCGAAATGTGCTTATTGAGGCTTCCATTTATAAGTCTCGTGAATGCTAGGATGGTCTGCAAGAAATGGAGAAACTTGACAATGACACCTCGTTTCTGGCGAATGAGGCAGGAAGGTTCGTTCCAGAGTCCATGGTTGTTTCTTTTTGGGGTTGTAAAAGATGGCTGTTGTTCCGCAGAAATACATGCATTTGATGTTTCCTTCAACCAATGGCACAAAATGAATTCTGAAGTTCTGAAAGGGAGGTTTTTATTCTCTGTTGCTGGTATCCATGATGATGTTTACGTTGCTGGAGGTTGTTCTAGCCTTGCTAACTTTGGGAAGGTTGATAAGAGCTCATTCAAGACACATAAAAGTGTGCTCGTTTTTAGTCCCTTGATGAGAACGTGGCGTAAAGCTGCACCGATGAAGCATGCAAGATCATCCCCTATTTTAGGAACTTATGAGATCAGTTCAGATTGTTTAATTATTAGGAATCAACAAACTCGAGGAGACAAAAGATTTTACCGTCCAAGAGTTGGTGGTGTATCTGATGTTTATGAAGATCCTCATAGACTTTCAGTGAGACGCCAATTTCGACATTCTCTGGATGAGAACGAAGTTACATTCTTTCCCAATGTGAAACCATACAAGTTTGTCAAACAAAAAACCGAACCTTCAAATAAAGATCAAAGACGCTTTCTCTTGATTGCTGTAGGAGGTCTCGGGTGCTGGGATGAGCCTCTGGATTCTGGGGAAATTTATGATTCCATGTCAAATAAATGGACAGAGATCCAGAGGCTTCCTGTAGATTTCGGAATAGCTTGTTCAGGGGTTGTGTGCAATGGGTTGTTTTATGTTTATTCAGAAACTGATAAGCTAGCTTCATATGACGTTGAGAAGGGCTATTGGGTTAGAATCCAAACTAGTCCATTCCCTCCCCGAGTTCATGAATACCACCCTAAACTTATATGCTGCAATAGTCGGCTATTCATGCTCTCTGTCTCCTGGTGTGAAGGGGAAGGTCAGATTGGCAGGAGAAACAAAGCAGTAAGGAAACTCTGGGAGCTAGATCTTATGCCTCTCACTTGGAGAGAAGTTTCAATCCATCCAGATGCCCCAATGGATTGGAATGCTGCATTTATAGCTGATAAAAACTCTATATTTGGAGTCGAAATGTTCAAAATATTCGGGCAGGTGCTAGACTTCTTGACCGTTGGAGATGTAACCGATGCTGGAATAAACTGGAGCCATATCTCAAGGAATCGTCTTGCGCAAGAATTGGATGCTGCTTCTTGCTTGACGAAATCCATGGCAGTACTGCATTTGTAA

>StKFB23

ATGGCAATTATTCTTGAGGATGAAGCCCCTATTCATGGAGATGTATTAGAAACTATTCTTTCACATGTGCCACTTGTCGACTTAGTTCCTTCTTCGTGCGTTTCAAAATCATGGAATCGAGCCGTTACTTCTTCTCTCAAATGCTTCAACAAGCCGAAGCCTTGGCTAATTATCCACACTCAATGCACTCGCTCACCTTATGACATTTCGGTACATGGTTACGACCCTCGCTCGAATGTCTGGGTCGAGATATCTCAACCATCTATTAAGTATGTCTCCGCACTCCGATCATCTCATTCGAATTTACTCTACATGCTTTCTCCATCGAAGCTATCGTTCTCACACGACGCGATGAATCTCACGTGGCATCACGTGGATGCACCGCGCGTGTGGAGAACCGATCCTATCGTGGGTTATGTAGGTGGCTCAATCGTTATAGCCGGTGGCACGTGCGACTTTGAAGATGATCCATTAGCTGTGGAAATTTACAATAACGAAACGAACACTTGGGAAACGTGTGAATCAATGCCGGCGATACTCAAAGACTCTGCGGCGTCTACATGGCTGTCAATCGCTACCACCGGAGATAAACTCGTCGTCGCCGAGAAATTCACCGGAGTTACTTACTGTTTCGATCCGAAAACTAAGAATTGGTCAGGTCCGTATGAGTTAAGGCCAGATCCACGTATTTTTCACTCGATTATCGGGTTCGCGAACAACCGATTGATCCTGATTGGAATGATCGGAGACTCTGAAAATGTGGCCGGAGTTAAAATTTGGAAAGTGAATACGGAGAATTTTGAGTGTGAGGAATTGGGGGAAATGCCGGCGGCGTTGATTCAGAAGGTTAAGAGTGAAACATATGGAGTTTCTTCGATTAGTGTTTGTTTAGCTGGAGATTATGCGTACATGAGCAAGTCGTCGGAGATGGCGGAGGAGATAGTAGGTTGTGAGTTCAAAAACGACGGCGGATTACGGTGGTGGAGCATGAAAAACGAGGCTGCGGGTGACGGAAATAGATCGCAGAGAGTGGTGTTTAGTTGTTCTGTTATTGGGTTAGGTGATTTGCAACGAGCTATGTTATTGGAAAATCGGAAATTTACTGTGAAATTGTGA

>StKFB24

ATGGAAGTGTTGGCCATGCTGAGGCAACTCATTGGACAAGTTAAACAACTGTTAGAACTTCAAGCTTCTTCATCTTCATCATCATCGCTTGTTGCTGTTGCTCCTAACATCTCTTTTCATCTTCAAACGCCACCACTTATACACCTACCAAGATGTTATTTTCTGAATCTTGATGACAATTCTGCTGAAGACAGTTGCTACAACATCATCATGACTGCTGGAAAATCTGAAAATCTCAAGATGTTGGAACCAGGAAAGCCTCCACCAAAAAAGAAAGGCCGGAAGGAGAGAAATCAGGGAAAAGTGACTGGAACTTCATGCTCCATCGAGAATTTGGATCAGCAAATTTGGAAAGAATTTCCTGAAGACCTATTTGAAGCGGTTGTTGCAAGACTACCAGTTGCCACTTTTTTCCGCTTCAGATTAGTTTGCCGCAAATGGAACTCAATGCTGATGTCCCAAAGTTTTTCTGAACAGTGTGCCCAAGTTCCTCAACCACAACCATGGTTCTACACCATTACTCATGAAAACGTGAATACTGGAGCAATGTATGACCCAACATTGAAGAAATGGCATCATCCTACTATACCTGCACTGCCAACCAAGTTGATAGTCTTGCCAGTTGCTTCTGCAGGAGGTCTTGTGTGTTTCCTTGATATTGGACATAGGAGCTTCTATGTATGCAACCCACTTACTAGGTCCTTCAAAGAGTTACCAGCCAGATCAGTTAAGGTGTGGTCCCGTGTGGCAGTAGGTATGACATTGTGTGGTAAATCAGCTGGCGGGGGATACAATATCCTTTGGGTTGGTTGTGATGGAGAGTTTGAAGTTTACGACTCCCGAAATAACTCTTGGGCTCGTCCAGGAACCATGAGCTCGAATATTAAGCTCCCTTTGGCACTCAACTTCAAGTCGCAGACAGTGTCCATCGGTAGTAAACTTTACTTCATGCGCTCGGACCCTGATGGGATCGTGTCCTATGACATGGTTACTGGGGTTTGGAAACAGTTCATTATCCCTGCCCCCCTACATTTGAGTGATCATACACTAGCAGAATGTGGGGGGCGCATAATGCTTGTGGGTCTGCTGACAAAGAATGCAGCCACTTGCGTGTGCATATGGGAGCTGCAAAAGATGACTCTTTTGTGGAAGGAGGTTGACAGAATGCCAAACATATGGTGCTTGGAGTTTTATGGAAAGCACGTTCGAATGACTTGCTTGGGTAACAAAGGTTTGCTCATGCTATCGCTAAGATCAAGACAAATGAATCGTCTAGTTACATATGATTTCTCAAGCAGGGAATGGATGAAGGTCCCTGGTTGTGTTTTGCCACGTGGGAGAAAGAGGCAATGGATCGCATGTGGGACTGCTTTTCATCCCTGTCTAACAGCTTTGGCTTGA

>StKFB25

ATGCTATATCTCCCAAATGAAATTATCTTTGAAATTCTCCTAAGATTACCTGTCAAATCCATCATTAAATTCAAATCTGTTTCGAAATCCTGGCTTTCTTTACTATCATCTCCTCGATTCATCAATACCCATCTCAATTTTTCTAAAAACAACCACAAAAATGTCCCTCAAAAACTCCTTCTATTAACCCCTAACCAAAATTTATCTAAAAAACAATATACCCTTTTCTCCTCTATTTCTGAAACTATCGTCCGTGTTGATCTTGATTATCCTGTAAAATCCCCTAGTTGTGTTACGCAATTTATCGGATCGTGTGATGGGTTGATTTGTTTTTCAGTAGAAAATTCACTTATTTTGTGGAACCCCTCTACTAGAAAATGGAAAAAAATTCCAAAAGAATCTATTTTTATGAATCAAGATTATTATTGTACGTATGGTTTTGGTTACGATGAGTTTAATGATGATTATAAATTGATTTTAGTCTATAGTTCTAAAATCAAGAATATTGGTTATAATGAGGTTAAGGTTTATAGTTTAAAGACTAATTCTTGGAAAAGAATTAAAGGGTTTGTTAATGGTTATGTTTATAGTAATTCTGGTGTTTTAATGAATGGTATTATTCATTGGGATTCGCGCCCACATCGCGATTTCAATGGCAATGACTCGAAAATCGTCTACTTTAATTTGGTAAGTGAAAAACTTGGGAAGCTAGATTTACCTAGCTATGATGAAAATGAGGATGTTGTTTGGGATTTAATGTCATCTAAAGAATCTTTATTTGGATTTTGCCATAGTGAATCTCAAGGCGCGGTTGATATATGGGTAATGAAAGAGTATGGTATTAAGGAATCTTGGATTAAATTCGCGTCAGTTCCTTACTATGTAGTTCCGGGGATTTTTGATACATCTATGTTTATAAATGAGGACGGTGAGGTATTATTGATAGATGGGGAACGATTGGTGTTATTCAATACAAGAAATAATATTTACAAGGATCTTCGTATTCATATACCTGATACTCGTCGTAGAATTGATATGGCTACTTATAATGAGACCCTTGTTTCACCTGTTTTCGATGATGAAGATGGATGCGAGTTCTGGTAA

>StKFB26

ATGACTGCACTCATCGAAGGTCTTCCTGATGCTGTTGCCATTAGGTGCCTTGCACGGGTTCCATTCTACCTTCATCCAAAGTTAGAGCTTGTTTCCCATTCCTGGAGAGCTGCTATTCAAAGTGCTGAACTATTTAAAGCTAGACAGGAGGTCAACTCATCTGAAGAATTTTTATGTGTATCCGCATTTGAACCTGAAAACTTATGGGAGCTCTATGATCCTACACATGACCTTTGGATTACTCTCCCGATTCTCCCCTCCAACATCAGTCATTTTGCTCGCTTCAGTGTTGTTTCTACTGCTGGGAAGCTGTTTGTTTTAGGTGGTGGCAGTGATGCTGTGGATCCATTGACAGGTGACCAAGATGGAATTTTTGCAACTGACAAGGTCTGGTCATATGATCCTGCAACCCGAGCATGGAATCCGCGTGCATCTATGCTTGTTCCTCGTGCCATGTTTGCTTGTTGCGTGTTGGATGGGAAGATAGTCGCTGCAGGGGGTTTTACTAACCGCAGAAAATCAATATGCAATGCGGAAATCTATGATCCTGAGACGGATGTCTGGGTACAATTACCTGATCTCCATCATGCGCACAATTCTGCTTGCTCGGGAGTAGTTTTTGGTGGTAAAGTTCATGTCTTGCATAAAGGTTTGTCAACTATTCAGGTTTTAGAAAATTTCAAGCAGGGTTGGATTGCTCACGAGTATTCTTGGCTCCAGGGCCCGATGACTGTCGTTAGGGAAAATCTTTATGTTATGAGCAATTGGTTCATTTATAAGCAAGAAGGAGAATTAAGAAGAATGATAGTTTCAGCATCTGATTTTCGTAGGAGAATTGGGTATGCTATGATAGGGTTGGGAGATGATATTTATATAGTTGGAGGGGTTAATGGACCGGATTACTGGAATTGTAGCGTCAAAGTGCTGTCTGATGTTGATGTCTTGACCCTTGGAAGTGAGAGGCCAGCGTGGCGTAAGGTTGCTCCATTGACAAGGTGCAAAGGGACAATCCTTGGCTGCACACAGCTGAGAATTTAG

>StKFB27

ATGGCTACAAGCAGCAGCTGCAGTTCCCCTTATACCAACGGATCATCTCCAATAACGACAATAGCTCAAGACCATCTCTTCTCCATCTTACTCCTCTTACCTTTGGACTCCATTTTTTGCTTTGCTCTAACTTGTAAGAAGTTCAGGTCCCTCACTTACTCAGACTCTTTATGGGAATCACTCTGCCGAAGAGATTGGGGAAACTCCACCATTGATGCACTCAGATCCTTTGCTGTTGACGGTAAACAACAACAATTTCCTTGGAAGAAGCTCTACCAGCAAATCTACCAATTGGACTCTGTCTACTGTCGCAGACTGTTGACGCATCCACAAGGAGGAGAAGAGCTGCTTCTTCCTCGACCTAGAGCTTCTCATTCTCTCAATTTTGTTTCTGGTTGTCTTGTTCTCTTCGGCGGCGGCTGCGAGGGAGGAAGGCATCTGGACGATACATGGGTAGCATACGCTGGCAATGACTTCAAGAGAATATTGGAATGGAATAAGATTGAATCAGGGGTACCAAGTGGCCGCTTTGGGCATTCATGTGTCGTGATTGGCGATTATCTAGTGCTTTTTGGAGGAATAAATGATCATGGGGCCCGCCAAAATGATACATGGATTGGCCAAGTAGCGGTACACGATGCATTTGGTATCACATTATCCTGGAGACTACTTGATGTCGGTTCCATTGTGCCTCCACCAAGAGGCGCTCATGCTGCATGTAGCATGGATAAAAGGAGGATGCTGATTCATGGAGGGATTGGTCTGTCTGGCTTGCGATTGGGAGATACATGGGTATTGGAACTGTCCGAGAATCTTCATCTTGGAGTTTGGCAAGAAATTGTGACTCATCCATCTCCTCCATCTCGCTCTGGACATACATTGACTCCTGTTGGAGGAAACCAAACAATTTTGTTTGGTGGGAGGGGTTTAGGCTATGAGGTGCTTAATGATGTATGGCTGTTTGATACATCTGATGGTCACTGGAGATGGGTACAGTTACTATTTGACTTGCAAAATATACCCCAAGGATTGACCCTGCCAAGAGTTGGTCACTCGGCTAATCTCATCATAGGTGGACGATTGCTAATTTATGGAGGAGAAGATTCCTACAGACACAGGAAAAATGACTTCTGGGTGTTGGATATCAGCTCAGTGACGTCCATTATGCAGTCTGGGTCTCCTCCAAGCCCGGATAGATCAATGACCAAGTTGTGGAGACGGCTCAAGTCCAAAGGTGACAACCCTTGCGGCAGATCATTTCACCGAGCTTGTGTGGATCCTTCAGGTCGTAATTTATACATCTTCGGTGGCATGGTAGATGGTTTACTTAATCCTGCTGAATCCTCTGTGTTGAGGTTTGACGGGGAGTTGTTTCTTGTGGAGCTTTTGCTTCAGTGCTAG

>StKFB28

ATGGCGGATCAAAACAACAATGAAGACACAGTTATAAACTGCAATTTGCCCAAAGATGCAGTGGAGCGGATACTTGTAGGGCTTCCGGTCAGATCGCTGCTCCAGTTCAAGTCTGTATGCAAATCATGGTACAATTTCATCAAGAGCCGTAATTTTATCAAAATTTACCGCAATCATCAAATCTCTCGTCCTCCTTCTGCCCTTATATCTTACAAAAAAAATAATGTTAGCTCATTCTGGCCTGATTTCTTTTCGCTCAATCTTATATCCAACATTGAACAGTATAATTTCCCTATAAACCAGCAATTGTTTTTACCTTTTGCTATTGATGACGCAAAGGACTGGGCGAATTTTGAGTTTTGCTACAATGGCTTAATTTGTTTGACGAAGGATATCTATACCTCACCAGTAGTTATTCTATGGAATCCCGCTAGTCGGAGATACAAATGCATTAATATACCCTCTGAGTGCTCCAGATTGACTTATCTAAAACTCGGGTTTCATCAACAAAGCAATGATTATAAAATTTTGAAAGTCCCCTTTCAAACGCGGAGTGATGATGAATCAACAAAAATGGCTTGGGTTTACACACTGAGTTCAGGTTCATGGAAAACCGTACCGTTTAGTCTTCTTAGCCTAACTGTTGCTTGGGGACCTCAGATTTCTGTAGATGGTTTTGTGTATTGGCTGGGTACACGCGGGGTGGAATATATTATCAGCTTTGATCTGATTAAAGACGAATTCAAACTTATGGATGTTCTTGATGATCGTGGTTTTGATCGCCAACTTGTTCGTAGAAAGCTTATGGTTTTGAGGGGATCACTTGCCATGATAGTTTCTGTTGAGGATTGTACTAGGGATACTGAAATATGGATGCTTGTGAAAGGAAATAGTCGCCTTCAACCATACTCATGGACTAAGAAGTTCATTTTAGAACCTTTTTCCAAAGAAACAACCACGTTGGGAATGTGGAACGATCATAAGCTTCTCGTTGTTGTTTCACAAACTCGTCCTCATGCTCTCAGACAAGTCTACTCTTATGATCTGGTTACCAAAGAGATGGAATATTTTCATATGCCAAAGGAACAAGATTTAGCATCTTTTGCGGCAGTGCGTGGGTGCTATTTTGAGAGCTTGGAACTTGAAGATGGATCTGGCCGGAGATACTGA

>StKFB29

ATGGACCTGATCCCGGGGATACCTAATGAAATAGCTCTTGAATGTCTCATACGTCTACCTGTTGATCAATTTTCTAAAGCAGCTTCAGTATGCAAAAAATGGAACGGCGAGATCACGTTGCCGGAGTTTCGCCGCCGGCGGAAAGTTTCTGGGTTGACCCGAACCGTTTTCGCCATGGTACAATCTATGGTTGCTACTGTGAAAAAACCAGAGGGTGATACTGCACTTTTTTCTACTCAGGTTTACCGGCTCTCTGTATGTGACCCGGAAAACGGCTCCTGGTATGATTTGCCGCCGATACCGGAGTTGATTGATGGATTGCCGAGGTTTTGCCGGATTGTTGGAGTCGGGTCGGATTTAGTAGTGATTGGCGGGTGTGATCCGGATACTTGGCGGGTTATGGATTGTGTTTTCGTTTACAATTTCATATCCGGGTCATGGCGTCGTGGGGCGGATATGCCAGGTCAGCAGAGGTCATTCTTCGGATGTGCATCGGATTCCAATAGAATGATCGTCGTCGCCGGTGGCCATGACGACGAAAAGAATGCGATGAAATCGGCGCTCTCATACGACGTCGTTAAAGACGAGTGGATTACACTTCCTGACATGGTCATGGAGAGAGACGAATGTAAGGTTGTATTTCACAAAGGTAAATTCCACGTCATCGGCGGTTATCCTACGTGGGCGCAAGGTCACTTTGAGAATGATGCTGAGGTGTTCGACACCGCCACGTGGCAGTGGCAACTGGAAGATGACTTCTTGAGTGTGATCAACACTTCTCCTCATAGTTGCATTGAAGGTGATGATGGGAAATTATACATGTGCCGAGGCGGTGACGTGGCAGTGAAGGAAGATCCTACGTGGCGGAAGTTGACGAGGTTGCCAGCAGGGATTACTAGCGTGGCATATTTGACATCGTGTCGGGGGTTGCCAGCAGGGATTACCAGCGTGGCATATTTGACAGCGTGTCAAGGGAAGTTGATATTGGTGGGAAGGGGCCAATTTGATGAACTATATAGTATTTATGCTTTGGATTTGGATGTGGAGAGTGATGGCAAAGTGAAAAAATGGACAAAAGTAGAAACTTCTGATGAATATAGTGGTCATGTTCAATATGGTTGTTGTTTGGAAATATGA

>StKFB30

ATGCAGATCCTTCAATGGCTTTTCAAGAATGCAAATGAGACCAATTTTAACACCAATTCTAGCAAGAAGCAAGTTGTTACTGATGAGGAAGTGAAATCCCAAGGCATTGTCCTGTTTGCGATTTCTCGAAAAAAGAGAAATCGAGCTGGAAAATCAGCAAATTCATGCTGCTGCAACATCCTCAATTTGTTCAACACATTACGCAGAAAAGACATTGCGAAAGATTATTTCTACAGCACGATTCGTCTAAAGAGATTGGAAAGTTCGCGAAGGAAACAACAATTTGTAGATTGCATGAAGATGAAAAAACAGAGTCTTACTCGTGGATTAAGCTTTCGTCGAAAGTCAGATTCTGCCACAGCCAAAGTTTTGCCAGTTAGTGATGGCCATAAGGAGCAAAATTTTAGTGCAGATAAGAAGGAAAAGGCTAATGGAGATAAAATCAAGACAGTTTCAAAGATGAAAGAACTAATAAGATGGGCTGCTGCTGCTAAATCACAAAAGGGAGGAAAATACTTTGGTCAAAAGGTTCTGCACTTACGCGATAGGTCGGACTTAAAGGCAGTACCAGATGATGATGATCAACTGAGCTATGACTCTCCAAAGATCAGCTTCAGATGGGATGTTGATCAAAATTACTCTGCAATTTCAGTGACAAAAAATCACCAATCTATAAAAAATTCCCCTTTAAACACTATGGTCCATATAGATCAGTGCCTTGCAGCAACTAGAAAAGGGAATTGGGTCACCACAGATTCTGAATATCTTACTGAAAAAATCCTCTCATACCTACCAATTCGTTCTATAATTGTTGCTTCCTCTGTATGCAAGATCTGGAATTCCATCATCACTTCTTCTTCTTTCACTACCAAAATCTCTGCTACAAAAAAGCAGCCATGGCTTTTTCTCTGTGGCCAAAACAGCATTTTTTACAACAATAACCAACCATTTGCTTATGACCCAGATGCCAATGAGTGGATTTCACTTCCCACTTCAACCCTTTTGTCCCAAGATTTCTTTATTGGTTCAAATGGTTTTTTCTTTGCTACAACTTCTGAAAATTTCAGTTTTAAGCCTATTTTTAATGGAATTTGGGTCCAAACAAGCCCTTTGAGGTTCTCCAGGTGTAATCCTCTCGTGGGTGTTTACAATGAAGGGTTAAATCCAAGATTCATTGTTGTTGGTGGGGTTAGATTTGTTGGTGGATTAGTTGATGTTGAGGACAGATTGGCTGTTGAGATTTATAACCCAAATCTTGATTATTGGGAACTGTGCCCACCTTTACCAGCAGATTTTAGGTCAGGGAATTCATCACAGTGGTTATGTTCAGCTTTGTTAAGAGATAAATTCTACTATGTTTTTGGGATATATTCTTGTTTTATTACATGTTTCAATTTGGATGAACATTTGTGGAGTGAGGTACAAACACTTAGACCACCTGGGATTTTGTTTTCATTCTTGGTAACTTGTCAAGATTGTTTAGTTTTAGGTGGATTATGCAATTCACCAAATGGACCAAATTTTGTTCTATGGAAGGTTGATGAGAAAACAATGGAGTTTAGTGAACTTGCTACAATGCCTCAAGCATTGATGTATTGTTTGTTTGATAGTGATGAAGATGACAAATTTGCTAGTTTGAAATGTGTTGGATTGGGGAATCTTGTTTATGTGTACAATGAAGAACATCTTAAGAATTATCCTGCTTGTGTTTGTGAATTTAGTAATGATTTTGGGATGTGTAGTTGGAGGAAGTTGCCTAATTTGCCACCAAATGCTAGTAAGTTTCATAGAGTGATTAGCTTTTGTTCAAATGTTTCACTTGACAGCATTCTTGTTGGTGCAAGAATTTGA

>StKFB31

ATGTTGGACTTAAACAAAATAGAACCTGAAGAAGGAAAGCTAGACTTGGATCTTCGTCGCGTTAACTATGATCTTGATGACAAATCTCATCTTAGATATGGTGATTGGGTCACTTTCAAGAGCCATTCATCTAAAAAAGTTAGAATCTCTCGACCTGAATCTCCGAGAGGTGAAAATTCAGATAGACAACCTCAGGATGCAGATTATCCCTCACTTAGCTATGAACTCGAAAGTGAGATTCTATCCAGGTTTCCTAGGTGGGAGTATTGGAAACTTAGCCTCGTAAACAAGAGATGTTCAATGCTTCTAAAGAGTGGTGAGATATTTGAGATTCGTAAAGAGAATGGATTTAAAGAACCTTCGGTATATATGTTAGCAAGTGGGGAGACTAATTGGTGGACGTTTGATAGAGAGTTTAATTCCCGGAGGAAAATCCCTGATTTACCATCAGATGTGTGCTTCACATTTGGCGATAAGGAATCGCTATGTGCAGGCACACATTTACTTGTTTCGGGTAGGGAAATCGATGGTCTTGTTATATGGAGGTTCGAATTAGCAACGAATTGTTGGTACAAAGGTCCTTCTATGGTTAATCCAAGATGTTTATTTGCATCAGCTACTTGTGGCACCTCTGCTTTTGTTGCTGGTGGTGTTGGTATTATGGCGAACAGTGAGGTCTATGACACAGCTGAGAAATACAACCCCGATAGCAGATCGTGGGACCCACTACCGAGGATGAAGAGGAAGAGGAAGCTTTGTTCAGGATGTTACATGGACAAGAAATTCTACGTGATCGGGGGGAGGAATGAGAACGGAGAGCTTACCTGTGGAGAATTCTTCGATGAGGGTAAGAACAAGTGGGAGCTGATTCCGGACATGTTGAAGGATGATCCAGTCCTAACATGCCACTCACCACCCCTCATTGCTGTCGTGAACAATGAGCTGTACTCACTCGAGGCATCTTCAAACCAGCTGAAGCTTTACCTGAAAAAAACCAACACTTGGAAAATGTTAGGACAAGTGCCAGTTAGAGCTGATTCAAACAGAGGATGGGGCATTGCTTTCAAGTCATTGGGAAATGAACTTCTTGTAATCGGAGCTGCTTCATCGTCAGCCTCTTATTCGGGTAATTCCATGGCTATATATACTTGCTGTCCAGATCCTGATGCAATTGAGTTGCAGTGGCAACCTCTTGACAGTGGTCGAAATCGACTTAGTAGCTTTATCTTAAACTGTTCTGTTATGGTAGCTTGA

>StKFB32

ATGCCTAATGATATCACCTTTAACATTCTTCTAAAACTTAATGTCAAATCGCTTTTACGATTCAAATGCGTTTGCAAATCATGGTGCTCTTTAATTGAAGATCCCCAATTCATCAAGCAGCACTACGATATGTCTAAAAAGGATGTGAATCGTCATAAATTCTTCTTGACTGGTGGTGAATGGGAAAACAAGGACGACTATTTCTTCTCTATAGACACTCCACTTCAACATGATTCTGCTGCCTCTCTTATCGAGTCTCTGATTCCAGGAATCAATCATTTGAGTAGCGTAAGTTTTGTTAGTTCTTCTAATAATGGTATAATTCTTATGGTTTTCCCTTATGATTTTATCATTTTGTGGAACCCGGCCATCAGGGAATTAAGAAAAATTCCAAGCTCAATCCCTAAAATTAAAAAGTCAGGTAGAAAGCGGTTTCCTGCTATTTATGGTTTGGGCTATGTTTCGTCTATTGATGATTATAAAATAGTTAGAGTAGGGGGAAAAGATAACTCAAATGGTCATTTTGAGATCGATCTCTTTTCGACGAGAGATAACTCGTGGAAATTGATTGGCAAGTTTCCTCCGTATAACTTTTTCCGTGAAGGAGATATTGTTAGCATAGATGGTATCGTTTATATGATAGTGATGACAAATTTATCTGAAAGTGTGAATGAATCTACTATATTAAGTTTTTGCTTGGAGAAGGAACAATTTGAAGAAGTATTATTTCCAGATCGGATTCTAAGGTTCCAAAATCCAATTTTGCATGTTTTAGAAGAAAATCTTTGTTTGACTAGAATGCATGATTTAGCAAGTCGTGATTTTGAAGTTTGGCATATGAAAAAAGATGGATCAATGAGCACCACATGGAGTAAGATCTTGACGATTCCATCGATGTATTTATGGACGTTGCTTGAGGCGCGTGAAATTAATGAAAATTAA

>StKFB33

ATGAGTGATGAAACTGCTGATTGCTCTCTGACCGGAGATGTAATCTACGAAATCCTTTTACGGCTTCCGGTGAAGTCACTGCTCCGATTCCGATCGGTCTCCAAGTCATGGTATTGTTACATCAGCAGCCGCGATTTTATTCAGATGCACCGTCGACAACCTGTCCATGAAAAGCTTCTTAGAGTAAGCGATAGAGGTCCTAGAGACATCCCTACTATCTCCTTCTTCTCTCTAGATCCGAAAATTACGACTGTTGTTGTTGATGATGAAATTGACGAACTCGGAGATTTCGATTCCAGTCCCGATCCGTCTGTTGTCGTTGTTGATTTGCCCTTTCCATGTACTCCCGACGATGAGGTACGGGTCGTTGGTTCTTGTAATGGCTTATTATGTGTTCATTTCAATAGGAGTTCAAGCATTATTTTGTGGAATCCTGCAACTAGAAAGTATAAATTGCTTGAATCCCCTGATTGTGTCTCCTTTTATACTGATCCTTTCTTCCCCTGTATAATGCTTGGTTTCGTCCCTCAAACTAATGATTACAAGGTTATTAAGCTTCCATCATCTAGTAAGAACCCTAAGGTTTGGGTTTACTCCCTGAATTCCGATTCCTGGGAAGAAATTGAAGCTCCTATTCTTCATGGTTTGCTTCCCAAAGGTGGGTCTGCAGTAATTCTTAATCACTGCTTGTATTGGTTATCGTATAGCAATGATGACGATAATGAACTCGATATAATTACTTGTTTTGGTTTGTATAACCAAGTTTTTACAAGGATTAAGCTACCGAATCTCGATACTGCCTCTAACCTCACTATTCAGAAGCTTGTGATACTTAAGGGTTGTCTTTCTATGATTACCTATTCTGGGAATGGTATAGCTGTGAACAACTATGAGGTATGGGTGATGTCTGAACAACAAGGTGCAGCTGAGTTTTGGACTAAGCAATTTGCTTTTTCGTCGTTCTCTAATCTAGCACGACCCGTTGGATCATGGAGGAAAGGTGAACTTCTTTTAGGCTACCCAAATGGATTGTCACTTGAACTCATATCATACAATCCTTATACAAAAAGAACTCATAGCTTTCAAAAGAGAACTTCAGAGGATAGATTTAAAGTAGTAAATTATGTCGAAAGCCTAGAATCCGTGGAGGGTAGTTAA

>StKFB34

ATGGCTTCTAGGAGGAATTTAAAGAGAAAATCAATTGAAGATGAAGATCTTGTTTTTCCATTCGATGAACTTAATCAAGATCTTCTTGAAAGGGTTCTTTCATGGCTTCCAACTTCAACTTTTCTAAGATTAAGTTCAGTCAGCAAGAGGTGGAAATCTGCTGCTAATTCCCCTGTTTTCCACCATGCTTGCTCTGAAATTCCATCAAGAGAACCTTGGTTCTACATGGTTGATTCTTCACAATCAGTCGAATTCAAGAATCAACAACACTTTGTTTATGATTCTGCTGAAATGAACTGGAAATTCCTCACCTACCCATCTAATTTTCTTGAAGAAAATCAGCTTAATCAGTCAAATTTCCTCCCTGTTGCTGCTTCTGGTGGACTCCTCTGTTTTCATAACGGCGAAAATGGTGAATTCCTCATTTGTAACCCTGTTACTTCCTCGTATCGCAAGCTTCCTTTATTGGATTATTCCGATACCCTTTCTGCCATTGGAATGATTTCTACTCAGGAATCGTATAAACTCTTCTTAATTTTCGGAGAAATTCCGAATCTTTCATTCAGAATCTATGATTCCACTACAGATAGCTGGAAAGACTCTGCAATTCTGAGTAAGAAATCTCCTACTTGCCCTGCAGCACAATCCTGCAGTACTGACGATGAAGAAGAAGAAGACGATGATCGAATGCTCTATTTCTTGAGCAAATGTGGGAATGTAGTAGGAACTGAAATACAAAAAAACCCATGTAAGCAATACTCTTCAATAACTACCACCACGAAAGGCGATGGACAAGAAATCCTCTGTTTCTTGAACTCTTCCGGGAAAGTGGTGGCTTGCAATCTTACTGAAAAACACTTTTTCGAGTACCCAAGATTGCTCCCTCTTCATCACGAGTACTCAATCGACTTAGTTGAATGTGGAGGAGAGCTATTAGCAGTTGTTCTATCTGAATTCCTTGAAACAGCTAGTCTCAGAATATGGAAATTTGACGAAAATGAATGGGCATGGAATCAGGTGTTGGCAATGCCATCAGCAATTTCACATGAGTTTTATAGCAAGAAAGTCGACATCAACTGCACCGGAAGTAGTGAACACATGTTTGTCTGCATTTCGAATTCTGGTTCGACTGATGATGAGAGTTGTAGGTATTTCTTGTGTAAATTGGCTGAAAATGAGTGGACTGAATTGCCTGCTTGTTCTGGGAAATTCAGTTGTGCTTTCTCTTTTCAGCCTAGGATTGAAGCTTCTGTGTGA

>StKFB35

ATGAACAACTCCGCCACCATCACTGTCGCAGCCGCAGATACAGCAGCCGGAGCTGCTGTAAAACGCCGGAAAAAACTTCATCAACATCATCAGTATGAGTCCTTGATTCTTGGACTTCCTGATGATATAGCTCAGATTTGTCTTTCTCATGTTCAACCTTCAACTCTTTACTCCGTTTGTCAATCATGGCGTCGGCTTGTTTATTCTCCTTCGTTTCCCCCCTTTTTATCCATATATTCTCTACTGAAGCCATCTAAATCTGAGGAGAACTCCGTTCAGTTCGCGAATTTCGATCCGATTTCAGCAAAATGGAATTTACTTCCTCCGCCTCCACTTAATCCACCTCTCCGTCTCCTTCTCCGTCACCGTTCGTTCATCTCCCGACACCTTCCGATTCAATCGGTAAGCGTTTCCGGCAATTTTATACTCCTCGCCGCGACGACTGACCCTTTGCTCCCGGCCTTATCTCGTCCTCTTGTTTTCAACCCGCTTACTCGTAAATGGACCTCCGGTCCCCGGTTCAAAACTCCGAGACGATGGTGCGTTGCGGGCGCGTCGCAGGGCGTGGTGTTTGTTGCGAGTGGGATTGGGTCAAGCTATAACCCAGAGGTAGCTCGATCAGTTGGAAAGTGGGACCTGAAAAGTAACTGCACAAAGTACAGCTGTAACCATCACGATAGAAACAAGGTCTGGAAATGGGAGAAAATGAGTGGTCTCAAGGATGGGAAGTTCAGTAGGGAAGCGATTGAAGCAGTAGGCTGGAGAGGGAAGCTCTGTATGGTAAACGTGAAAGGCGATGCAGCGAAAGAAGGAATAATCTACGACGTCGGAAGTGACACGTGGCAGGAGATGCCGGAGGGGATGTTGGTTGGATGGAGAGGTCCGGTGGCGGCGATGGAGGAGGAAGTTATTTATACGGTGGATGAATCGAAAGGAGCATTGAGAAAGTATGATCCAGGAAGTGATAGTTGGATTATGATTTTGGAAAATGAGATGCTTAAAGGTGCACAACATATGGCTGCTGCCGGCGGTAGAGTTTGCGTGGTTTGTGGCGGCGGTGATGGGATAGCGGTGGTGGATGTGACGGCTGAGCCGCCGAGTTTGGTGGTGGTGGAAACTCCGGTGGGGTTTCAGGTTTTGGACGTACATATTTTGCCAAGGATGAATCAATTGGATTCTTAA

>StKFB36

ATGTCTGTACTCATTGAAGGGCTTCCTGATGCTGTTGCTCTCAGGTGTCTTGCACGGGTTCCATTTTATCTTCATCCCAAGTTAGAACTTGTTTCCCATTCCTGGCAAGCTGCTATTCGAAGTGGTGAACTATTTAAGGCAAGACGGGAGGTCAATTCATCGGAAGAATTTTTATGTGTCTGCGCCTTTGATCCTGATAATTTATGGCAGCTTTATGATCCTATGCGTGATCTTTGGATTACTCTCCCTGTTCTTCCGTCAAACATCAGACATCTTGCACACTTCGGTGTGGTATCTACTGCAGGAAAACTCTTTGTTCTTGGTGGTGGTAGTGATGCTGTGGATCCATTGACTGGTGACCAAGATGGAAGTTTTGCCACGGATGAGGTCTGGTCATATGACCCAGTCACCCGAGAATGGAGTCTCTGTGCGTCTATGATTGTGCCTCGAGCCATGTTCTCTTGTTGTGTGTTTGATGGGAAGATAGTTGTTGCAGGGGGTTTCACTAACTGCAGAAAATCAATATGTAAAGCAGAAATCTATGATCCCGAGAAGAATGTTTGGGATCCGATCCCTGATCTTCATCACACACACAACTCTGCCTGCTCAGGAGTGGTTATTGGTGGTAAAGTTCATGTATTGCACAAAGGTTTGTCAACTGTTCAGGTTTTGGAAAATGTGAAGCAGGGTTGGACTGTGCACGAGTACGGTTGGCTCCAAGGTCCCATGGCTGTTGTTAGGGGAGAGCTTTATGTATTGAGTCATTGGCTCATATACAGGCAGGAAAGAGAAACAAGGAAGATGGTAGTTTCAGCATCAGAGTTCCGTAGAAGAATTGGGTTTGCAATGATAGGTCTGGGAGATGATATCTATATTGTTGGAGGGGTTATTGGACCCGAGCGCTGGAATTGGGACATTAAATTGCTGTCTGATGTTGATGTCCTGACACTCGGAAATGAGAGGCAAGTGTGGCGTCAAGTTGCTCCAATGACAAGGTGTAGAGGAACGGTCCTTGGCTGCACGCTAATGAGAATATAG

>StKFB37

ATGGCTCTTCCTTCTTCAACAACTGTCACAAATCAAGAATCTCAACTACAAACCCTAAACCCAACTATAATCCCTGGTCTTCCAAACGATTTAGCAGCGGTTATACTCGTATTCGTTCCTTACTCTCATCATTCACGTCTCAAATCCATTTGTAAATCATGGAAGCAATTTTTCTCTTCCAAAACCATCATTTCTCTGCGGCAAAAGCATCTCCCACTATCTACTCTGTCTCCCCTTCTCTGTATATTCCCACAAGATCCTTTAATTGCTTCGCCGTACCTGTTTGACCCTAGAAATCTTGCGTGGTCTCCACTTCCACCTATGCCTTGTAACCCTCATGTATATGGGCTTTGTAATTTCACTTCGATTTGTATCGGCTCTCATTTGTATGTGTTAGGTGGGTCGCTTTTTGATACACGATCTTACCCTCTTGATTACCCATGTCCTTCATCCTCTGCTTTTAGGTTTGATTTTGGAACTTCTTCATGGGAGACGTTGGCGCCTATGATTAACCCTAGGGGGAGTTTTGCGTGTGCTGCGGCACCTAATTTGGATAAGATTTTGGTGGCGGGTGGTGGGTCTCGGCATACGATGTTTGGGGCGGCTGGTAGTAGGATGAGTTCAGTGGAGATGTATGATATTGGGAAAGATGAGTGGGTTGCATTGGATGGGTTACCTAGGTTTAGAGCAGGGTGTGTTGGGTTCTTTGTTGGGAATGGAGAGGAGAGGGAATTTTGGGTGATGGGTGGATATGGTGAGTCGAGGACGGTCTCCGGTGTGTTTCCTGTGGATCAATATTATAGGGATGTTTTAGTGATGGAAATGAAGAATGGTGGGAAGTGGAGGGAGCTTGGGGATATGTGGGAAGAAGGGGAAAGGTGGAAGCTTGGAAAGATTGTGGTGGTTGAGGATGTGCCTTCGGAAGCTCCTGCAGTATTCATGCTCGACCGAGGAGATATTTTCAGCTATATAATGGCTTCCAACAGTTGGATAAAGGAAACAAGTCTGCCAAGAAAAACATCTGATGAATCATCTGTTGGCTTTGTTGCATTGGATGGGGAGCTGCATGTGATATCTCATTTAAATGGGGTTAAATCAAAGGAGTGCCAAAGATTAAGGCAGCAGAAAAGGTCAACTATGCTTGTACAAATATACCATCCTAGGACGAAGTCATGGAGGGCTGTCACTACAAGGTCACCTTTTCAGCACCCTTTGGATTTCAAAACTGCAGTTATGTGCACCATTCGTCTGTAG

>StKFB38

ATGCCTAATGATATCATCCTTAACATTCTTCTAAGACTTCCTATCAAATCGCTTTTACGATTCAAATGCGTTTGCAAATCATGGCGCTCTTTAATTGAAGATCCTCAATTCATTAAGCATCACTATGATATGTCTAAAAGTGATGTGAATCGTCATAAAATATTTCTAACAGGAGGTATATGTGACAACATGGACAACTATTTCTACTCTGTAGATGCTCCACTTCAATATGATTCTGTTGTCTCTGTTCTCGAGACTCCGATACCTGGAATCAATACCTTAAGTAGAGTAAGTTTTATTAGTTCTTCCAGTAATGGTATAATTCTTATAGTTTTTCCTTATGATTTAATCATTTTGTCGAACCCAGCCACCGGAGAATCAAGAAAAATTCCTAGCCCAATTCCTAAAAAGAAAAAGTTAGAGAGAAGACAATTTCCTGCTATTTATGGTTTTGGATATGTTTCATCTATCGATGATTATAAAATAGTTAGGGTAGGGGGAAAAGATAACTCACATGGACATTTTGAGATCGAATTGTTTTCGACGAAAAGTAACGCTTGGAAATTGCTTGGCAAGTTTCCTCCAAACAGCTTTTACTTCGAAGGGGGCATTGTTACCATCGATGGTATCGTTTATATGATAGAGATGGCGGATTTATCTAGAAATATTAATAGATCTACTATATTAAGTTTTTGCTTGGAAAACGAACAATTTCAGGATGTTTTGTTTCCAGATCAAATTCAAAAGTTCCAAGATCCTATTTTGTATGTTTTAGGAGAAAATATTTGTTTGACTAGGATGCACGATCTAGCTAGACGAGATTTTGAAGTTTGGCATATGATAAAAGATGGATCAATGAACAACATATGGAGTAAGATCTTGACGATTCCATCGATGCATTGTGGACGTTGCTTGAGGCCCGTGAGTTTAATGAAAATTAATGGATACATAATGCTTCTGAAGCATAAGGGTGATTTCGAGGTCTATAACTCAAATGGAGAGCAAATCGAAATAGTTGAAGTTCCTGGGCTTGAGTCATCACTTTTCTTCAATTGTATTGTACCGTATGTAGAAAGTTTGTTTACTCCTAAACGGAAACGGATTTAA

>StKFB39

ATGAATCAAAATGAGTTCACTGAATTAGTACCGGGTCTTCCCGAGGACATTGCCCTCGAGTGTTTGACCCGGTTACACTACTCAACTCATGGAGTTGCATCTCGTGTTTGCCGAAGATGGTGTAGAATTCTTCAAAGCAAAGCTTTCTATTACCATCGGAAACAAACGGGTTTTACCCATAAAGCTGCTTGTTTAGTACAAGCTTTACCTTCTCCGGTTGAATCCAAACCCACTGGACAACCCAGGTACGCGATCTCAGTTTTCGATTTAGTATCCGGGATTTGGGATCGGGTTGACCCGATTCCTAAATACCCTGATGGGTTACCGATGTTTTGTCAAATTGCAACTACTGAAGGAAAGCTAATTGTTATGGGTGGATGGAATCCGGCGAGCTGGAATCCAATTAAGGACGTGTTTGTTTACGATTTCATGACTCGGAGATGGAATCAGTGTAAGGATATGCCGGAAGCTCGGTCGTTTTTCGCTATGGGAGCTGCCGGAGGAAAGGTTTTCATCGCCGGCGGTCACGATGAGAGTAAGAACGCGCTGAGTTCTGCTTGGGTTTTTGATATTTCATCCAACGAGTGGACGGAGTTGCCTAGGATGAGTGAGGAGCGAGATGAGTGTGAAGGGGTTATAATCGGCTCGGATTTCTGTGTCGTAAGTGGTTACGACACGGAAACCCAAGGCCGATTTAAAAGTAGTGCTGAGTTATATGAACTCAGCACGGGTCAGTGGAGACGAGTTGAGGATGCATGGAGGTCGAGTCAGTGTCCTAGGGCATGTGTAGGGGTGGGAAAAAATGGAAATTTAACTTGTTGGGCAGAATCCGACCCGAATGTTAAAGTCGGAGCATGTGGTGTGGATCTTGGTGATCGGACCTTGGTAACCGGGTCGGCCTATCAAGGTGCCCCACATGGGTTCTTTTTCGTAGAAAACAACAAAAAAGGACAAAACAGTAAATTGACAAAGATAAATGTTCCTGATGAATTTTCAGGATTTGTACAATCTGGATGCTGTGTGGAGATCTGA

>StKFB40

ATGAAACGAGGTCGTCGATGCAAATTGTTTGTTTGTGAAGAAATCATAATCAACATACTGAATCGTTTGCCTTTAAAATCGCTAGAAAAGTTTAAATGCATATCGAAGAATTGGCGAAAGTATATTGCTGAAATTTACAGGCGCCGTTTCCAGTGGCCTGAACCCTATCTACTCGGTTTCTTTTGCTTAGAGAAACGTTCCCAAAGTTGTTTCTTCTATTCATCAAAGGAATCACCACTTGTAATCGGTTCTAGTTTGGATGAATCAGTCAATTTTATTGGTGAGAAAGTGTATATTGTTGCTTCTTCCAACGGTTTTCTACTATGCAATAAGCTTAGAAGTAGGCATAGGGTTTATTATGTTTATAATCCTGCCACAAGGCAGCGTTTGGATGTCCCTAAAACTCCAATCTCTATGGATGATCCATACATCGGATTTACTTGTAAGGTAGGTGAGGATCATGGCATTGTCTCGTTTACTATAGTTCGTTATGTAATTAGCGAACACTATCAGAGTAGTATAACAATTGAAAGTTTCTCTTCAGAGATTAATGTGTGGACTGATTATAAGCTAATTGTTGATGTACCTCATGCATTGTATCCTTCTTTGGATGGGACTTCGTTATCATCTGCTGGTGTAGTTGATGGAATCTTCTTTTGGCTTAATTATACATTGATCACTATTTATGATAGTGTAAACAAGTGTTTTTGGGCTTTGGAATTACCTGAAAACTTGTTTATCTATTATCCCCGTAGTTGTTGTCTTGGATTATCAGGCGGGGAGCTCTGTTTTGCATCGAATCGTTGGACAACAACCATCACTTGTTGGCGACTCAACAATTTTCCTAGTCGAGATAATGCTGAATGGGTTATAAAGTATGTTATAAATGTTGCTACTATAATTAAAAAATATCCCAAGTATTTTGGAGTTGGAGACGGTTTTGCTAAGGTTCAGAACATGGTTTTTCATCCTGCTCTTCCACACATCTTGTATTTGCAAATAAAAGACAAGGTTATTTCTTATGATGTGAAAACTCGTAAGACAGAACTTGTGTATGATTTTGGAGAAGCTTGGCGGAAGACAAAATGCTACAAATTGTTTTCCTATGAGTGGCCTCAATGGCCGCGTCTTCTGTAG

>StKFB41

ATGAAACATCTTCGTCAATGCAATTTGTTTATTCATGAGGAAATCATAATCAACATACTGAATCGTTTGCCTTTGAAATCGCTAGCTAGGTTTAAATGTATATCCTATAATTGGCAAAAGTGCATTGCTGAAATTTATCGATGTCGTCTCGGTTTTCCTGAACCTTACTTGATCGGTTTCTTTTGTGTAGAGAAACGTTTGCGAAGTCGTTTCTTCTTCACATCGAAAGAATCGCCACTATTAATTGGTACTAATTTGGACAAATCAATCGATTTCATCGGTGAGAGAGTGTATATTGTTGCTTCTTCCAATGGTTTTGTACTATGCAATAGGCTTAGAAGTAGGCATAGGGTTTATTATGTTTATAATCCTGCCACAAGGCAGCGTTTGGATGTCCCTAAAACTCTAATTCATATGAAAGATCCTTATGTTGGATTTACTTGTAAAGTAGATGAAGATCATGGCATTGTCTCGTTTACTATAGTTCGTTATGTAATTAGGAAACGCTATCAGTCTAGTATAACAATTGAAAGTTATTCTTCGGAGACTAATGTGTGGATTGCTAATAATGTCATTCATGATTTACTTTATCCATTGCAGCCTAGTAGGGATGAGAATTACTTATCATCACGTTGTGTAATTGATGGAGTATTCTGTTGGATTGATAATTTTGGACAAGGGATGACTGTTTATGATAGTGTAAAAAAGTGTTTTTGGGGTTTGACATATCCTCAACCAGGGTGTATGATCTATCCAGGTTTTCATTTTCTTGGTTTATCAGGTGGAGAAGTCTGTTTTGCATCGAAAGGTTGGACCATCCCTTGTTGGAGACTCAACAATTTTCCTAGTAGAGATGCAGAATGGGTTTGGAAGTATAATGTAGATGTTGTTGCTATAATTGAGAAATGTGGAGAGGATTTTGGACTTGGAGGAGGGAATGTTCAGAACATAGTTTTTCATCCTGTTTTTCCGGACATTTTGTACTTGCAAATAAATGGCAAGGTTATCTCTTATGACGTGAAAACGAGTACTGTAGAACTTGTCTATGATTTTGGAGAAGCTGGGCGGAAGACAAAACATTACAAATTGTTTTCCTATGAATGGCCTCAATGA

>StKFB42

ATGAAATCTTGTTTCCCCAATAACCAACACCCACCACCCCACCACCCTACCCCCATCGGAACCACCACCATTTTACCCACCGGAACCACCATTGCTGAACTCCCAGATGACCTTCTTCTTGAATGTCTATCAAGGGTTACTCATTCTTCACTTCCTTCTTTACCTTTAGTCTGTTGTCGTTGGTCTCTGCTTCTTGATTCCCCCACTTTTCATCTTCTCCGACACCGGCATAACCTTTTACGGCTTACCCTTTTCGCTGTTTCGGTTTCTGATGGAGCCCTTTGCACAGCTAGTTACAGATTGAACAATGATTGTTCATGGAAGATTTGTTCTTTTACACCGGCGAATGACCCTGTTTTCGAACATGGGTGTTTTTATTCTTTGTTCTCGCATTCTCGTTTATCAGTTATTGGACGGAAAATCTACGTAATTGGACGGACGGCGATGCTCCGGTGTGATACCTGGACTGGTTTAGTAGTTCCAAGACAAGGACCGGTTTTCCCGAGGAAGAAATTTGCTGCGGCGGTTGTGGGTGGGAAAATCTACGTCGCCGGAGGTTGTGCGAGGTCTGCGGCGGTGGAGGAGTATGACCCAACGAGTAATACATGGAGTGTGGTGGCTAAAGCTCCGAGGAAGAGGTATGGTTGCGTTGGGGCTTCAGTCGACGGCGTTTTTTACGTCATCGGAGGATTGAAGCTCGGAGGCGCGTCGGGAAATGAAATGCTGGTGGCGCGTGGTAGCCGTGCATCTGATGCAGCTCATGTTTACGCTAGCTCAATGGATTTATACGATACTGTTAATGGGGTTTGGTTAAAAACCCGATCTGTTCCCGGCGGCGGCTGTGTTGTGGCAGCATTGGAACTTTCGTTCTGGAAGTTTAATGGGTCACGTAAAAGTACCGGATTCGGCGATTGGTGCAGGATAAAATCGCCGCCGTTGCCGGCGCAAGTCAGATTAGACAGTACGGTGAGATTCAGCTGCGTGGGGATCGGAGAAAAGGTGGTTTTAGTACAAGTAAATGGTTGTATAGACGATTTGTTGCGGCGGAGTGGGAGGATTGAGAGAGGATTGAAGGAGGGGTTGGTGTTGGTTTACGACTGTGTCGCCGGAGAATGGAGCCGTGCCGCCGATTTGCCGGAGGTCATTCGCCGATCAGCCTGCGTCTGTGTTGAGTGCTAA

>StKFB43

ATGGATTCAGTATTTGTATCAAACCCTAGAAACAATTATTCCGTTCCCCATGATATCATAATTGATATACTCACACAACTTCCTGTGAAATCACTTATAAGGTTCAAAGGCGTTTGCAAATCATGGTATTCGTTAATTAAAGATGACAACTTCATCAAGCAACACTATGACACTCACCAAAATTGTCAAAAGTACTTTGTGGTTTGTAGAAGACACAAGATTTCCACCCACTACACCATGGAACTTGATTCTAATAGTATAGCCTCTCTTGTCGCGCCTCCGGTTCCAATTGATCAATCACGAAAGGCATTCAATGAAATACAATATTGCTCATGTAATGGAATACTTCTTATAACATACGCTAATGACATAATCATTATGTGGAATCCGGCCACTAGAGAATCAAGAAGAATCCCTAAGTGTAAGAGTGGTGGTCTTTATAATTTTTGCTACTTTCCGCGTATTGAAAGCTACAAAATATTTAGACTAGGACCTGTAGTCTTTAATGGTGACAAGGATGAAATGGATATTGATATATTTTCAACGAAAAGTAACAAGTGGAAAACGGTTGGCATATTTCCTCCAGATTATTATTTCGAAGGTACTAGCATTGTTATGTCAGATGGGATTGTTTATATGATGGCGGAGAGGAAAGAAAATGAAAATTGTACAATATTACGTTTTTGTTTGGACAAGGAAGAATTTCAAGAGGAATTGTTGTTTCCGAACACGATACCAAGTGAAATACATGTTGTAGGAGAAAAACTATGTTTGATTGGGTCATTGATGAGTAATCATAAGATTCATGAGTTTTGGTTGTATATGATGAAAACAAATTCATGGAATAAGATATTGGCAATTCGATTACCTTCGAAATCTTGTTTGAAACCTTTGAGTTTTATAAAGGATGGAGGGATCATGTTTAAAAATTACATGAGTAAATATGTGTTCAAGGCTTACAATTCAACAACACACAAATTGGAAAAAGTTAATGTAGCTGGACTTGAAGGACATAACTTCAAAGAGGTTGTAACCTACGTCGAAACTTTATCCTCTCCGTTTCTTTAA

>StKFB44

ATGGATTCCACCCAAAACGTTCCCCATGATATCATAATCGATATACTCGCACAACTTCCTGTGAAAGCACTTATAAGGTTTAAAGGTGTTTGCAGATCATGGTATTCGTTAATTAAAGACAATAAATTCATCAAACAACACTACGATACTCACAAAAATTGCCAAAAGTACTTTATGACTTGTAGAAGACGCAATATGAAGAACCAATTCAATTACTACCACTACACCATGGACGTCCCTCAACTTGATTCTACTAGTACAATCTCTCTTGTCGAATCTCCGGTTCCAATTGATCGATCGCGAATGGTATTGGGTAACACACAATATTTCTCATGTAATGGAATACTTCTTATAATTACATATGCTAATGATATCGTGTTGTGGAACCCAGCCACTAAAGAATCAAGAAGAATCCCTTGTCCAATTCGGAGTAAGAGTGGTGGTCTTTATAATTTTTGCTACTTTCCGAGTATTGACAGCTACAAAATATTTAGACTAGGACGTGAAGTTTTTAATGATGACAAAAATGACATGGATATTGATATATTTTCAACGAAAGGTAACTCGTGGAAATCTGTTGGCATGTTTCCTTCAAATTATGATTTCTTAGATAGTAGCATTGTTATGTCAGATGGGATTGTTTATATGATGGCGAAGAGAATTGGAAATTTAGTCAGTAGTACAATATTACGTTTTTGTTTGGAGAAAGAACAATTTGAAGAGGAATTGCTATGTGTGGACACGATACCAGGGAGAATACGTATTTTGTATTCTGTAGGAGAAAAACTTTGTTTGATTAGTTCATCTGGAGATAATAATGAGGTTCGTGAGATTTGGTTGTATATGATGACAACAAATTCATGGAGTAAGATATTGACAATTCCGTTAACTTTGAGACCTTTGAGTTTTATGGAGGATGGAGGCATGATGTTTCAAAAGTTCAATAGATCTGGGTTCGTGTTCGAGGCTTACAATTCAACAACACACAAATTTGAACAAGTTAATGTAACTGAAATTGAAGGACTTAACTTATTCAATAGGGTTGTAACCTACGTTGAAACTTTATCCTCTCCTAATTCGTAA

**Protein sequences of 44 StKFBs**

>StKFB01

MEEEEENMRREKRLKCINMEEDEDEDEEEGEILDDDDDDDEYEEEIENIEVPSQPVGFFYPSTTPSSIVVSDALDPDLPVIYVNSAFESSTGYRADEVLGRNCRFLQFRDPRAQRRHPLVDPVVVSEIRRCLEEGVDFQGELLNFKKDGTPVVNRLRLAPIHSDDGTVTHIIGIQMFSETKIDLNTVSYPVFKETCQPHCDESSEYSIKSGNLLHREMCGILQLSDEVLAHNILSRLTPRDVASIGSVCRRIRQLTKNEHVRKMVCQNAWGADVTGVLEHMTKKLAWGRLARELTTLEAVCWKKLTVRGAVEPSRCNFSACAAGNRLVLFGGEGVNMQPMDDTFVLNLDAANPEWRRVSVKSSPPGRWGHTLSCLNGSWLVVFGGCGREGLLNDVFVLDLDAKQPTWKEVSGGTPPLPRSWHSSCTMEGSKLVVSGGCTDAGVLLSDTYLLDLANDKPTWREIPTTWAPPSRLGHSLSAYGKTKILMFGGLAKSGHLRLRSGESYTIDLEDERPQWRQLECGAFTGVGSQNAVVPPPRLDHVAVTMPCGRIIIFGGSIAGLHSPSQLFLLDPSEEKPLWRTLNVPGQPPKFAWGHSTCVVGGTRVLVLGGHTGEDWVLNELYELCLASKQDSDA

>StKFB02

MDQTIERSPNAHRGFRVQAPLVDSVSCYCKVDSGFKTVVGARKFVAGSKICIQPDINPHAHKTKNSRRERSRVQSPLLPGLPDDLAIACLIRVPRVEHNKLRLVCKRWYRLLAGNFFYSLRKSLGMAEEWVYVIKRDRDGRISWHAFDPTYQLWQPLPPLPGEYSEALGFGCAVLSGCHLYLFGGKDPIKGSMRRVIFYSARTNKWHRAPDMLRKRHFFGSCVINNCLYVAGGECEGIQRTLRSAEVYDPNRNRWTFITDMSTAMVPFIGVIYDGKWFLKGLGSHREVLSEAYNPETNGWSPVTDGMVAGWRNPSISMNGCLYALDCRDGCKLRVYDGASDSWNRFIDSKLHLGSSRALEAAALVPLNGKLCIIRNNMSISMVDVSSPDKQVETNPHLWENIAGKGHFRTLFTNLWSSIAGRGGLKSHIVHCQVLQA

>StKFB03

MDQTIERSSNAHRGFRVQPPLVDSVSCYCNVDSGLKTVAGARKFVPGSKLCIQSDISSHAHKSKNSRRERSRVQPPLLPSLPDDLAIACLVRVPRVELSKLRLVCKRWYRLLAGNFFYSQRKSLGMAEEWVYVVKRDRDGRISWHAFDPTYQLWQPLPPVPGDYSEALGFGCAVLSGCHLYLFGGKDPIKGSMRRVIFYNARTNRWHRAPDMLRKRHFFGSCVINNCLYVAGGECEGIQRTLRSAEVYDPNRKRWSFIADMSTAMVPFIGVVYDGKWFIKGLGSHREVLSEAYNPDTNAWSPVNNRMVAGWRNPSISMNGRLYALDCRDGCKLRVYDESTQSWIRFIDSKLHLGSSRALEAAALVPLNGKLCIIRNNMSISIIDVLSPDKRVETNPHLWENIAGKGHFRTMFTNLWSSIAGRAGLKSHIVHCQVLQV

>StKFB04

MAHESESVSLLPPEVISEILVRLPVKSLLMMRCVSKSWLSLISTRQFIKTHLEFSTNKQDFAHDILLLSSSSYEHTLRFYTCSLYAIMYQESPHVPDDLDFPCKDPLVEYNFVGSCDGLLCISGGARDLFLWNPSIRKSKKLPMSGSNVHCSSYLAYGVGYNECQDDYKVVEVMGSSHSEYGFQNEFRVYSLRTNSWKMIQEYPGVIFCNDPAKFVNGRLHWIATRVSDKNDSWFISSLNLVDETYENVGLPDLVYGNFDWELGILGGNLCVFCDYYKVQMDVWVMKAYGLVESWTKVASIPYFRAIEHSPFPVFISHNDEILLQHGSSLLIYNSTDNTFKHPQVQIHHGYEIQFSLYTKSLVSPHFVED

>StKFB05

MSKGEILPQDIIIDILSRLPAKSIGQYRCVSNQWYNFLSHPQFIKFHFTLHAHKQETKLIFISDSDDLHTITFNRNPQNIILDAISTNLNFQNNWLSIACSCNGLVLVENQEHIMYLINPTTLDYHRIPVFHLGLPQQSSYREYGFGYDFASDDYKVVNLSRYRKGNIDTTFVDVYSVRMGLWRRLESLPYDDVLSERGGASGVLVNGVLHWMASKASSFVIIGFDLSDEKFFEVPAPTNLYGNELDWYELRSFRGCLCMFCALLESEIDVWVMKEYRVEESWTTFRIDRMDLENGSVPFCPISDDDVVLSVDRDRLTVYNIKEDQWRYMEVDGLTYMFERTGIFIESLVLPMLGKGTEGYHIA

>StKFB06

MQSCKAAPSNPQGAGTNTEQVYSESENFVVTENSLDNDQSVDVEDSGSGDNSELDPEASRLIPGLPDDIALFCLARVPRRHHVLLKCVSRKWRDLVSGEEWYSYRKKHDLQESWIYALGRDKSEQLCCYVLDPTRLKRGWKPILGLPHRCIRRKGVGFEVLGKKLFLFGGCGWIEDATNEVYCYDAAMNKWNQATSLVVPRCYSVSEVLDEKIYAIGGIGPNSNNLPSWETYNTETTSWTLHENPNIFPDIEDSIVLGGKIYIRGGSSPLSSLVSAFVYERSSNTWQPAASELVSGWYGPAVVVDGTLYVLDQSSGTRLMMWQKDIREWVAVGRLSPLLTKPPCRLVAVGNNIFIIGKGLSTVVFNVENARNMDGVLVSTSIPKSISDDDVISCKAITI

>StKFB07

MEGETSWVSHCPDYVVPAMVEFDSFSELNDEENREASSVPVDLILPDDLLERILAYLPIASIFRASCVCKRWYEIVSSRRFLWNFSQVLSQKPWYFMFTSSEEPVGYAYDPSLRKWYSIDLPCIQTSNWFIASSCGLVCIMDNDSRSELYVCNPITKCSKNLQEPPGLKFSDYSALAICASMKTFCYSVAIVKSKQVPGNFYQWDLSIHIYDSGTMKWLTPLTEVLTGWRGGDESVICDGVLYFLIYATGGGGLESRHGLITYNLSSRSSHCSLIKTFIPVPCSLTCGRLMNLKEKLVMVGGIGKPDRPDIIKGIGIWVLKGTEWQEISRMPHKYFQGFGEFDDVFASSGTDDLIYIQSYGAPALLVFDVNQKQWRWSQKCPVTKRFPLQLFTGFCFEPRLEMSP

>StKFB08

MGGILSRSNHNSNVGDLIEGSQSASCKRQRTSDSFWEHSPRLIPSLPDEISIQILARLPRIHHLNAKLVSPSWKGAIMSPELYRCRKKLGTTEVWLYLLTKTEGDKFLWYAFDPISVRWQKLPPMPAIAVNDEPKSGLSGIRAWNMAGSSIRIADAIRGWLGRRNALDQVPFCGCAIGAVDGCLYALGGFFRAAAMRSVWRYDPIVNAWNEVSPMSTARAYCKTGVLNGKLYVVGGVTRDRGGLTPLQSAEVFNPHTGIWSEIPSMPFSKAQMLPTAFLADLLKPIATGMTSYRGKLYVPQSLYCWPFFVDVGGEVYDPETNAWIDMPLGMGDGWPARQAGTKLSVTVEGELYALDPSSTLDSARIKVYDHQDDTWKVIEGDIPINDNSESPYLLAGFLGKLHVITKDANHNIMVMQADRQNHSAPSPSTSANSSQKNLHEVPEPVLGSEANMWRVVALRSGGTAELVSCQILDV

>StKFB09

MQRVRVSSHQAPVQRLGDSQMTLSPKFRLAAKQSDLLDPSFDLEMWRKGEPLIPGLPDDVALNCLLRIPVDDHMNCRVVCKRWYSLFATKDRFFSRRKELGFHDPWLFVFAFHKTSGKIQWKVFDLKNSSWHTIPAMPCKEKVCPHGFRCICFPHDGVLYVCGGVASDVDCPLNLVVKYEVRRNRWTVMKKMITARSFFASGVIDGMIYVAGGNSTHLFELDSAEVLDPNKGIWCPVASMGTNMASYDSAVLNGKLLVTEGWFWPFYVVPRGQIYDPQTGNWENMASGLREGWTGSSVVLYGQLFVVSEHERTKLKVYDPETDSWDTVEGLPLPEQICKPFSVDCCDNRIVVVGRNLHVAVGHIKSLQPSSKRCSFAVYWQVVDAPASLSDFTPSSAQVLFA

>StKFB10

MDGLPHLPGDIVNSIFFKLPVKSLIRFKSCCKSWYGCIDDSDFIKSHLHKSSIDISRKKFVLVNSILLHREGTRKFKIVSTEASINADSKVVYLNIPEYFSDYFSLQVFSCSGLIFMTSYDLGYCMTLLNPVVGKYKLIQNSLFSQNTKTNRCSTSPIFGFAYDFVAEDYKVICAHYLINKYFNVVEVYSVKNQCWRAIHNTFPVSPDSYNQHLYSNQVSLNGVIHRMSYNRAVISFHLVDEKIYL

>StKFB11

MPPKGKGNGKKKGKSKGKGKGTSKEPKCRAAAEPEPTSHFYFPREIISNILSRLPVKTLLRFRCVSKQWRNLISKPDFIASHFRHSSSLQFSGSSILIGSRHRESNHHVVSLYNPPESVVQVDSPFPCFFPNMYIVGPCNGFICLFNPPWGELITLWNPAMRKYKMVELTDSLPRQGLHFLASIGMAFDFQHNDLLILRIFCVGIMYAVPNHVEMYSSKSGKWKKLKNEMIFHILEFTCNVIVKGVAYWLVCMPDKFGSRAVFVRFDVGKLVFEKLPSIGRRKKHQYLVELEGSLCMLDWDHKDDCHMDVWVMDDVDGWSKKYSVGPLVGFDLILGCLRNGDIVAKNENGVIFLCDPITSSIKEKFSFDNNKDGSYVIVDYSESLFLIGGMLPVKKQDAQDKLARKRVTRNSGNLAH

>StKFB12

MVEIAESSESGSGLLNSTQVKIGSLPEEDSIHWQVSSFGSSGSRNTSPLGRIGSRNTSPSRQKVVKTKPRGLDEETVTTFAKAVQPDVQMEDNIWAMLPEDLLNEILARVPPFMIFRLRSVCKRWNSILQDHSFLRFHSQVPSHGPCLLTFWKNSQTPQCSVFSLPLKQWFRIPFTFLPQWAFWLVGSSGGLVCFSGLDVLTFKTLVCNPLTQTWRTLPSMHYNQQRQLLMVVDRKDRSFKVIATSDIYGDRSLPTEVYDSKIDKWSLHQTMPAVNLCSSKMAFCDSRLYLETLSPLGLMMYRLDTGQWEHIPAKFPRSLLDGYLVAGTHKRLFLVGRIGLYSTLQSMRIWELDHTKVVWVEISRMPPRYFRALLRLSAERFECFGQDNLICFTSWNQGKGLLYDVDKKAWSWIAGCALQSYNSQVCFYEPRFDASIY

>StKFB13

MWSNLPFELLANIFSYLSPDSLARAKSTCKSWHTCANNSLSWATLPWRQYPPWFLALPTRNHGHFICAHNPIKDSWHLLPLDFIPNPIRPIAAVNGLILLRETTTTALQLAICNPFTRQFRNLPKLNVTRTNPAVGVISLNSANFQVYVAGGMSEASSVGGGASYEPSLEVYDSVHENWKTIGSMPMEFAVRLTVWTPNESVYCNGILYWITSARAYTVMGFEIRNKNWRELGVPMADRLEFAALVERNGKLCLVGGTSDAGACIWQLEESNNWRMIEKVPQELWAKLFGGKGRWGSINTRCVCIGGAMCLYRDLGSGMLVWRECAENGTKWEWHWIEGCGTIKGVHLQNFPIKGLLLHPYLASSNFLLNE

>StKFB14

MRRRSKTNRRRRVVEKKKKQFFFKDLSDELLIEILIRLPSSKEATLCKSVCKRWFALISSDNFRKISLTHNRNCDKKTLIPFTFVSIDYNYFHYLSDDTDLYVSEFSPENGFSRRVNSGFLYSNLPPVNYISLIESCGDLIYCSGGTSDRIDYYIVNVLTKQWFLLPRTPLESNTHFMSTSERVGFLIEPSSVDNAPCQYLVLMFISWGDSKFSIHVFSSKKGNWTRMVVTSPRNLNMLTRRTSIVACGRMFYTFTYERNDVVDCVLAFDPFTNDPAQFLSVIDFPPEARDKPCLTCKLGVCGGRLRFARIVLLPSRYLYPCISIWELEDDYRTGKWTLVHQRVPTDTVFRVPTLATKWVSVLTFHPYNEDLICFLVGNDHIVYNIQTDKLESSTLTSLFKKSLDVHVVPITRNWWPTSL

>StKFB15

MDLLPGLPNDIALECLIRLPLHQFSKAASVCTSWKTEIKHPLFRQRRKESGLTRPVFVLAQAMVTTIRKPYGITSLSSTQFYRLTLYDPERGCWYDLPPIPELIDGLPMFCRVVGVGSDVMVIGGCDPVNWRVMDSVFIYNFVSGSWRRGTDMPGGQRLFFGCASDSERFVVVAGGHNDEKNALRSALLYDVAEDEWITLPEMATERDECKCAFHRGEFHVIGGYPTHAQGQFHRSAEVFNSDTSRQWRLEEDFLGADTCPQTCIEGDDGRLYMCRDGDVVVKINATWKHVARLPGGVSNGAYLTAWQGKLLAVGNSILDEIHSSYELDVNSESKEKTWRKLDAPNEYCGHVQSVCWLEI

>StKFB16

MEIIPTLPYDIGLECLIRVPYYNFSSVTSVSRNWKLQIELPEFWRRRRATGSTRQVILMAQARIDPRLKLGSFKYSAFSVYKLTLYEPESGYWAELPPVPGISDGLPMFCQLVGVGLNLVVMGGWNPLTWEPSNAVFVFSFVSATWRRGADMPGCRRSFFGCASDSERTVYVAGGHDEEKNALKSAMAYDVARDMWVPMPDMASERDECKCTFFQDKFHVIGGYDTSMQGQFGTSAESFDPSTWQWDQVNEHFFESATCPRTCVEGGDGKLYLCRDGDVLALGKSTWQAVAAIPVELRSVAFVTAWRGKILMTGSMGFNEPHNTYVLDLQSYKWTKMDTPANFSGHVQSGCCLEM

>StKFB17

MPPVRARARGDTPATSEAPPLVAVVNSELYAIGYANMEVRKYDKKIKAWATIRRLPRAASMDDRGLAFRACGDRLIVIGEPIAMGVGPSEVSSSDALSGTIFDESDQDALCITVLSWVADHEIFANRDRMASVS

>StKFB18

MADLSLQSQLIPNLPDDIALQCLARVPRSHHPILSLVSKSWRCILSSTALYTTRSILRTTETFLYLNIRVNSTFHWYTLFHNLTFTNPEKPRKLFPLSSIPTKPIGPAFAVLGSRIYVIGGSIGEIPSNNVWVFDCRLNCWEMGPRMRIGREFAAAGVVNGKIYVMGGCVVDNWARSMNWAEVFDPMTGLWTALPSPIEVRDKWMHASAVVGEKMYAMADRGGVVYDVGGCEWGSVSKRLDLGWRGRAAVVGGVLYCYDYLGKIRGYDVKEDVWKELKGVDKGLPKFLCGATMVNFDDRLCVVWEGKGRGKEVDIMCAEIEVWKDEDGGLSGNILWSDMILVVPNGASIVQCLAVDL

>StKFB19

MEFSPLNRLPQDTLHQIFSHLTLREIIVSKCVCKCLNTTLSSPAFLHLISTQQPPLSLLALRPSHRTHTHTHNNSSSHCALHVFDTMLNYWFRFPLSFLPFRSHYPITSSHGLLYLWAEGPTSVSPPGNNSKTLIVCNPLTRQFKLLPQLGSAWCKHGSVLVGSPNQVLVLTELAAIYFSGSTTSNNWLKFSSNLPSKPRSPILISDTILALCDVGSPWRSQWKLFRSTVKDLQFSQQWTRLEKHEWGDIFDIMKRPRLLAGKNDKVLMIGGLKSSYSLHSTCSTILILRLDMESLEWEEAGRMPPEMFRYFQDSSKFKVFGGGSRVCFSGKRVGRLALWEENECGKGEWRWISGVPGNSDGLYRGFVFEARLNVVP

>StKFB20

MNYNRYSLTMENKKMIQSNQMDPKIWSRLPEDVLEHLLSFLPLKTFLKLRSTCKHFKTLLFSPPFISKSSSSSSSSSSSPFSSFFLLSHPQFPRQYPLFDTVHNNWRNLSLCISPVLPSSSSVLLSSSNGLLCFNSSNSSSFIITNVLARSSRVVKYPNLPFSFESVTLISSSNNGYKLFVMSAFGSSSQVFVYDSLVHSWSQFGGFDLILNENHHQEGVFHDGYLWFITPEPYFTVCMDLDNGVWKRSNFELPSEVTFARLVCDGDKKMFLVSGNGSNGISRSMKLWELNGDSKIWVEVENVPELICRKFLSVCYHNYEHVYCFWHKGLICVCCYSWPEILYYKVSRRTWHWLPKCPSLPDKWSCGFRWFSFVPELYAFV

>StKFB21

MESPPPPPMETSKRRAAATASIASLTNDNSVLPIELIFFEILIRLPVKTLLKMRSVSKSWISRISTPEFVKAHLNFSANNREFAHHRVLSIRSGSHIHDDGHITRWRYFRTFSLYAILYGESPCFPVELHNFGVSYNVLGSCDGLFVISKTWYDNDIENLFLWNPSIRKLSKLPYSGIDARKRRFAYGFGYIECQNDYQIVEIVASKPSYLIADISVYSLRNNSWKTIQEFPIISLPENVKFVKGKLHWITGGSSGNNATWFNPGDEKFGNVALPNPSGDTFNWKFVSSSGNLCMTCDYRNKIDVWIMKEYGLAESWTIVGSIPKFVNKVVRPIFISHNDEILLQDVSGLLWWYVSRDDGSFDRPEDQTRCEYDRGSELNLYIESLVSPNSP

>StKFB22

MTSERLTGEESLQQDLESLSVSKRLVRSVSQKLKKKNHRSGGEEEDDSKGISLRCLTLYGRGGGCKVGADTGDDLGDSCGRRRSNASEEGKGYNPICGNEETSVDCFSYGMREKFWRRSNRKSLELEAALQNKSMNVFLPDDILEMCLLRLPFISLVNARMVCKKWRNLTMTPRFWRMRQEGSFQSPWLFLFGVVKDGCCSAEIHAFDVSFNQWHKMNSEVLKGRFLFSVAGIHDDVYVAGGCSSLANFGKVDKSSFKTHKSVLVFSPLMRTWRKAAPMKHARSSPILGTYEISSDCLIIRNQQTRGDKRFYRPRVGGVSDVYEDPHRLSVRRQFRHSLDENEVTFFPNVKPYKFVKQKTEPSNKDQRRFLLIAVGGLGCWDEPLDSGEIYDSMSNKWTEIQRLPVDFGIACSGVVCNGLFYVYSETDKLASYDVEKGYWVRIQTSPFPPRVHEYHPKLICCNSRLFMLSVSWCEGEGQIGRRNKAVRKLWELDLMPLTWREVSIHPDAPMDWNAAFIADKNSIFGVEMFKIFGQVLDFLTVGDVTDAGINWSHISRNRLAQELDAASCLTKSMAVLHL

>StKFB23

MAIILEDEAPIHGDVLETILSHVPLVDLVPSSCVSKSWNRAVTSSLKCFNKPKPWLIIHTQCTRSPYDISVHGYDPRSNVWVEISQPSIKYVSALRSSHSNLLYMLSPSKLSFSHDAMNLTWHHVDAPRVWRTDPIVGYVGGSIVIAGGTCDFEDDPLAVEIYNNETNTWETCESMPAILKDSAASTWLSIATTGDKLVVAEKFTGVTYCFDPKTKNWSGPYELRPDPRIFHSIIGFANNRLILIGMIGDSENVAGVKIWKVNTENFECEELGEMPAALIQKVKSETYGVSSISVCLAGDYAYMSKSSEMAEEIVGCEFKNDGGLRWWSMKNEAAGDGNRSQRVVFSCSVIGLGDLQRAMLLENRKFTVKL

>StKFB24

MEVLAMLRQLIGQVKQLLELQASSSSSSSLVAVAPNISFHLQTPPLIHLPRCYFLNLDDNSAEDSCYNIIMTAGKSENLKMLEPGKPPPKKKGRKERNQGKVTGTSCSIENLDQQIWKEFPEDLFEAVVARLPVATFFRFRLVCRKWNSMLMSQSFSEQCAQVPQPQPWFYTITHENVNTGAMYDPTLKKWHHPTIPALPTKLIVLPVASAGGLVCFLDIGHRSFYVCNPLTRSFKELPARSVKVWSRVAVGMTLCGKSAGGGYNILWVGCDGEFEVYDSRNNSWARPGTMSSNIKLPLALNFKSQTVSIGSKLYFMRSDPDGIVSYDMVTGVWKQFIIPAPLHLSDHTLAECGGRIMLVGLLTKNAATCVCIWELQKMTLLWKEVDRMPNIWCLEFYGKHVRMTCLGNKGLLMLSLRSRQMNRLVTYDFSSREWMKVPGCVLPRGRKRQWIACGTAFHPCLTALA

>StKFB25

MLYLPNEIIFEILLRLPVKSIIKFKSVSKSWLSLLSSPRFINTHLNFSKNNHKNVPQKLLLLTPNQNLSKKQYTLFSSISETIVRVDLDYPVKSPSCVTQFIGSCDGLICFSVENSLILWNPSTRKWKKIPKESIFMNQDYYCTYGFGYDEFNDDYKLILVYSSKIKNIGYNEVKVYSLKTNSWKRIKGFVNGYVYSNSGVLMNGIIHWDSRPHRDFNGNDSKIVYFNLVSEKLGKLDLPSYDENEDVVWDLMSSKESLFGFCHSESQGAVDIWVMKEYGIKESWIKFASVPYYVVPGIFDTSMFINEDGEVLLIDGERLVLFNTRNNIYKDLRIHIPDTRRRIDMATYNETLVSPVFDDEDGCEFW

>StKFB26

MTALIEGLPDAVAIRCLARVPFYLHPKLELVSHSWRAAIQSAELFKARQEVNSSEEFLCVSAFEPENLWELYDPTHDLWITLPILPSNISHFARFSVVSTAGKLFVLGGGSDAVDPLTGDQDGIFATDKVWSYDPATRAWNPRASMLVPRAMFACCVLDGKIVAAGGFTNRRKSICNAEIYDPETDVWVQLPDLHHAHNSACSGVVFGGKVHVLHKGLSTIQVLENFKQGWIAHEYSWLQGPMTVVRENLYVMSNWFIYKQEGELRRMIVSASDFRRRIGYAMIGLGDDIYIVGGVNGPDYWNCSVKVLSDVDVLTLGSERPAWRKVAPLTRCKGTILGCTQLRI

>StKFB27

MATSSSCSSPYTNGSSPITTIAQDHLFSILLLLPLDSIFCFALTCKKFRSLTYSDSLWESLCRRDWGNSTIDALRSFAVDGKQQQFPWKKLYQQIYQLDSVYCRRLLTHPQGGEELLLPRPRASHSLNFVSGCLVLFGGGCEGGRHLDDTWVAYAGNDFKRILEWNKIESGVPSGRFGHSCVVIGDYLVLFGGINDHGARQNDTWIGQVAVHDAFGITLSWRLLDVGSIVPPPRGAHAACSMDKRRMLIHGGIGLSGLRLGDTWVLELSENLHLGVWQEIVTHPSPPSRSGHTLTPVGGNQTILFGGRGLGYEVLNDVWLFDTSDGHWRWVQLLFDLQNIPQGLTLPRVGHSANLIIGGRLLIYGGEDSYRHRKNDFWVLDISSVTSIMQSGSPPSPDRSMTKLWRRLKSKGDNPCGRSFHRACVDPSGRNLYIFGGMVDGLLNPAESSVLRFDGELFLVELLLQC

>StKFB28

MADQNNNEDTVINCNLPKDAVERILVGLPVRSLLQFKSVCKSWYNFIKSRNFIKIYRNHQISRPPSALISYKKNNVSSFWPDFFSLNLISNIEQYNFPINQQLFLPFAIDDAKDWANFEFCYNGLICLTKDIYTSPVVILWNPASRRYKCINIPSECSRLTYLKLGFHQQSNDYKILKVPFQTRSDDESTKMAWVYTLSSGSWKTVPFSLLSLTVAWGPQISVDGFVYWLGTRGVEYIISFDLIKDEFKLMDVLDDRGFDRQLVRRKLMVLRGSLAMIVSVEDCTRDTEIWMLVKGNSRLQPYSWTKKFILEPFSKETTTLGMWNDHKLLVVVSQTRPHALRQVYSYDLVTKEMEYFHMPKEQDLASFAAVRGCYFESLELEDGSGRRY

>StKFB29

MDLIPGIPNEIALECLIRLPVDQFSKAASVCKKWNGEITLPEFRRRRKVSGLTRTVFAMVQSMVATVKKPEGDTALFSTQVYRLSVCDPENGSWYDLPPIPELIDGLPRFCRIVGVGSDLVVIGGCDPDTWRVMDCVFVYNFISGSWRRGADMPGQQRSFFGCASDSNRMIVVAGGHDDEKNAMKSALSYDVVKDEWITLPDMVMERDECKVVFHKGKFHVIGGYPTWAQGHFENDAEVFDTATWQWQLEDDFLSVINTSPHSCIEGDDGKLYMCRGGDVAVKEDPTWRKLTRLPAGITSVAYLTSCRGLPAGITSVAYLTACQGKLILVGRGQFDELYSIYALDLDVESDGKVKKWTKVETSDEYSGHVQYGCCLEI

>StKFB30

MQILQWLFKNANETNFNTNSSKKQVVTDEEVKSQGIVLFAISRKKRNRAGKSANSCCCNILNLFNTLRRKDIAKDYFYSTIRLKRLESSRRKQQFVDCMKMKKQSLTRGLSFRRKSDSATAKVLPVSDGHKEQNFSADKKEKANGDKIKTVSKMKELIRWAAAAKSQKGGKYFGQKVLHLRDRSDLKAVPDDDDQLSYDSPKISFRWDVDQNYSAISVTKNHQSIKNSPLNTMVHIDQCLAATRKGNWVTTDSEYLTEKILSYLPIRSIIVASSVCKIWNSIITSSSFTTKISATKKQPWLFLCGQNSIFYNNNQPFAYDPDANEWISLPTSTLLSQDFFIGSNGFFFATTSENFSFKPIFNGIWVQTSPLRFSRCNPLVGVYNEGLNPRFIVVGGVRFVGGLVDVEDRLAVEIYNPNLDYWELCPPLPADFRSGNSSQWLCSALLRDKFYYVFGIYSCFITCFNLDEHLWSEVQTLRPPGILFSFLVTCQDCLVLGGLCNSPNGPNFVLWKVDEKTMEFSELATMPQALMYCLFDSDEDDKFASLKCVGLGNLVYVYNEEHLKNYPACVCEFSNDFGMCSWRKLPNLPPNASKFHRVISFCSNVSLDSILVGARI

>StKFB31

MLDLNKIEPEEGKLDLDLRRVNYDLDDKSHLRYGDWVTFKSHSSKKVRISRPESPRGENSDRQPQDADYPSLSYELESEILSRFPRWEYWKLSLVNKRCSMLLKSGEIFEIRKENGFKEPSVYMLASGETNWWTFDREFNSRRKIPDLPSDVCFTFGDKESLCAGTHLLVSGREIDGLVIWRFELATNCWYKGPSMVNPRCLFASATCGTSAFVAGGVGIMANSEVYDTAEKYNPDSRSWDPLPRMKRKRKLCSGCYMDKKFYVIGGRNENGELTCGEFFDEGKNKWELIPDMLKDDPVLTCHSPPLIAVVNNELYSLEASSNQLKLYLKKTNTWKMLGQVPVRADSNRGWGIAFKSLGNELLVIGAASSSASYSGNSMAIYTCCPDPDAIELQWQPLDSGRNRLSSFILNCSVMVA

>StKFB32

MPNDITFNILLKLNVKSLLRFKCVCKSWCSLIEDPQFIKQHYDMSKKDVNRHKFFLTGGEWENKDDYFFSIDTPLQHDSAASLIESLIPGINHLSSVSFVSSSNNGIILMVFPYDFIILWNPAIRELRKIPSSIPKIKKSGRKRFPAIYGLGYVSSIDDYKIVRVGGKDNSNGHFEIDLFSTRDNSWKLIGKFPPYNFFREGDIVSIDGIVYMIVMTNLSESVNESTILSFCLEKEQFEEVLFPDRILRFQNPILHVLEENLCLTRMHDLASRDFEVWHMKKDGSMSTTWSKILTIPSMYLWTLLEAREINEN

>StKFB33

MSDETADCSLTGDVIYEILLRLPVKSLLRFRSVSKSWYCYISSRDFIQMHRRQPVHEKLLRVSDRGPRDIPTISFFSLDPKITTVVVDDEIDELGDFDSSPDPSVVVVDLPFPCTPDDEVRVVGSCNGLLCVHFNRSSSIILWNPATRKYKLLESPDCVSFYTDPFFPCIMLGFVPQTNDYKVIKLPSSSKNPKVWVYSLNSDSWEEIEAPILHGLLPKGGSAVILNHCLYWLSYSNDDDNELDIITCFGLYNQVFTRIKLPNLDTASNLTIQKLVILKGCLSMITYSGNGIAVNNYEVWVMSEQQGAAEFWTKQFAFSSFSNLARPVGSWRKGELLLGYPNGLSLELISYNPYTKRTHSFQKRTSEDRFKVVNYVESLESVEGS

>StKFB34

MASRRNLKRKSIEDEDLVFPFDELNQDLLERVLSWLPTSTFLRLSSVSKRWKSAANSPVFHHACSEIPSREPWFYMVDSSQSVEFKNQQHFVYDSAEMNWKFLTYPSNFLEENQLNQSNFLPVAASGGLLCFHNGENGEFLICNPVTSSYRKLPLLDYSDTLSAIGMISTQESYKLFLIFGEIPNLSFRIYDSTTDSWKDSAILSKKSPTCPAAQSCSTDDEEEEDDDRMLYFLSKCGNVVGTEIQKNPCKQYSSITTTTKGDGQEILCFLNSSGKVVACNLTEKHFFEYPRLLPLHHEYSIDLVECGGELLAVVLSEFLETASLRIWKFDENEWAWNQVLAMPSAISHEFYSKKVDINCTGSSEHMFVCISNSGSTDDESCRYFLCKLAENEWTELPACSGKFSCAFSFQPRIEASV

>StKFB35

MNNSATITVAAADTAAGAAVKRRKKLHQHHQYESLILGLPDDIAQICLSHVQPSTLYSVCQSWRRLVYSPSFPPFLSIYSLLKPSKSEENSVQFANFDPISAKWNLLPPPPLNPPLRLLLRHRSFISRHLPIQSVSVSGNFILLAATTDPLLPALSRPLVFNPLTRKWTSGPRFKTPRRWCVAGASQGVVFVASGIGSSYNPEVARSVGKWDLKSNCTKYSCNHHDRNKVWKWEKMSGLKDGKFSREAIEAVGWRGKLCMVNVKGDAAKEGIIYDVGSDTWQEMPEGMLVGWRGPVAAMEEEVIYTVDESKGALRKYDPGSDSWIMILENEMLKGAQHMAAAGGRVCVVCGGGDGIAVVDVTAEPPSLVVVETPVGFQVLDVHILPRMNQLDS

>StKFB36

MSVLIEGLPDAVALRCLARVPFYLHPKLELVSHSWQAAIRSGELFKARREVNSSEEFLCVCAFDPDNLWQLYDPMRDLWITLPVLPSNIRHLAHFGVVSTAGKLFVLGGGSDAVDPLTGDQDGSFATDEVWSYDPVTREWSLCASMIVPRAMFSCCVFDGKIVVAGGFTNCRKSICKAEIYDPEKNVWDPIPDLHHTHNSACSGVVIGGKVHVLHKGLSTVQVLENVKQGWTVHEYGWLQGPMAVVRGELYVLSHWLIYRQERETRKMVVSASEFRRRIGFAMIGLGDDIYIVGGVIGPERWNWDIKLLSDVDVLTLGNERQVWRQVAPMTRCRGTVLGCTLMRI

>StKFB37

MALPSSTTVTNQESQLQTLNPTIIPGLPNDLAAVILVFVPYSHHSRLKSICKSWKQFFSSKTIISLRQKHLPLSTLSPLLCIFPQDPLIASPYLFDPRNLAWSPLPPMPCNPHVYGLCNFTSICIGSHLYVLGGSLFDTRSYPLDYPCPSSSAFRFDFGTSSWETLAPMINPRGSFACAAAPNLDKILVAGGGSRHTMFGAAGSRMSSVEMYDIGKDEWVALDGLPRFRAGCVGFFVGNGEEREFWVMGGYGESRTVSGVFPVDQYYRDVLVMEMKNGGKWRELGDMWEEGERWKLGKIVVVEDVPSEAPAVFMLDRGDIFSYIMASNSWIKETSLPRKTSDESSVGFVALDGELHVISHLNGVKSKECQRLRQQKRSTMLVQIYHPRTKSWRAVTTRSPFQHPLDFKTAVMCTIRL

>StKFB38

MPNDIILNILLRLPIKSLLRFKCVCKSWRSLIEDPQFIKHHYDMSKSDVNRHKIFLTGGICDNMDNYFYSVDAPLQYDSVVSVLETPIPGINTLSRVSFISSSSNGIILIVFPYDLIILSNPATGESRKIPSPIPKKKKLERRQFPAIYGFGYVSSIDDYKIVRVGGKDNSHGHFEIELFSTKSNAWKLLGKFPPNSFYFEGGIVTIDGIVYMIEMADLSRNINRSTILSFCLENEQFQDVLFPDQIQKFQDPILYVLGENICLTRMHDLARRDFEVWHMIKDGSMNNIWSKILTIPSMHCGRCLRPVSLMKINGYIMLLKHKGDFEVYNSNGEQIEIVEVPGLESSLFFNCIVPYVESLFTPKRKRI

>StKFB39

MNQNEFTELVPGLPEDIALECLTRLHYSTHGVASRVCRRWCRILQSKAFYYHRKQTGFTHKAACLVQALPSPVESKPTGQPRYAISVFDLVSGIWDRVDPIPKYPDGLPMFCQIATTEGKLIVMGGWNPASWNPIKDVFVYDFMTRRWNQCKDMPEARSFFAMGAAGGKVFIAGGHDESKNALSSAWVFDISSNEWTELPRMSEERDECEGVIIGSDFCVVSGYDTETQGRFKSSAELYELSTGQWRRVEDAWRSSQCPRACVGVGKNGNLTCWAESDPNVKVGACGVDLGDRTLVTGSAYQGAPHGFFFVENNKKGQNSKLTKINVPDEFSGFVQSGCCVEI

>StKFB40

MKRGRRCKLFVCEEIIINILNRLPLKSLEKFKCISKNWRKYIAEIYRRRFQWPEPYLLGFFCLEKRSQSCFFYSSKESPLVIGSSLDESVNFIGEKVYIVASSNGFLLCNKLRSRHRVYYVYNPATRQRLDVPKTPISMDDPYIGFTCKVGEDHGIVSFTIVRYVISEHYQSSITIESFSSEINVWTDYKLIVDVPHALYPSLDGTSLSSAGVVDGIFFWLNYTLITIYDSVNKCFWALELPENLFIYYPRSCCLGLSGGELCFASNRWTTTITCWRLNNFPSRDNAEWVIKYVINVATIIKKYPKYFGVGDGFAKVQNMVFHPALPHILYLQIKDKVISYDVKTRKTELVYDFGEAWRKTKCYKLFSYEWPQWPRLL

>StKFB41

MKHLRQCNLFIHEEIIINILNRLPLKSLARFKCISYNWQKCIAEIYRCRLGFPEPYLIGFFCVEKRLRSRFFFTSKESPLLIGTNLDKSIDFIGERVYIVASSNGFVLCNRLRSRHRVYYVYNPATRQRLDVPKTLIHMKDPYVGFTCKVDEDHGIVSFTIVRYVIRKRYQSSITIESYSSETNVWIANNVIHDLLYPLQPSRDENYLSSRCVIDGVFCWIDNFGQGMTVYDSVKKCFWGLTYPQPGCMIYPGFHFLGLSGGEVCFASKGWTIPCWRLNNFPSRDAEWVWKYNVDVVAIIEKCGEDFGLGGGNVQNIVFHPVFPDILYLQINGKVISYDVKTSTVELVYDFGEAGRKTKHYKLFSYEWPQ

>StKFB42

MKSCFPNNQHPPPHHPTPIGTTTILPTGTTIAELPDDLLLECLSRVTHSSLPSLPLVCCRWSLLLDSPTFHLLRHRHNLLRLTLFAVSVSDGALCTASYRLNNDCSWKICSFTPANDPVFEHGCFYSLFSHSRLSVIGRKIYVIGRTAMLRCDTWTGLVVPRQGPVFPRKKFAAAVVGGKIYVAGGCARSAAVEEYDPTSNTWSVVAKAPRKRYGCVGASVDGVFYVIGGLKLGGASGNEMLVARGSRASDAAHVYASSMDLYDTVNGVWLKTRSVPGGGCVVAALELSFWKFNGSRKSTGFGDWCRIKSPPLPAQVRLDSTVRFSCVGIGEKVVLVQVNGCIDDLLRRSGRIERGLKEGLVLVYDCVAGEWSRAADLPEVIRRSACVCVEC

>StKFB43

MDSVFVSNPRNNYSVPHDIIIDILTQLPVKSLIRFKGVCKSWYSLIKDDNFIKQHYDTHQNCQKYFVVCRRHKISTHYTMELDSNSIASLVAPPVPIDQSRKAFNEIQYCSCNGILLITYANDIIIMWNPATRESRRIPKCKSGGLYNFCYFPRIESYKIFRLGPVVFNGDKDEMDIDIFSTKSNKWKTVGIFPPDYYFEGTSIVMSDGIVYMMAERKENENCTILRFCLDKEEFQEELLFPNTIPSEIHVVGEKLCLIGSLMSNHKIHEFWLYMMKTNSWNKILAIRLPSKSCLKPLSFIKDGGIMFKNYMSKYVFKAYNSTTHKLEKVNVAGLEGHNFKEVVTYVETLSSPFL

>StKFB44

MDSTQNVPHDIIIDILAQLPVKALIRFKGVCRSWYSLIKDNKFIKQHYDTHKNCQKYFMTCRRRNMKNQFNYYHYTMDVPQLDSTSTISLVESPVPIDRSRMVLGNTQYFSCNGILLIITYANDIVLWNPATKESRRIPCPIRSKSGGLYNFCYFPSIDSYKIFRLGREVFNDDKNDMDIDIFSTKGNSWKSVGMFPSNYDFLDSSIVMSDGIVYMMAKRIGNLVSSTILRFCLEKEQFEEELLCVDTIPGRIRILYSVGEKLCLISSSGDNNEVREIWLYMMTTNSWSKILTIPLTLRPLSFMEDGGMMFQKFNRSGFVFEAYNSTTHKFEQVNVTEIEGLNLFNRVVTYVETLSSPNS
