# Supplementary material for: Genome-wide identification, evolutionary and functional analyses of KFB family members in potato
Source: BMC Plant Biol. 2022 May 2;22:226. doi: 10.1186/s12870-022-03611-y (PMC9063267; doi:10.1186/s12870-022-03611-y)
Supplement: Supplementary file 3 — Additional file 3 Fig. S1. Gene duplication analysis of potato genome. The local database of potato protein sequences was established by Makeblastdb program. And pairwise comparisons were made between potato protein sequences by Blastp with E-value ≤1e-10. The gene duplication analysis result was obtained by duplicate_gene_classifier program provided in MCScanX software. Singleton: single copy genes; Proximal: adjacent but discontinuous repetitive genes on the same chromosome; Tandem: tandem duplications; WGD or segmental: whole genome duplications or segmental duplications; Dispersed: dispersed genes. Fig. S2. Sequence logos of conserved motifs in StKFB proteins. The 20 conserved motifs of the putative StKFB proteins were identified by MEME software v5.3.0. Fig. S3. The correlation analysis between the expression patterns of StKFBs in diverse potato tissues (a), in potato plants with different treatments (b) and in three colored potato tubers (c). ﻿The correlation between the expression levels (FPKM values) of StKFBs was analyzed by Pearson’s correlation coefficient and plotted using the corrplot package v. 0.92 (https://cran.r-project.org/web/packages/corrplot/). Fig. S4. Dissociation curves of primers for qRT-PCR. Dissolution curves were obtained by heating the amplicon from 60 °C (5 s) to 95 °C (50 s) on CFX96 PCR System (Bio-Rad, USA). Fig. S5. Comparison of the expression levels of the 9 selected StKFB genes determined by qRT-PCR and transcriptome sequencing. The boxplots were plotted using tidyverse v. 1.3.1, cowplot v. 1.1.1, ggplot2 v. 3.3.5 and ggsci v. 2.9 packages in RStudio. Values are means ± SD of three replicates in each experiment. Bars with different lowercase letters represent significant difference at p < 0.05. Fig. S6. Conserved domain analysis of StKFB01, AtFKF1, OsFKF1 and StKFB27. The conserved domain analysis was conducted by Conserved Domain Search tool (https://www.ncbi.nlm.nih.gov/Structure/cdd/wrpsb.cgi) in NCBI. [file 12870_2022_3611_MOESM3_ESM.docx]

**Additional file 3**

**
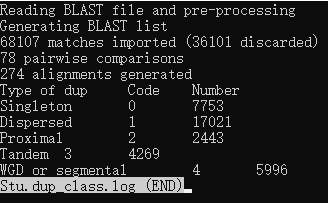
**

**Figure S1** Gene duplication analysis of potato genome. The local database of potato protein sequences was established by Makeblastdb program. And pairwise comparisons were made between potato protein sequences by Blastp with E-value ≤ 1e-10. The gene duplication analysis result was obtained by duplicate_gene_classifier program provided in MCScanX software. Singleton: single copy genes; Proximal: adjacent but discontinuous repetitive genes on the same chromosome; Tandem: tandem duplications; WGD or segmental: whole genome duplications or segmental duplications; Dispersed: dispersed genes.

**Figure S2** Sequence logos of conserved motifs in StKFB proteins. The 20 conserved motifs of the putative StKFB proteins were identified by MEME software v5.3.0.

**a**

**b**

**c**

**Figure S3** The correlation analysis between the expression patterns of *StKFBs* in diverse potato tissues (**a**), in potato plants with different treatments (**b**) and in three colored potato tubers (**c**). ﻿The correlation between the expression levels (FPKM values) of *StKFBs* was analyzed by Pearson’s correlation coefficient and plotted using the R package “corrplot.”

**Figure S4** Dissociation curves of primers for qRT-PCR. Dissolution curves were obtained by heating the amplicon from 60°C (5 s) to 95°C (50 s) on CFX96 PCR System (Bio-Rad, USA).

**Figure S5** Comparison of the expression levels of the 9 selected *StKFB* genes determined by qRT-PCR and transcriptome sequencing. The boxplots were plotted using tidyverse v. 1.3.1, cowplot v. 1.1.1, ggplot2 v. 3.3.5 and ggsci v. 2.9 packages in RStudio. Values are means ± SD of three replicates in each experiment. Bars with different lowercase letters represent significant difference at p < 0.05.

**Figure S6** Conserved domain analysis of StKFB01, AtFKF1, OsFKF1 and StKFB27. The conserved domain analysis was conducted by Conserved Domain Search tool (https://www.ncbi.nlm.nih.gov/Structure/cdd/wrpsb.cgi) in NCBI.
